# Use of MALDI Mass Spectrometry Imaging to Identify Proteomic Signatures in Aortic Aneurysms after Endovascular Repair

**DOI:** 10.3390/biomedicines9091088

**Published:** 2021-08-26

**Authors:** Matthias Buerger, Oliver Klein, Sebastian Kapahnke, Verena Mueller, Jan Paul Frese, Safwan Omran, Andreas Greiner, Manuela Sommerfeld, Elena Kaschina, Anett Jannasch, Claudia Dittfeld, Adrian Mahlmann, Irene Hinterseher

**Affiliations:** 1 Berlin Institute of Health, Vascular Surgery Clinic, Charité—Universitätsmedizin Berlin, Freie Universität Berlin and Humboldt-Universität zu Berlin, Hindenburgdamm 30, 12203 Berlin, Germany; matthias.buerger@charite.de (M.B.); sebastian.kapahnke@charite.de (S.K.); verena.mueller@charite.de (V.M.); jan-paul-bernhard.frese@charite.de (J.P.F.); safwan.omran@charite.de (S.O.); andreas.greiner@charite.de (A.G.); 2 BIH Center for Regenerative Therapies BCRT, Berlin Institute of Health at Charité—Universitätsmedizin Berlin, Augustenburger Platz 1, 13353 Berlin, Germany; oliver.klein@charite.de; 3 Center for Cardiovascular Research (CCR), Institute of Pharmacology, Charité—Universitätsmedizin Berlin, Freie Universität Berlin and Humboldt-Universität zu Berlin, Hessische Str. 3-4, 10115 Berlin, Germany; manuela.sommerfeld@charite.de (M.S.); elena.kaschina@charite.de (E.K.); 4 Department of Cardiac Surgery, Herzzentrum Dresden, Medical Faculty Carl Gustav Carus Dresden, Technische Universität Dresden, 01307 Dresden, Germany; anett.jannasch@tu.dresden.de (A.J.); claudia.dittfeld@tu-dresden.de (C.D.); 5 University Center for Vascular Medicine, Department of Medicine—Section Angiology, University Hospital Carl Gustav Carus, Technische Universität, 01307 Dresden, Germany; adrian.mahlmann@uniklinikum-dresden.de; 6 Medizinische Hochschule Brandenburg Theordor Fontane, 16816 Neuruppin, Germany

**Keywords:** aortic aneurysm, EVAR, endoleak, proteomic signature, MALDI-MSI

## Abstract

Endovascular repair (EVAR) has become the standard procedure in treating thoracic (TAA) or abdominal aortic aneurysms (AAA). Not entirely free of complications, a persisting perfusion of the aneurysm after EVAR, called Endoleak (EL), leads to reintervention and risk of secondary rupture. How the aortic wall responds to the implantation of a stentgraft and EL is mostly uncertain. We present a pilot study to identify peptide signatures and gain new insights in pathophysiological alterations of the aortic wall after EVAR using matrix-assisted laser desorption or ionization mass spectrometry imaging (MALDI-MSI). In course of or accompanying an open aortic repair, tissue sections from 15 patients (TAA = 5, AAA = 5, EVAR = 5) were collected. Regions of interest (tunica media and tunica adventitia) were defined and univariate (receiver operating characteristic analysis) statistical analysis for subgroup comparison was used. This proof-of-concept study demonstrates that MALDI-MSI is feasible to identify discriminatory peptide signatures separating TAA, AAA and EVAR. Decreased intensity distributions for actin, tropomyosin, and troponin after EVAR suggest impaired contractility in vascular smooth muscle cells. Furthermore, inability to provide energy caused by impaired respiratory chain function and continuous degradation of extracellular matrix components (collagen) might support aortic wall destabilization. In case of EL after EVAR, this mechanism may result in a weakened aortic wall with lacking ability to react on reinstating pulsatile blood flow.

## 1. Introduction

Defined as a dilatation of the aortic wall by 1.5 times of the physiological diameter, aortic aneurysm is a common disease with an age-dependent prevalence of 8% and is a leading cause of death in men >65 years old. If left untreated, the risk of further dilatation and rupture increases. With a mortality of up to 80%, aortic rupture is an acutely life-threatening event [1].

The aortic wall consists of a three-layered structure. On the luminal site, a single layer of endothelial cells is forming the tunica intima. The tunica media is formed by adjacent vascular smooth muscle cells (VSMC) and structural proteins, such as elastin and collagen. The outermost tunica adventitia is composed of mostly fibroblasts and collagen fibers [2]. The formation of an aortic aneurysm is considered a multifactorial process caused by genetic and epigenetic alterations supported by behavioral risk factors such as smoking and atherosclerotic degradation. However, the exact pathophysiology remains to be elucidated. Histologically, aortic aneurysms are characterized by inflammation, VSMC apoptosis, extracellular matrix (ECM) degradation and oxidative stress [3,4]. This results in a weakened and instable aortic wall which is no longer able to withstand luminal blood pressure.

To prevent aortic rupture, aortic aneurysm repair should be considered from a threshold diameter of 60 mm or 55 mm in thoracic (TAA) and abdominal aortic aneurysm (AAA), respectively (depending on localization, configuration, and patient gender). Furthermore, rapid aneurysm growth is an additional risk factor for aortic rupture requiring aortic repair. Endovascular stent grafts became state of the art in the treatment of TAA and AAA [5]. Several studies have shown an early survival benefit of patients treated with EVAR compared to conventional open aortic repair for elective surgery in infrarenal AAA in short-term follow-ups [6,7,8]. Nevertheless, the emergence of late complications after EVAR leads to a loss of the initial survival benefit during long-term observations [9,10]. The most common complication leading to secondary intervention is persisting perfusion of the aneurysm sac, a so-called endoleak (EL) [11,12]. As a result of persisting EL, the risk of aortic rupture is increased [13].

The underlying pathophysiological changes in the aortic wall leading to EL and rupture after EVAR are unclear. Findings of structural atrophy due to a significant thinning of the aneurysm wall layers accompanied by cell deficiency suggest a weakened aneurysm wall after EVAR. Cell-depletion supported by alterations of extracellular matrix (ECM) components, such as collagen, may result in a status of instability [14]. Furthermore, new insights into the role of VSMC as the predominant existing cell type in aortic walls underline their importance for aortic wall integrity. The exact sequential pathophysiology of AAA formation remains uncertain, but VSMC contractile and synthetic phenotypes are considered to have an impact on aneurysm formation [15]. While the synthetic phenotype provides manufacturing of ECM, the contractile properties of VSMC encompass mechanical force distribution by regulating their linkage to ECM components [16,17]. It has been shown that mutations in genes, encoding for contractile proteins of VSMC, such as smooth muscle actin (ACTA2) and smooth muscle myosin heavy chain (MYH11), predispose for hereditary TAA and aortic dissections [17,18]. Recently, evaluations of VSCM contractility suggest both an impaired contractile function in AAA patients and patients formerly treated by EVAR [19]. Therefore, pathophysiological changes of ECM components accompanied by alterations of VSCM properties are of particular interest to understand mechanisms leading to complications after EVAR.

The unraveling of molecular changes often remains hidden due to tissue heterogeneity. Proteomic methods have been successfully used to characterize pathophysiological processes in various diseases [20,21]. In current studies, fluid-based proteomic approaches such as liquid chromatography are combined with mass spectrometry after tissue microdissection to discover new disease-related markers in muscle tissue [22,23,24]. However, obtaining a sufficient amount of material is labor-intensive and provides little insight into the actual spatial distribution of pathophysiological regions.

Recently, matrix-assisted laser desorption or ionization mass spectrometry imaging (MALDI-MSI), a tissue-based technology for analyzing human specimens, entered the field of diagnostics for a variety of diseases [25,26]. Several studies have demonstrated the advantages of high resolution MSI data in microdissected tissue sections while preserving spatial specificity with accurate protein assignment [27,28,29]. Due to long processing time for both microdissection and mass spectrometry and the higher costs, these promising techniques are not well suited for large scale studies. In contrast, spatially distinct peptide signatures obtained from MALDI tissue imaging data can be acquired in a shorter time frame, a larger sample cohort, and at a lower cost [30,31,32]. Combining a mass spectrometric technique with conventional histological evaluation on a single tissue section allows the analysis of a variety of molecules. Furthermore, by preserving the spatial coordinates in the analyzed tissue sections, it generates a unique molecular intensity map and thus allows conclusions about location-related alterations in specific regions of interest. Referring to aortic diseases, Mohamed et al. emphasized the potential of the MALDI technique in a proof-of-concept study to provide useful information for underlying pathogenesis in aneurysms of the thoracic ascending aorta [26].

The aim of this pilot study was to investigate the feasibility and potential of using MALDI-MSI combined with univariate statistical analysis to differentiate between TAA, AAA, and EVAR and to discriminate remodeling processes in aortic wall specimens after EVAR. Alterations in proteomic signatures after EVAR might be used to gain insights into the underlying pathophysiological remodeling leading to complications such as EL or aortic rupture.

## 2. Experimental Section

The study was approved by the institutional ethic review board at the Charité Universitätsmedizin Berlin on 7 July 2020 (project identification code EA4/108/20) and written consent was obtained from all patients.

### 2.1. Patient and Sample Cohort

Human aortic tissue samples were obtained during elective open aortic surgeries for TAA, AAA, or EL, and aneurysm sac enlargement after EVAR, respectively. Specimens were removed intraoperatively from the area of largest diameter of the aneurysmal formation in the thoracic or abdominal aorta. Subsequently, aneurysmal aortic tissue sections from TAA (n = 5), AAA (n = 5), and EVAR (n = 5) were transported to the laboratory for further processing and preparation of formalin-fixed paraffin-embedded (FFPE) tissue sections.

### 2.2. MALDI-MSI

FFPE tissue samples were prepared for MALDI-IMS analysis as previously reported [33]. Briefly, all FFPE tissue sections were 6 µm thick, cut by microtome (HM325, Thermo Fisher, Bremen, Germany) and mounted onto conductive glass slides coated in indium tin oxide (Bruker Daltonik GmbH, Bremen, Germany). Sections were preheated to 80 °C for 15 min before deparaffinization. Paraffin was removed in xylene, and tissue sections were processed through 100% isopropanol and successive hydration steps of 100% ethanol followed by 96%, 70%, and 50% ethanol, each for 5 min. Sections were fully rehydrated in Milli-Q-purified water (MilliQ-water). Heat-induced antigen retrieval was performed in MilliQ-water for 20 min in a steamer. After drying slides for 10 min, tryptic digestion was performed. An automated spraying device (HTX TM-Sprayer, HTX Technologies LLC, ERC GmbH Riemerling, Germany) was used to deliver onto each section, 16 layers of tryptic solution (20µg Promega^®^ Sequencing Grade Modified Porcine Trypsin in 800 µL digestion buffer; 20 mM ammonium bicarbonate with 0.01% glycerol) at 30 °C. Tissue sections were incubated for 2 h at 50 °C in a humidity chamber saturated with potassium sulfate solution, then the HTX TM Sprayer applied 4 layers of the matrix solution (7 g/L a-cyano-4-hydroxycinnamic acid in 70% acetonitrile and 1% trifluoroacetic acid) at 75 °C. MALDI imaging was conducted on the rapifleX^®^ MALDI Tissuetyper^®^ (Bruker Daltonik GmbH, Bremen, Germany) in reflector mode with the detection range of 600–3200 *m*/*z*, 500 laser shots per spot, a 1.25 GS/s sampling rate, and raster width of 50 μm. FlexImaging 5.1 and flexControl 3.0 software (Bruker Daltonik GmbH, Bremen, Germany) coordinated the MALDI imaging run. External calibration was performed using a peptide calibration standard (Bruker Daltonik GmbH, Bremen, Germany). The matrix was removed from tissue sections with 70% ethanol after MALDI imaging, and sections were stained with hematoxylin and eosin for histology. Subsequently, aortic wall layers were annotated in QuPath software [34] and transferred into SCiLS Lab software (Version 2019c Pro, Bruker Daltonik GmbH, Bremen, Germany). In general, we stuck to the previously published standard operation procedure by Ly et al. [35].

### 2.3. Protein Identification by Electrospray Ionization Tandem Mass Spectrometry

Protein identification for peptide values was performed on adjacent tissue sections using a bottom-up nano-liquid chromatography electrospray ionization tandem mass spectrometry approach as previously described [33]. Similar to their preparation for MALDI-MSI, sections were preheated to 80 °C for 15 min before deparaffinization. Paraffin removal, antigen retrieval, and tryptic digestion were carried out as for MALDI-MSI. After incubation at 50 °C in a humidity chamber saturated with potassium sulfate solution for 2 h, peptides were extracted separately from each tissue section into 40 μL of 0.1% trifluoroacetic acid and incubated 15 min at room temperature. Digests were filtered using a ZipTip^®^ C18 following the manufacturer’s instructions, and the eluates were vacuum concentrated (Eppendorf^®^ Concentrator 5301, Eppendorf AG, Hamburg, Germany,) and reconstituted separately in 20 µL 0.1% trifluoroacetic acid, from which 2 µL were injected into a NanoHPLC (Dionex UltiMate 3000, Thermo Fisher Scientific, Bremen, Germany) coupled to an ESI-QTOF ultrahigh-resolution mass spectrometer (Impact II™, Bruker Daltonic GmbH, Bremen, Germany). The peptide mixture was loaded onto an Acclaim PepMap™ 100 C18 trap column (100 µm × 2 cm, PN 164564, Thermo Fisher Scientific, Bremen, Germanyand calibrated with 10 mM sodium hypofluorite (flowrate 20 µL/h) before separation in an Acclaim PepMap™ RSLC C18 column (75 µm × 50 cm, PN 164942, Thermo Fisher Scientific, Bremen, Germany) with an increasing acetonitrile gradient of 2–35% in 0.1% formic acid (400 nL/min flow rate, 10–800 bar pressure range) for 90 min while the column was kept at 60 °C. Released charged peptides were detected by a tandem mass spectrometer using a full-mass scan (150–2200 *m*/*z*) at a resolution of 50,000 FWHM. The autoMS/MS InsantExpertise was used to select peaks for fragmentation by collision-induced dissociation. Acquired raw MS/MS spectra were converted into mascot generic files (.mgf) for amino acid sequences using ProteoWizard software [36], and used to search the human UniProt database using the Mascot search engine (version 2.4, MatrixScience Inc., London, UK) with the significance threshold of *p* < 0.05 and the settings for trypsin as the proteolytic enzyme; a maximum of 1 missed cleavage; 10 ppm peptide tolerance; peptide charges of 2+, 3+, or 4+; oxidation allowed as variable modification; 0.8 Da MS/MS tolerance and a MOWSE score > 13 to identify the corresponding protein. Mascot results were exported as .csv files. To match aligned *m*/*z* values from MALDI-MSI (Supplementary Table S1) with the peptides identified by nanoLC-MS/MS (Supplementary Table S2), we developed an excel macro in-house. The macro was applied with settings accommodating previously described parameters [37]. Briefly, the comparison of MALDI-MSI and LC−MS/MS m/z values required the identification of >1 peptide (mass differences < 0.9 Da). The peptides with highest MOWSE peptide score, smallest mass differences between MALDI-MSI and LC-MS/MS data and a correlation coefficient >0.1 or <0.1 were accepted as correctly identified.

### 2.4. MALDI-MSI Data Processing for Statistical Analyses

MALDI-MSI raw data were imported into the SCiLS Lab software version 2019c Pro (Bruker Daltonik GmbH) using settings preserving total ion count without baseline removal and converted into the SCiLS base data .sbd file and .slx file. An attribute table was built for the sample number and the different aortic wall layers tunica media and tunica adventitia. Attributes were used to divide the data set into independent data sets from different spatial spectral regions in tissue sections. Peak finding and alignment were conducted across a data set (interval width = 0.3 Da) using a standard segmentation pipeline (SciLS Lab software) in maximal interval processing mode with TIC normalization, medium noise reduction and no smoothing (Sigma: 0.75) [38,39].

### 2.5. Statistical Data Analysis

The top-down segmentation using bisecting k-means clustering analysis was performed on the partitioned data sets from tissue sections, as previously described [40], to defined proteomic signatures. Both analyses used settings for 0.3 Da interval width, include all individual spectra, and medium noise reduction and correlation distance. Discriminative MALDI-MSI *m*/*z* values were identified using supervised ROC analysis on the partitioned data sets from different tissue regions such as tunica media and tunica adventitia. The area under the ROC curve (AUC) varies between 0 and 1, where values close to 0 and 1 indicate peptides to be discriminatory and 0.5 indicates no discriminatory value. Since the number of *m*/*z* values from comparison groups must be similar for analysis, 35,000 *m*/*z* values were randomly selected per group. For those peptides with an AUC > 0.6 or <0.4, a univariate hypothesis test (Wilcoxon rank sum test) was used to test the statistical significance of *m*/*z* values. Peptides with *p*-value < 0.001 and a peak correlation ratio > 0.5 were selected as candidate markers.

## 3. Results

### 3.1. Clinical Characterization

General patient characteristics are shown in Table 1. All patients were non-diabetic men. The mean overall age was 67 ± 10 years. No significant difference in subgroup comparison was observed for age (TAA = 59 ± 10 years vs. AAA = 73 ± 6 years vs. EVAR = 70 ± 7 years, *p* = 0.077). The mean aortic diameter was 69 ± 16 mm. Patients with AAA (AAA = 80 ± 19 mm vs. TAA = 54 ± 5 mm, *p* = 0.01) or EVAR (EVAR = 72 ± 12 mm vs. TAA = 54 ± 5 mm, *p* = 0.02) had a significantly larger aortic diameter compared to those with TAA.

Indications for open aortic repair included rapid aneurysm growth (>5 mm in six months or >10 mm in one year, n = 2) or exceeded a threshold diameter of 55 mm (n = 8) for TAA and AAA patients. Patients formerly treated by EVAR developed several types of EL requiring open surgery for EL type Ia (n = 1), EL type II (n = 2), EL type III (n = 1), and EL type V (n = 1).

### 3.2. MALDI-MSI Data

**Figure 1:**
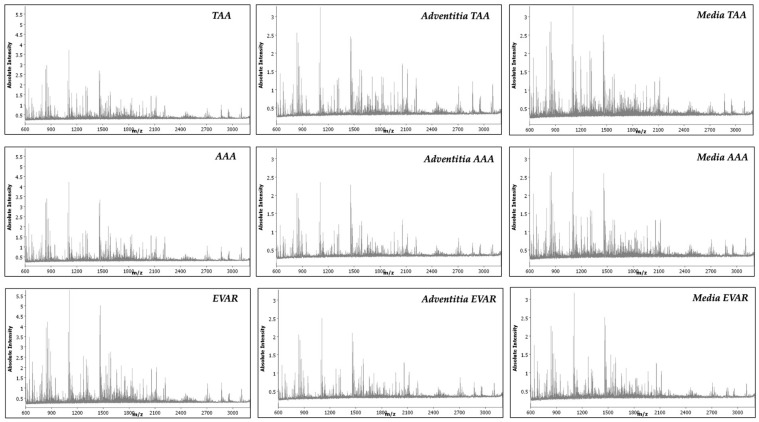
MALDI-MSI average spectra from the whole tissue sections and annotated regions of interest of tunica adventitia and tunica media in TAA, AAA, and EVAR tissue samples.

Primary proteomic screenings were performed simultaneously for TAA, AAA, and EVAR tissue sections. Subsequently, mass spectra for the annotated regions of interest (ROI, tunica media, and tunica adventitia) were obtained and statistically analyzed using SCiLS Lab software (Bruker, Bremen, Germany). Analysis of the whole tissue sections revealed 46.446, 75.128 and 126.259 spectra for TAA, AAA, and EVAR specimens, respectively. Annotated ROIs in TAA, AAA, and EVAR revealed 10.014, 11.988, and 27.112 spectra for tunica adventitia and 22.661, 8932, and 16.588 spectra for tunica media, respectively. Average exemplary spectra are shown for TAA, AAA, and EVAR subregions in Figure 1. The peptide signatures extracted from the analyzed tissue samples yielded 476 aligned peptide values (Supplementary Table S1) in a mass range for tryptic peptides (*m*/*z* value range: 600–3200).

### 3.3. Discriminative Proteins from TAA, AAA, and EVAR Tissue Sections Based on MALDI-MSI Data

To provide a better understanding of the pathophysiological differences in aortic wall layers, specific localized peptide values were explored in TAA, AAA, and EVAR tissue sections. Based on the typical wall structure of the aorta, sections of tunica media, and tunica adventitia were defined for analysis.

The identification of peptide values might provide insights into aneurysm formation and alterations of aortic wall structure after EVAR. To identify the corresponding proteins to the discriminatory tryptic peptide fragments, we performed a bottom-up LC-MS/MS approach in adjacent tissue sections. In total, 476 peptide values were detected via MALDI-MSI in annotated ROIs. Of those, 284 peptide values could be assigned to peptide values derived from LC-MS/MS (mass differences < 1 Da) corresponding to 91 proteins (Supplementary Table S2). After comparison of MALDI-MSI and LC−MS/MS (Supplementary Table S3) peptide values (requiring identification of >1 peptide to one protein), 84 peptide values revealed 28 corresponding proteins. ROC analysis was used on the total 284 aligned peptide peaks from annotated tunica adventitia (Supplementary Table S4) and tunica media (Supplementary Table S5) in AAA, TAA, and EVAR tissue sections. Overall, analysis for tunica adventitia revealed 82 and 62 discriminative peptide values for comparison of AAA and TAA and for EVAR and TAA, respectively. For tunica media, 63, 52, and 257 discriminative peptide values have been identified for subgroup comparison (AAA vs. EVAR, AAA vs. TAA, and EVAR vs. TAA). As the formation of an aortic aneurysm is largely caused by changes within the tunica media, we focus our interpretation of the results only on the peptide alterations observed in this area.

**Figure 2:**
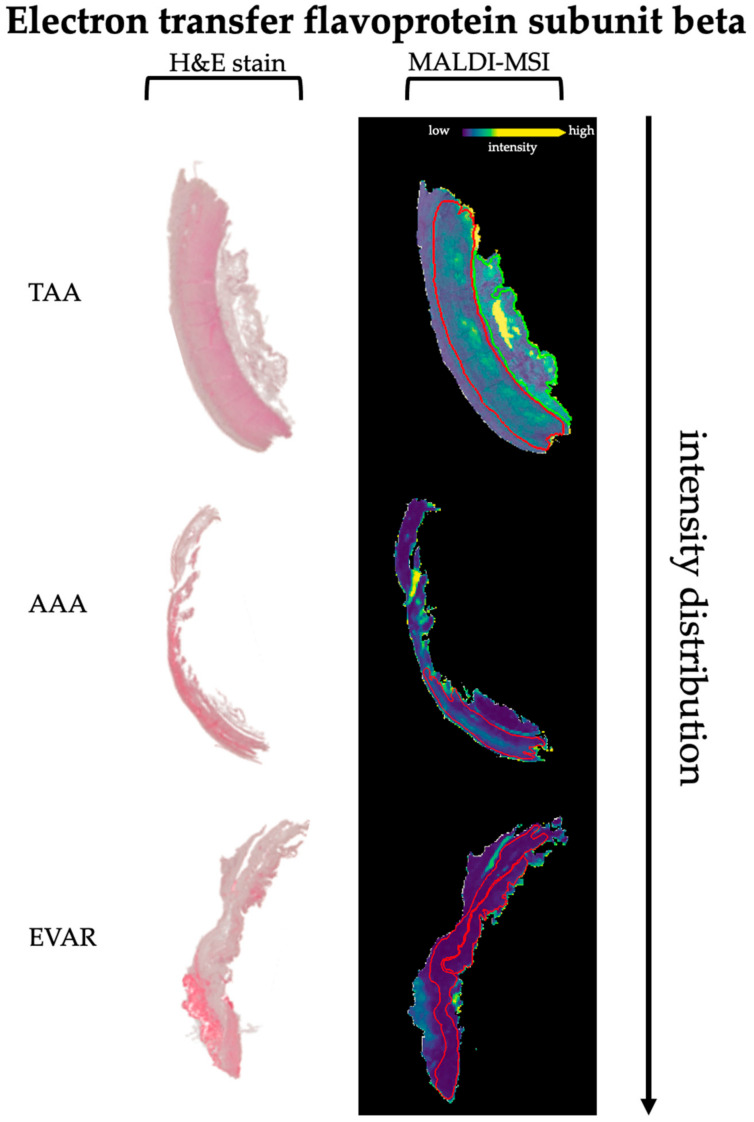
Differential intensity distributions of electron transfer flavoprotein subunit beta between TAA, AAA, and EVAR specimens.

**Figure 3:**
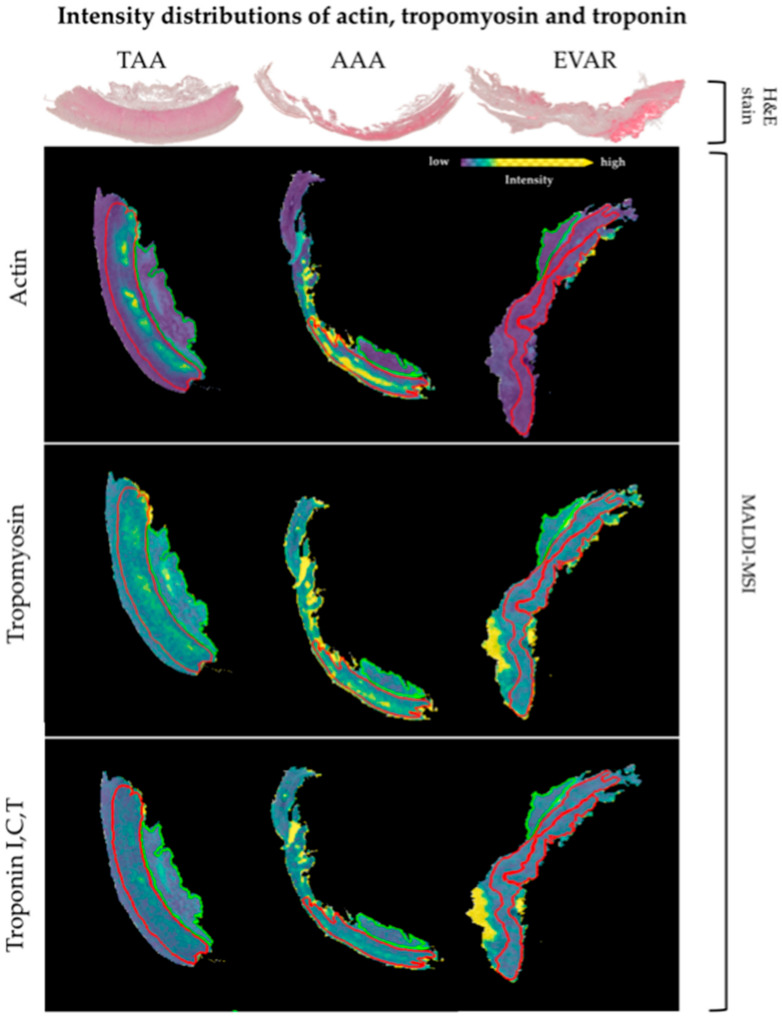
Differential intensity distributions of actin, tropomyosin, and troponin between TAA, AAA, and EVAR specimens.

Corresponding proteins to peptide values are correctly identified when the validating approach identifies at least two peptide values (detected in MALDI-MSI) from the same protein (AUC < 0.4, >0.6, *p* < 0.001) [36]. This revealed 47 peptide values with 17 corresponding proteins (ACTA, CAD13, CO1A1, CO1A2, CO6A3, KCRM, DESM, ETFB, H13, H4, MYH6, PGAM2, TPM1, TNNI3, TNNT2, TBB5, VME). In comparison to EVAR, nine peptide values with their corresponding four proteins (ACTA, ETFB, TPM1, TBB5) were increased in AAA tissue sections. Moreover, 46 *m*/*z* values from 17 corresponding proteins (ACTA, CAD13, CO1A1, CO1A2, CO6A3, KCRM, DESM, ETFB, H13, H4, MYH6, PGAM2, TPM1, TNNI3, TNNT2, TBB5, VME) are decreased in EVAR specimens compared to TAA tissue sections. The peptide values corresponding to ACTA were decreased in AAA specimens compared to TAA specimens (Table 2). As an example, we show intensity distributions in separate tissue sections for ETFB in Figure 2 and Supplementary Figure S1. Moreover, illustrative intensity distributions for actin, tropomyosin, and troponin are shown in Figure 3. The remaining peptides and their corresponding protein intensity distributions are shown in Supplementary Figure S2.

The relative peptide expression (color bar) is shown for MALDI m/z ion peaks with the highest significant area under the curve (AUC) values (>0.6, *p* < 0.001, on top) in receiver operator characteristic (ROC) analysis and the lowest AUC values (<0.4, *p* < 0.001, bottom MALDI images). Red lines represent tunica media. Hematoxylin and eosin (H&E) staining in sections is shown for orientation.

Relative peptide expression (color bar) is shown for MALDI m/z ion peaks with the highest significant area under the curve (AUC) values (>0.6, *p* < 0.001, left) in receiver operator characteristic (ROC) analysis and the lowest AUC values (<0.4, *p* < 0.001, right MALDI images). Red lines represent tunica media and green lines represent tunica adventitia. Hematoxylin and eosin (H&E) staining in sections is shown for orientation.

## 4. Discussion

### 4.1. Summary

As a unique mass spectrometric technique which combines spatial molecular analysis and histological assessment, MALDI-MSI identified proteomic signatures in aortic walls in TAA, AAA, and after, EVAR. Since little is known about pathophysiological changes after EVAR, the advantage of MALDI-MSI is seen in the absence of requirements for labelling or knowledge of molecular targets to analyze the distribution of hundreds of peptides. Furthermore, preservation of the spatial coordinates allows the linkage of alterations in protein distributions to specific regions inside the aortic wall layers. Thus, MALDI-MSI as a new technique seems to be an optimal tool to gain new insights on underlying changes inside the aortic wall. 

Acquired spatial proteomic signatures revealed 17 proteins with altered distributions among the tissue sections in EVAR and TAA or AAA, respectively. Actin, tropomyosin, and troponin, associated with the contractile unit in VSMC, showed decreased intensity distribution after EVAR. Furthermore, a decrease in extracellular matrix (collagen) or cytoskeletal proteins (desmin, tubulin) and electron carrier protein (electron transferring flavoprotein) could be observed in aortic wall specimens after EVAR. Based on these results, three spatial alterations could contribute to impaired wall stability after EVAR: (1) loss of function of the contractile unit in VSMC, (2) continuous degradation of extracellular matrix (ECM) proteins, and (3) reduced capacity to provide energy.

### 4.2. Impaired Vascular Smooth Muscle Cell Contractility after EVAR

Although the exact pathophysiological mechanism in aortic aneurysm formation remains unclear, VSCM are considered to play a central role depending on their phenotype [15]. Degradation of ECM induced by increased production of elastolytic enzymes, such as matrix metalloproteinases (MMP) by synthetic VSMC, is assumed to be one of the main reasons for aneurysm growth and rupture [3,4]. However, most VSMC in the aortic wall display a contractile phenotype responsible for regulating vascular tone. 

To maintain contractile function, VSMCs express α-smooth muscle actin (aSMA), which forms the thin filament of the contractile unit with tropomyosin (Tm), calmodulin, and caldesmon [41]. Although it has been demonstrated that mutations affecting the contractile unit, such as ACTA2 and MYH11-gene mutations [17,18], contribute to the formation of thoracic aneurysms or dissections, still, little is known about the loss of VSMC contractile function in the pathogenesis of aortic aneurysm. 

Recently, Bogunovic et al. [19] reported about the contractility in VSMC isolated from controls and sporadic AAA in human specimens by using electric cell-substrate impedance sensing (ECIS). No significant overall difference between AAA-patients and the control group in mean and maximum contraction was observed. Therefore, patients were subsequently divided into low and high contracting groups (defined as lower or higher than two standard deviations of results in the control group). Nevertheless, based on the findings that 28% (6/21) and 23% (5/21) of AAA patients showed lower contractility and low maximum contraction, respectively, the authors hypothesized that an impaired contractile function in VSMC in AAA patients might play a pathophysiological role in aneurysm formation. Furthermore, four patients formerly treated by EVAR were analyzed. Interestingly, VSMC obtained from those patients demonstrated a nearly significant (*p* = 0.05) trend of lower maximum contraction compared to the control group. Our data showed a lower intensity distribution of aSMA in the EVAR tissue sections compared to AAA and TAA specimens, respectively. In particular, this might explain the trend of impaired VSMC contraction in EVAR patients already observed by Bogunovic et al. 

However, the functionality of the contractile unit is complemented by other modulating proteins such as Tm and troponin. Although, Tm had neither been reported in AAA formation nor in EVAR complications, its importance to maintain contractile function as a calcium sensor is well established [41]. Historical assumptions that Tn proteins are not expressed in VSCM have been shown to be incorrect [42,43]. The exact role of Tn during the process of contraction in smooth muscle cells, particularly in VSMC, remains unclear. Kajioka et al. [43] not only provided evidence of the presence of all subunits of Tn in smooth muscle cells outside of cardiac muscle (e.g., aortic VSMC, trachea, urinary bladder) but also demonstrated its role in smooth muscle cell contraction. Their results suggest that particularly the complex of tropomyosin and TnT make a substantial contribution to smooth muscle contraction. With the detection of TnT and TnI, we confirm the expression of Tn outside the cardiac muscle. Furthermore, we see a significantly lower level of Tm, TnT, and TnI within the aortic wall after EVAR supporting the thesis of reduced VSMC contractility after stentgraft implantation.

In conclusion and based on our results of only a small-sample-sized patient cohort, the interaction of aSMA, myosin, Tm, and Tn assumably plays a central role in smooth muscle cell contraction. Our data provide evidence that, particularly after EVAR, a significant reduction of proteins elementarily important for VSMC contraction (aSMA, Tm, Tn) can be observed. If the aneurysm sac is successfully eliminated after EVAR, there is no risk of secondary rupture due to decreased contractility of the VSMC. However, if an EL occurs, the loss of the ability to respond to pulsatile blood flow by contraction may possibly promote rupture of the aortic wall.

### 4.3. Increased ECM Degradation after EVAR

In addition to the VSMCs, ECM is essential to maintain this physiological function. Within the three-layered structure of the aortic walls, a variety of ECM proteins compose a three-dimensional organization [16]. 

Collagen and elastic fibers comprise approximately 50% of the dry weight of larger arteries and function as the main proteins providing tensile strength and expandability [44]. Additionally, they play a key role in modulating the adhesion, proliferation, and migration of VSMC by interacting with various integrins and proteins [45]. ECM degradation in tunica media and tunica adventitia has proven to be involved in aneurysm formation [3,4].

Collagen, as the main structural protein in the aortic wall, mainly consists of fibrillar collagens type I and III, accounting for 80–90% of the total collagen. Both increased collagen I/III levels enhancing arterial stiffness and decreased collagen I/III levels weakening the aortic wall have been proven to favor aortic aneurysm and dissection [46,47]. Augmented expression of MMPs is well documented in human and mouse aortic aneurysm [48] leading to degradation of aortic ECM and collagen cleavage in particular. In contrast, Menges et al. [14] revealed an altered collagen composition in AAA and EVAR patients compared to healthy aorta with a high abundance of collagen I and decreased expression of collagen III, respectively. Underlining the importance of collagen integrity, Lee et al. [49] associated the use of fluoroquinolones to collagen degradation and higher risk of aortic aneurysm formation aortic dissection. Particularly with a significant decrease in collagen types I and III seen after EVAR, our data might provide further evidence that collagen is continued to be cleaved and degraded after stentgraft implantation. Based on this assumption, this may result in progression of aneurysm formation and lowers the tensile strength of the remaining aortic wall in case of reinstating pulsatile blood flow in case of EL.

As previously mentioned, depending on the phenotype, the increased expression of elastolytic enzymes by synthetic VSMC results in ECM degradation. The shift of the VSMC phenotype from actin- and desmin-expressing contractile phenotype to synthetic phenotype weakens the aortic wall [50,51]. Desmin, actin, and tubulin [52] can be used to define contractile phenotype in VSMC. In comparison to non-AAA samples, AAA samples contain significantly less actin and desmin, suggesting higher levels of synthetic VSMC in AAA patients [47]. 

Bogunovic et al. [19] examined if the observed decreased contractility might depend on the phenotype defect of VSMC. Therefore, changes in VSMC-specific marker genes (ACTA2, CNN1, TAGLN) and protein expression (aSMA, Calponin, SM22) of VSMC marker proteins were studied using quantitative PCR and Western blot, respectively. Again, the heterogeneous results in the small-sized comparison groups prevented a significant result. We observed a lower expression of desmin and tubulin after EVAR, which could be a potential sign of a lower level of contractile VSMCs. Aforementioned, we already assume to provide evidence for reduced VSMC contractility due to decreased expression of proteins necessary for contractile function. In addition, the lower expression of desmin and tubulin might support the idea that not only reduced expression of contractile proteins but also a lower proportion of contractile VSMCs in the aortic wall after EVAR might be able to reduce resistance of the aortic wall in case of pulsatile blood flow. Thus, ECM degradation and reduced VSMC contractility might benefit the risk of rupture due to aortic wall destabilization. 

### 4.4. Alteration of the Energy Supply after EVAR

The transport of electrons to the membrane-bound respiratory chain also involves electron transferring falvoproteins (ETFs) [53]. ETFs are soluble heterodimeric FAD-containing proteins [54] and function as electron carriers between various flavoprotein-containing dehydrogenases. At least nine mitochondrial matrix flavoprotein dehydrogenases (acyl-CoA dehydrogenase, isovaleryl-CoA dehydrogenase, 2-methyl branched-chain acyl-CoA dehydrogenase, glutaryl-CoA dehydrogenase, dimethyl-glycine and sarcosine dehydrogenases) donor electrons to ETFs. Thus, ETFs are indirectly involved in several energetic pathways (fatty acid β-oxidation, amino acid oxidation, choline metabolism [55,56]. Electrons are transported by ETFB to the membrane-bound ETF-ubiquinone oxidoreductase [57], which results in a reduction of ubiquinone to ubiquinol.

No studies are available regarding impaired mitochondrial function or respiratory chain disorders in TAA and/or AAA formation. Nevertheless, our data might provide evidence for a reduced or nearly eliminated capacity of the ETF in AAA and EVAR patients. Particularly after implantation of an endovascular stentgraft, restricted nutrition might be the reason for reduced energy production. Under normal conditions, cells of the aortic wall are largely dependent on intimal diffusion [58]. Not only ending endoluminal diffusion, the endovascular implantation of the stentgraft may restrict the collateral supply of nutrition brought by the vasa vasorum. However, the significantly lower concentration of ETF in AAA than in TAA may additionally suggest a relevant role in the formation of AAA through reduced energy supply. Since ATP as a universally usable energy carrier is indispensable for triggering a muscle contraction and thus also the contraction of the VSMCs; additionally, the circle might be closed to the assumption of a limited functional capacity of the contractile unit.

## 5. Conclusions

In summary, our data successfully demonstrate the feasibility of using MALDI-MSI to discriminate aortic wall specimens in patients with aneurysm disease and after the implantation of an endovascular stentgraft. Not only by detecting various alterations of protein distributions in general but also by directly assigning them to specific wall layers, MALDI-MSI is ideally suited for detecting ongoing remodeling processes after EVAR. The advantage of preserving the spatial coordinates could be used to link remodeling alterations to specific regions, even to regions inside the single aortic layers, and might allow conclusions about location-related changes in specific regions of interest.

The collected data might support the hypothesis of impaired contractility of VSMCs in the aortic wall after EVAR due to reduced synthesis of aSMA, Tm, and Tn, proteins that are elementally important for contraction. In addition, a reduced supply of the energy carrier ATP could promote decreased contractility through reduced electron transport to the respiratory chain. In combination with a continuous degradation of ECM proteins, this might result in a weakened aortic wall not able to react on reinstating pulsatile blood flow in case of EL after EVAR.

Nevertheless, the findings should only be interpreted in the context of a small sample size. Additionally, comparison to non-AAA samples and further analysis with methods such as immunohistochemistry could validate the results and might help to support the hypothesis. However, if the results can be confirmed in further studies, MALDI-MSI appears to be an excellent method to detect initial remodeling processes leading to complications after EVAR and could be an important tool for initiating subsequent preventive therapy concepts.

## Figures and Tables

**Table 1:** Indications and patient characteristics.

Subgroup	Gender	Age	Maximum Aneurysm Diameter (mm)	Comorbidities
TAA_1	M	62	58	CAD, HI
TAA_2	M	67	59	HI
TAA_3	M	54	52	HI
TAA_4	M	43	57	HI
TAA_5	M	68	46	CAD, HI, AHT, HLP, COPD, CRF
AAA_1	M	78	60	CAD, AHT, HLP
AAA_2	M	72	81	CRF, AHT
AAA_3	M	78	80	CAD, HLP
AAA_4	M	63	110	AHT
AAA_5	M	76	68	CAD, HI, AHT
EVAR_1	M	67	75	CAD, PAD, AHT
EVAR_2	M	64	90	CAD, AHT, HLP
EVAR_3	M	80	65	AF, PAD
EVAR_4	M	73	59	CAD, CRF
EVAR_5	M	65	69	AHT, PAD

CAD = coronary artery disease, HI = heart insufficiency, AHT = arterial hypertension, HLP = hyperlipidemia, COPD = chronic obstructive pulmonary disease, CRF = chronic renal failure, PAD = peripheral artery disease, AF = atrial fibrillation.

**Table 2:** Differential peptides (MALDI-MSI) and their corresponding proteins from the tunica media in TAA, AAA, and EVAR tissue sections.

MALDI-MSI *m*/*z* Value [Da]	ROC [AUC] Media AAA vs. EVAR	ROC [AUC] Media AAA vs. TAA	ROC [AUC] Media EVAR vs. TAA	LC-MS [MH + Calc.]	Scores	Sequence	Gene Symbol	Protein
976.43	0.62	0.35	0.22	976.4468194	88.56	AGFAGDDAPR	ACTA2	Actin, aortic smooth muscle
1198.65	0.66	0.29	0.10	1198.51941	57.19	DSYVGDEAQSK		
1198.65	0.66	0.29	0.10	1198.703002	41.77	AVFPSIVGRPR		
1580.684	0.56	0.45	0.39	1580.800411	43.04	MQKEITALAPSTMK		
1790.904	0.57	0.36	0.27	1790.891339	28.06	SYELPDGQVITIGNER		
1564.883	0.59	0.42	0.33	1564.905355	31.49	SIVVSPILIPENQR	CDH13	Cadherin-13
1835.908	0.58	0.44	0.34	1836.842546	32.84	MTAFDADDPATDNALLR		
836.417	0.59	0.42	0.31	836.4361428	49.08	GPAGPQGPR	COL1A1	Collagen alpha-1(I) chain
852.418	0.61	0.40	0.28	851.4249814	43.88	GFSGLDGAK		
868.42	0.59	0.42	0.31	868.4253882	34.63	GEAGPQGPR		
886.421	0.56	0.43	0.36	886.4359955	47.39	GSEGPQGVR		
784.412	0.58	0.45	0.37	785.3889089	48.12	GDQGPVGR	COL1A2	Collagen alpha-2(I) chain
1561.883	0.60	0.48	0.37	1562.79067	87.57	GETGPSGPVGPAGAVGPR		
1237.653	0.57	0.43	0.37	1238.651334	47.18	VAVFFSNTPTR	COL6A3	Collagen alpha-3(VI) chain
1459.673	0.61	0.43	0.32	1459.860756	25.39	IGDLHPQIVNLLK		
1462.674	0.55	0.44	0.39	1462.763096	27.26	QINVGNALEYVSR		
1508.678	0.60	0.40	0.32	1507.799786	83.26	LSVEALNSLTGEFK	CKM	Creatine kinase M-type Desmin
1508.678	0.60	0.40	0.32	1507.699725	58.36	GGDDLDPNYVLSSR		
1767.902	0.57	0.47	0.39	1768.83488	32.75	DGEVVSEATQQQHEVL		
2088.931	0.56	0.45	0.39	2088.091512	51.57	TFGGAPGFPLGSPLSSPVFPR		
853.418	0.61	0.40	0.27	853.5233919	55.31	LGPLQVAR	ETFB	Electron transfer flavoprotein subunit beta
1340.663	0.60	0.44	0.34	1339.720602	51.07	LSVISVEDPPQR		
974.429	0.56	0.43	0.38	973.6021152	45.36	SGVSLAALKK	H1-3	Histone H1.3
1106.641	0.58	0.42	0.32	1107.565851	47.87	ALAAAGYDVEK		
1198.65	0.66	0.29	0.10	1198.666651	64.14	ASGPPVSELITK		
1325.661	0.59	0.44	0.36	1325.752447	45.61	DNIQGITKPAIR	H4C1	Histone H4
1466.674	0.60	0.42	0.32	1466.801839	61.03	TVTAMDVVYALKR		
1533.68	0.58	0.44	0.34	1533.775171	82.04	VVDSLQTSLDAETR	MYO6	Myosin-6
1850.909	0.59	0.44	0.34	1851.041427	48.84	VQLLHSQNTSLINQKK		
2088.931	0.56	0.45	0.39	2088.123001	33.09	YRILNPVAIPEGQFIDSR		
2199.941	0.57	0.46	0.39	2200.123705	49.68	GTLEDQIIQANPALEAFGNAK		
976.43	0.62	0.35	0.22	975.4887038	36.63	AMEAVAAQGK	PGAM2	Phosphoglycerate mutase 2
1150.645	0.56	0.43	0.38	1150.666958	45.55	VLIAAHGNSLR		
875.42	0.57	0.44	0.37	875.4465169	30.27	SLEAQAEK	TPM1	Tropomyosin alpha-1 chain
1460.674	0.62	0.43	0.31	1460.731208	39.4	KATDAEADVASLNR		
1516.679	0.61	0.43	0.32	1516.819568	27.34	SKQLEDELVSLQK		
1305.659	0.58	0.41	0.34	1306.638768	44.38	KNIDALSGMEGR	TNNI3	Troponin I, cardiac muscle
1479.675	0.54	0.43	0.40	1479.727686	42.27	ISADAMMQALLGAR		
1889.913	0.57	0.46	0.38	1890.031221	44.46	NITEIADLTQKIFDLR		
758.41	0.55	0.45	0.40	757.4673223	35.39	ILAERR	TNNT2	Troponin T, cardiac muscle
906.423	0.55	0.43	0.37	906.5021046	26.65	YEINVLR		
1797.904	0.57	0.46	0.40	1796.934971	29.11	SFMPNLVPPKIPDGER		
1143.445	0.60	0.42	0.31	1143.632775	28.94	LAVNMVPFPR	TUBB	Tubulin beta chain
1320.661	0.64	0.38	0.24	1319.701066	49.82	IMNTFSVVPSPK		
1269.656	0.56	0.43	0.37	1270.559399	37.39	LGDLYEEEMR	VIM	Vimentin
1428.671	0.55	0.33	0.28	1428.710851	40.41	SLYASSPGGVYATR		
2498.168	0.55	0.49	0.44	2497.256473	42.86	LLQDSVDFSLADAINTEFKNTR		

## Data Availability

Data are contained within the article or supplementary material. The MALDI-MSI data presented in this study are available on request from the corresponding author.

## Supplementary Materials

The following are available online at https://www.mdpi.com/article/10.3390/biomedicines9091088/s1, Figure S1: Differential intensity distributions of electron transfer flavoprotein subunit beta in all specimens Relative peptide expression (color bar) is shown for MALDI m/z ion peaks. Red lines represents tunica media. Figure S2: Differential intensity distributions of vimentin, tubulin, collagen alpha-1 (I) chain and desmin in TAA, AAA and EVAR specimens Relative peptide expression (color bar) is shown for MALDI m/z ion peaks. Red lines represents tunica media and green lines represent tunica adventitia. Hematoxylin and eosin (H&E) staining in sections is shown for orientation. Table S1: Overall differential intensity distributions of m/z values in TAA, AAA and EVAR specimens. Table S2: Peptides identified by nanoLC-MS/MS. Table S3: Differential intensity distributions of all peptides (MALDI-MSI) and their corresponding proteins from the tunica adventitia in TAA, AAA and EVAR tissue sections. Table S4: Differential intensity distributions of all peptides (MALDI-MSI) and their corresponding proteins from the tunica media in TAA, AAA and EVAR tissue sections. Table S5: Differential intensity distributions of all peptides (MALDI-MSI) and their corresponding proteins from the tunica media in TAA, AAA and EVAR tissue sections.
