# Supplementary material for: Use of MALDI Mass Spectrometry Imaging to Identify Proteomic Signatures in Aortic Aneurysms after Endovascular Repair
Source: Biomedicines. 2021 Aug 26;9(9):1088. doi: 10.3390/biomedicines9091088 (PMC8465851; doi:10.3390/biomedicines9091088)
Supplement: Supplementary file 1 [file biomedicines-09-01088-s001.zip › biomedicines-1294606-supplementary.pdf]

Supplementary Figure S1. Differential intensity distributions of electron transfer flavoprotein subunit beta in all specimens Relative peptide expression (color bar) is shown for MALDI m/z ion peaks. Red lines represents tunica media.

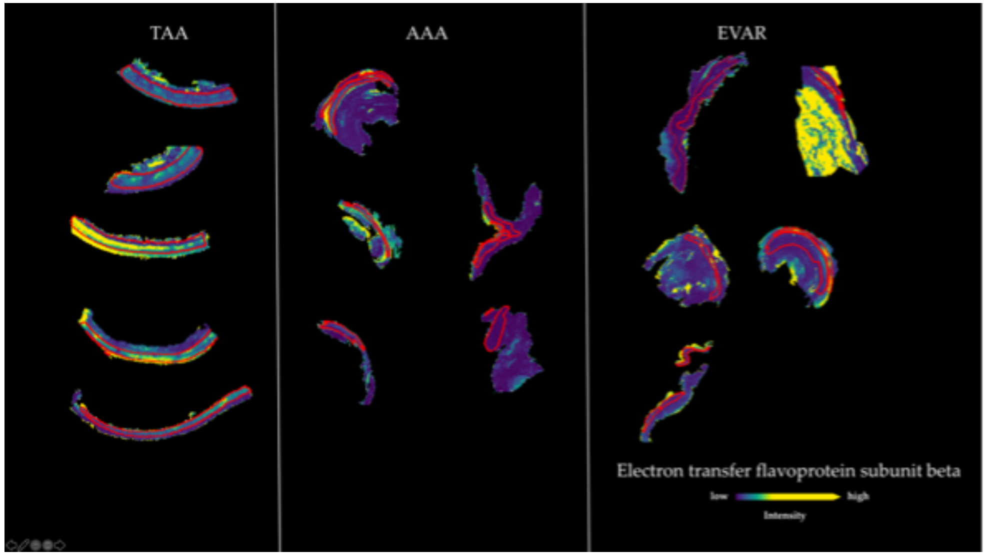

Supplementary Figure S2. Differential intensity distributions of vimentin, tubulin, collagen alpha-1 (I) chain and desmin in TAA, AAA and EVAR specimens Relative peptide expression (color bar) is shown for MALDI m/z ion peaks. Red lines represents tunica media and green lines represent tunica adventitia. Hematoxylin and eosin (H&E) staining in sections is shown for orientation.

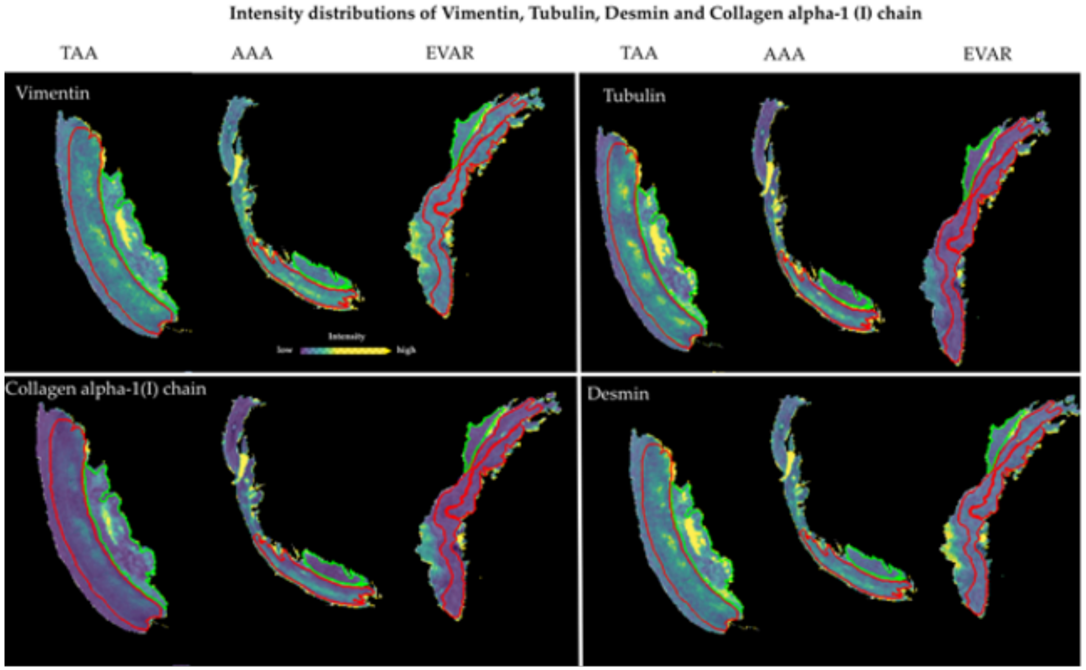

Supplementary Table S1. Overall differential intensity distributions of m/z values in TAA, AAA and EVAR specimens

MALDI-MSI value [Da]	ROC [AUC] Adventitia AAA vs EVAR	ROC [AUC] Adventitia AAA vs TAA	ROC [AUC] Adventitia EVAR vs EVAR vs TAA	ROC [AUC] Media AAA vs EVAR	ROC [AUC] Media AAA vs TAA	ROC [AUC] Media EVAR vs EVAR vs TAA
602.396 m/z ± 0.154 Da	0,47	0,41	0,43	0,63	0,40	0,26
603.396 m/z ± 0.154 Da	0,49	0,44	0,44	0,59	0,42	0,31
616.197 m/z ± 0.154 Da	0,54	0,61	0,55	0,51	0,57	0,56
617.197 m/z ± 0.154 Da	0,53	0,59	0,56	0,50	0,55	0,54
618.397 m/z ± 0.154 Da	0,49	0,40	0,37	0,61	0,40	0,29
620.397 m/z ± 0.154 Da	0,50	0,40	0,40	0,62	0,39	0,25
628.398 m/z ± 0.154 Da	0,47	0,43	0,44	0,60	0,40	0,29
643.999 m/z ± 0.154 Da	0,53	0,54	0,52	0,54	0,49	0,43
644.399 m/z ± 0.154 Da	0,49	0,41	0,41	0,58	0,42	0,33
644.999 m/z ± 0.154 Da	0,54	0,56	0,51	0,54	0,49	0,42
645.399 m/z ± 0.154 Da	0,49	0,42	0,41	0,57	0,42	0,34
650 m/z ±	0,51	0,49	0,46	0,55	0,49	0,43

0.154 Da						
651 m/z ± 0.154 Da	0,52	0,50	0,49	0,54	0,50	0,46
666.001 m/z ± 0.154 Da	0,53	0,53	0,49	0,52	0,52	0,50
678.402 m/z ± 0.154 Da	0,44	0,37	0,41	0,57	0,43	0,34
679.403 m/z ± 0.154 Da	0,46	0,40	0,41	0,55	0,46	0,38
683.403 m/z ± 0.154 Da	0,53	0,57	0,54	0,55	0,51	0,45
688.403 m/z ± 0.154 Da	0,51	0,51	0,48	0,61	0,44	0,33
700.404 m/z ± 0.154 Da	0,51	0,47	0,46	0,55	0,44	0,39
701.405 m/z ± 0.154 Da	0,49	0,47	0,48	0,55	0,38	0,33
730.407 m/z ± 0.154 Da	0,50	0,43	0,42	0,58	0,40	0,32
758.41 m/z ± 0.154 Da	0,50	0,45	0,46	0,55	0,45	0,40
759.41 m/z ± 0.154 Da	0,46	0,45	0,50	0,52	0,48	0,46
771.411 m/z ± 0.154 Da	0,52	0,44	0,41	0,57	0,45	0,36
784.412 m/z ±	0,51	0,46	0,45	0,58	0,45	0,37

0.154 Da					
785.412 m/z ± 0.154 Da	0,50	0,44	0,42	0,57	0,43	0,35
786.412 m/z ± 0.154 Da	0,50	0,45	0,44	0,55	0,44	0,38
795.413 m/z ± 0.154 Da	0,49	0,43	0,42	0,63	0,35	0,21
796.413 m/z ± 0.154 Da	0,48	0,43	0,44	0,62	0,39	0,27
815.415 m/z ± 0.154 Da	0,50	0,45	0,44	0,54	0,47	0,43
816.415 m/z ± 0.154 Da	0,45	0,46	0,51	0,53	0,46	0,42
817.415 m/z ± 0.154 Da	0,46	0,46	0,50	0,54	0,45	0,40
822.416 m/z ± 0.154 Da	0,49	0,51	0,50	0,52	0,47	0,45
823.416 m/z ± 0.154 Da	0,50	0,50	0,50	0,54	0,46	0,43
833.016 m/z ± 0.154 Da	0,54	0,56	0,52	0,55	0,49	0,44
833.417 m/z ± 0.154 Da	0,48	0,49	0,51	0,59	0,40	0,31
836.417 m/z ± 0.154 Da	0,52	0,43	0,41	0,59	0,42	0,31
837.417 m/z ±	0,56	0,45	0,41	0,60	0,43	0,31

0.154 Da					
838.417 m/z ± 0.154 Da	0,50	0,44	0,44	0,57	0,45	0,38
839.017 m/z ± 0.154 Da	0,53	0,52	0,51	0,54	0,49	0,47
840.417 m/z ± 0.154 Da	0,47	0,45	0,48	0,59	0,48	0,38
841.417 m/z ± 0.154 Da	0,47	0,42	0,45	0,59	0,46	0,37
842.417 m/z ± 0.154 Da	0,52	0,45	0,46	0,55	0,42	0,39
843.417 m/z ± 0.154 Da	0,50	0,49	0,48	0,51	0,44	0,44
852.418 m/z ± 0.154 Da	0,54	0,45	0,42	0,61	0,40	0,28
853.418 m/z ± 0.154 Da	0,52	0,43	0,42	0,61	0,40	0,27
854.418 m/z ± 0.154 Da	0,51	0,43	0,41	0,60	0,42	0,32
855.018 m/z ± 0.154 Da	0,53	0,53	0,51	0,55	0,47	0,41
856.019 m/z ± 0.154 Da	0,52	0,53	0,51	0,56	0,47	0,41
857.019 m/z ± 0.154 Da	0,53	0,53	0,52	0,54	0,50	0,46
861.019 m/z ±	0,51	0,49	0,48	0,52	0,50	0,48

0.154 Da						
862.019 m/z ± 0.154 Da	0,51	0,50	0,50	0,51	0,51	0,50
868.42 m/z ± 0.154 Da	0,50	0,42	0,43	0,59	0,42	0,31
869.42 m/z ± 0.154 Da	0,50	0,43	0,44	0,57	0,43	0,36
870.42 m/z ± 0.154 Da	0,51	0,44	0,42	0,56	0,46	0,40
871.02 m/z ± 0.154 Da	0,54	0,56	0,53	0,51	0,51	0,51
872.42 m/z ± 0.154 Da	0,52	0,45	0,43	0,55	0,37	0,33
874.42 m/z ± 0.154 Da	0,48	0,43	0,45	0,59	0,42	0,32
875.42 m/z ± 0.154 Da	0,51	0,48	0,46	0,57	0,44	0,37
877.02 m/z ± 0.154 Da	0,53	0,53	0,51	0,51	0,52	0,51
886.421 m/z ± 0.154 Da	0,49	0,40	0,41	0,56	0,43	0,36
887.421 m/z ± 0.154 Da	0,49	0,40	0,41	0,55	0,45	0,38
890.422 m/z ± 0.154 Da	0,54	0,49	0,45	0,57	0,39	0,30
891.422 m/z ±	0,54	0,51	0,48	0,56	0,42	0,35

0.154 Da					
898.422 m/z ± 0.154 Da	0,54	0,51	0,46	0,55	0,46	0,41
899.423 m/z ± 0.154 Da	0,54	0,47	0,42	0,56	0,46	0,39
904.423 m/z ± 0.154 Da	0,50	0,53	0,54	0,46	0,47	0,52
906.423 m/z ± 0.154 Da	0,51	0,50	0,48	0,55	0,43	0,37
944.427 m/z ± 0.154 Da	0,53	0,51	0,48	0,57	0,45	0,37
945.427 m/z ± 0.154 Da	0,51	0,46	0,46	0,56	0,45	0,38
946.427 m/z ± 0.154 Da	0,47	0,44	0,47	0,56	0,49	0,43
964.428 m/z ± 0.154 Da	0,50	0,49	0,49	0,55	0,46	0,41
974.429 m/z ± 0.154 Da	0,49	0,46	0,44	0,56	0,43	0,38
976.43 m/z ± 0.154 Da	0,52	0,43	0,40	0,62	0,35	0,22
980.43 m/z ± 0.154 Da	0,49	0,52	0,52	0,44	0,49	0,55
981.43 m/z ± 0.154 Da	0,45	0,49	0,53	0,45	0,50	0,54
982.43 m/z ±	0,48	0,50	0,52	0,52	0,48	0,46

0.154 Da						
1032.635 m/z ± 0.154 Da	0,43	0,49	0,55	0,46	0,49	0,53
1033.635 m/z ± 0.154 Da	0,42	0,48	0,58	0,45	0,47	0,52
1034.635 m/z ± 0.154 Da	0,47	0,51	0,53	0,46	0,49	0,52
1035.435 m/z ± 0.154 Da	0,53	0,55	0,53	0,48	0,46	0,48
1044.036 m/z ± 0.154 Da	0,53	0,54	0,51	0,55	0,50	0,46
1045.636 m/z ± 0.154 Da	0,48	0,46	0,48	0,49	0,47	0,48
1060.037 m/z ± 0.154 Da	0,51	0,53	0,53	0,53	0,49	0,42
1061.037 m/z ± 0.154 Da	0,54	0,55	0,51	0,54	0,48	0,43
1062.037 m/z ± 0.154 Da	0,53	0,55	0,50	0,55	0,50	0,45
1066.038 m/z ± 0.154 Da	0,55	0,53	0,49	0,57	0,48	0,42
1067.038 m/z ± 0.154 Da	0,53	0,52	0,50	0,54	0,49	0,45
1082.039 m/z ± 0.154 Da	0,51	0,52	0,51	0,51	0,52	0,52
1094.64 m/z ±	0,52	0,40	0,37	0,63	0,37	0,23

0.154 Da					
1095.64 m/z ± 0.154 Da	0,45	0,38	0,42	0,58	0,39	0,31
1096.64 m/z ± 0.154 Da	0,51	0,43	0,40	0,59	0,42	0,32
1097.641 m/z ± 0.154 Da	0,50	0,44	0,43	0,55	0,45	0,40
1105.641 m/z ± 0.154 Da	0,47	0,40	0,41	0,60	0,42	0,31
1106.641 m/z ± 0.154 Da	0,41	0,36	0,42	0,58	0,42	0,32
1107.641 m/z ± 0.154 Da	0,50	0,40	0,40	0,61	0,44	0,31
1108.642 m/z ± 0.154 Da	0,51	0,44	0,43	0,56	0,45	0,38
1111.642 m/z ± 0.154 Da	0,48	0,42	0,43	0,61	0,36	0,26
1112.642 m/z ± 0.154 Da	0,50	0,43	0,42	0,62	0,39	0,26
1113.642 m/z ± 0.154 Da	0,44	0,43	0,50	0,55	0,42	0,37
1116.442 m/z ± 0.154 Da	0,54	0,45	0,41	0,56	0,45	0,40
1117.442 m/z ± 0.154 Da	0,53	0,47	0,44	0,54	0,44	0,40
1118.442 m/z ±	0,53	0,50	0,47	0,53	0,47	0,45

0.154 Da						
1127.643 m/z ± 0.154 Da	0,46	0,43	0,47	0,56	0,47	0,42
1133.444 m/z ± 0.154 Da	0,51	0,50	0,47	0,59	0,43	0,34
1134.444 m/z ± 0.154 Da	0,53	0,52	0,48	0,56	0,43	0,38
1138.644 m/z ± 0.154 Da	0,43	0,38	0,43	0,59	0,35	0,25
1139.644 m/z ± 0.154 Da	0,42	0,37	0,45	0,60	0,38	0,27
1140.644 m/z ± 0.154 Da	0,47	0,42	0,45	0,60	0,41	0,31
1143.445 m/z ± 0.154 Da	0,52	0,51	0,49	0,60	0,42	0,31
1144.445 m/z ± 0.154 Da	0,47	0,47	0,49	0,58	0,45	0,36
1149.645 m/z ± 0.154 Da	0,47	0,46	0,47	0,58	0,43	0,35
1150.645 m/z ± 0.154 Da	0,49	0,49	0,50	0,56	0,43	0,38
1154.646 m/z ± 0.154 Da	0,44	0,37	0,44	0,63	0,40	0,28
1155.446 m/z ± 0.154 Da	0,51	0,48	0,48	0,57	0,45	0,38
1176.448 m/z ±	0,53	0,55	0,50	0,58	0,39	0,30

0.154 Da					
1198.65 m/z ± 0.154 Da	0,49	0,38	0,39	0,66	0,29	0,10
1199.65 m/z ± 0.154 Da	0,51	0,42	0,41	0,65	0,30	0,12
1200.65 m/z ± 0.154 Da	0,45	0,42	0,46	0,61	0,35	0,22
1214.651 m/z ± 0.154 Da	0,47	0,44	0,47	0,54	0,46	0,42
1223.652 m/z ± 0.154 Da	0,46	0,45	0,48	0,53	0,47	0,45
1230.653 m/z ± 0.154 Da	0,44	0,45	0,49	0,53	0,46	0,43
1231.653 m/z ± 0.154 Da	0,52	0,50	0,47	0,58	0,44	0,35
1235.653 m/z ± 0.154 Da	0,46	0,38	0,41	0,57	0,44	0,36
1236.653 m/z ± 0.154 Da	0,49	0,40	0,40	0,57	0,42	0,34
1237.653 m/z ± 0.154 Da	0,52	0,45	0,41	0,57	0,43	0,37
1240.654 m/z ± 0.154 Da	0,50	0,57	0,56	0,52	0,51	0,49
1241.654 m/z ± 0.154 Da	0,51	0,55	0,53	0,52	0,45	0,43
1264.456 m/z ±	0,48	0,48	0,50	0,48	0,51	0,53

0.154 Da					
1267.656 m/z ± 0.154 Da	0,47	0,42	0,45	0,57	0,40	0,34
1268.656 m/z ± 0.154 Da	0,54	0,47	0,43	0,59	0,43	0,34
1269.656 m/z ± 0.154 Da	0,52	0,45	0,42	0,56	0,43	0,37
1271.056 m/z ± 0.154 Da	0,51	0,53	0,51	0,53	0,49	0,46
1274.657 m/z ± 0.154 Da	0,47	0,50	0,54	0,51	0,54	0,53
1275.657 m/z ± 0.154 Da	0,46	0,51	0,56	0,48	0,49	0,51
1297.659 m/z ± 0.154 Da	0,52	0,44	0,43	0,53	0,44	0,41
1302.659 m/z ± 0.154 Da	0,48	0,43	0,43	0,57	0,48	0,41
1303.659 m/z ± 0.154 Da	0,48	0,39	0,40	0,62	0,36	0,24
1304.659 m/z ± 0.154 Da	0,50	0,42	0,42	0,62	0,39	0,27
1305.659 m/z ± 0.154 Da	0,47	0,44	0,45	0,58	0,41	0,34
1314.66 m/z ± 0.154 Da	0,46	0,49	0,55	0,50	0,41	0,42
1315.66 m/z ±	0,48	0,50	0,53	0,51	0,42	0,42

0.154 Da						
1316.66 m/z ± 0.154 Da	0,46	0,50	0,53	0,51	0,45	0,44
1320.661 m/z ± 0.154 Da	0,57	0,48	0,41	0,64	0,38	0,24
1321.661 m/z ± 0.154 Da	0,55	0,47	0,42	0,63	0,37	0,24
1322.661 m/z ± 0.154 Da	0,53	0,46	0,45	0,60	0,38	0,26
1324.661 m/z ± 0.154 Da	0,54	0,48	0,43	0,59	0,43	0,34
1325.661 m/z ± 0.154 Da	0,45	0,45	0,48	0,59	0,44	0,36
1326.661 m/z ± 0.154 Da	0,44	0,46	0,53	0,54	0,46	0,41
1337.662 m/z ± 0.154 Da	0,48	0,40	0,45	0,57	0,39	0,33
1340.663 m/z ± 0.154 Da	0,50	0,46	0,46	0,60	0,44	0,34
1341.663 m/z ± 0.154 Da	0,46	0,44	0,46	0,58	0,42	0,34
1342.663 m/z ± 0.154 Da	0,48	0,45	0,47	0,57	0,43	0,35
1352.664 m/z ± 0.154 Da	0,50	0,48	0,47	0,52	0,44	0,43
1353.664 m/z ±	0,49	0,48	0,49	0,51	0,48	0,47

0.154 Da						
1358.664 m/z ± 0.154 Da	0,49	0,46	0,48	0,60	0,41	0,30
1359.664 m/z ± 0.154 Da	0,52	0,47	0,47	0,63	0,40	0,26
1366.665 m/z ± 0.154 Da	0,49	0,41	0,41	0,59	0,37	0,29
1390.667 m/z ± 0.154 Da	0,48	0,50	0,52	0,50	0,50	0,50
1391.667 m/z ± 0.154 Da	0,46	0,48	0,51	0,46	0,48	0,52
1401.668 m/z ± 0.154 Da	0,48	0,43	0,44	0,55	0,44	0,39
1408.669 m/z ± 0.154 Da	0,55	0,46	0,42	0,60	0,31	0,20
1409.669 m/z ± 0.154 Da	0,49	0,44	0,46	0,56	0,34	0,25
1428.671 m/z ± 0.154 Da	0,50	0,44	0,44	0,55	0,33	0,28
1459.673 m/z ± 0.154 Da	0,54	0,46	0,41	0,61	0,43	0,32
1460.674 m/z ± 0.154 Da	0,56	0,47	0,40	0,62	0,43	0,31
1461.674 m/z ± 0.154 Da	0,50	0,42	0,41	0,58	0,42	0,33
1462.674 m/z ±	0,48	0,44	0,45	0,55	0,44	0,39

0.154 Da						
1465.674 m/z ± 0.154 Da	0,47	0,37	0,39	0,56	0,39	0,31
1466.674 m/z ± 0.154 Da	0,55	0,43	0,38	0,60	0,42	0,32
1467.674 m/z ± 0.154 Da	0,52	0,41	0,39	0,57	0,42	0,33
1468.674 m/z ± 0.154 Da	0,46	0,40	0,44	0,52	0,46	0,43
1476.075 m/z ± 0.154 Da	0,54	0,55	0,51	0,51	0,49	0,47
1477.675 m/z ± 0.154 Da	0,54	0,46	0,43	0,60	0,43	0,33
1478.675 m/z ± 0.154 Da	0,55	0,47	0,43	0,59	0,45	0,35
1479.675 m/z ± 0.154 Da	0,48	0,44	0,47	0,54	0,43	0,40
1480.675 m/z ± 0.154 Da	0,50	0,48	0,46	0,54	0,48	0,44
1481.675 m/z ± 0.154 Da	0,47	0,43	0,46	0,54	0,46	0,42
1482.676 m/z ± 0.154 Da	0,50	0,47	0,47	0,53	0,46	0,43
1487.676 m/z ± 0.154 Da	0,48	0,43	0,47	0,55	0,46	0,42
1488.676 m/z ±	0,54	0,48	0,43	0,55	0,45	0,41

0.154 Da						
1493.677 m/z ± 0.154 Da	0,49	0,35	0,37	0,59	0,45	0,34
1494.677 m/z ± 0.154 Da	0,47	0,33	0,37	0,58	0,36	0,28
1495.677 m/z ± 0.154 Da	0,52	0,37	0,37	0,59	0,34	0,26
1501.677 m/z ± 0.154 Da	0,47	0,48	0,53	0,53	0,44	0,41
1502.677 m/z ± 0.154 Da	0,51	0,52	0,52	0,54	0,43	0,40
1503.677 m/z ± 0.154 Da	0,50	0,49	0,48	0,56	0,46	0,40
1504.678 m/z ± 0.154 Da	0,49	0,47	0,48	0,54	0,46	0,42
1508.678 m/z ± 0.154 Da	0,49	0,41	0,42	0,60	0,40	0,32
1509.678 m/z ± 0.154 Da	0,48	0,42	0,43	0,57	0,40	0,33
1510.678 m/z ± 0.154 Da	0,50	0,41	0,40	0,57	0,42	0,36
1511.678 m/z ± 0.154 Da	0,50	0,44	0,45	0,54	0,47	0,43
1515.679 m/z ± 0.154 Da	0,52	0,43	0,41	0,61	0,41	0,28
1516.679 m/z ±	0,52	0,45	0,42	0,61	0,43	0,32

0.154 Da					
1520.679 m/z ± 0.154 Da	0,49	0,49	0,51	0,49	0,46	0,47
1521.679 m/z ± 0.154 Da	0,47	0,48	0,54	0,48	0,48	0,51
1529.68 m/z ± 0.154 Da	0,46	0,57	0,59	0,50	0,61	0,60
1530.68 m/z ± 0.154 Da	0,46	0,42	0,45	0,57	0,43	0,36
1531.68 m/z ± 0.154 Da	0,50	0,42	0,41	0,59	0,41	0,31
1532.68 m/z ± 0.154 Da	0,45	0,39	0,44	0,55	0,42	0,36
1533.68 m/z ± 0.154 Da	0,55	0,47	0,42	0,58	0,44	0,34
1534.68 m/z ± 0.154 Da	0,51	0,44	0,43	0,57	0,47	0,40
1546.881 m/z ± 0.154 Da	0,51	0,37	0,36	0,61	0,44	0,31
1547.882 m/z ± 0.154 Da	0,45	0,33	0,37	0,61	0,43	0,33
1548.882 m/z ± 0.154 Da	0,48	0,37	0,39	0,58	0,45	0,37
1549.682 m/z ± 0.154 Da	0,54	0,44	0,43	0,56	0,43	0,37
1553.682 m/z ±	0,48	0,48	0,51	0,55	0,47	0,41

0.154 Da					
1561.883 m/z ± 0.154 Da	0,48	0,38	0,39	0,60	0,48	0,37
1562.883 m/z ± 0.154 Da	0,46	0,33	0,36	0,62	0,40	0,28
1563.883 m/z ± 0.154 Da	0,47	0,35	0,36	0,62	0,41	0,28
1564.883 m/z ± 0.154 Da	0,49	0,37	0,38	0,59	0,42	0,33
1565.883 m/z ± 0.154 Da	0,45	0,38	0,42	0,54	0,44	0,40
1567.683 m/z ± 0.154 Da	0,49	0,48	0,48	0,50	0,51	0,50
1568.683 m/z ± 0.154 Da	0,48	0,43	0,43	0,54	0,45	0,41
1569.683 m/z ± 0.154 Da	0,46	0,43	0,45	0,52	0,45	0,43
1572.684 m/z ± 0.154 Da	0,49	0,49	0,52	0,52	0,47	0,46
1573.684 m/z ± 0.154 Da	0,48	0,48	0,52	0,51	0,47	0,47
1580.684 m/z ± 0.154 Da	0,54	0,45	0,41	0,56	0,45	0,39
1581.685 m/z ± 0.154 Da	0,56	0,47	0,40	0,59	0,47	0,39
1582.685 m/z ±	0,53	0,46	0,42	0,53	0,45	0,41

0.154 Da						
1584.685 m/z ± 0.154 Da	0,47	0,43	0,45	0,55	0,46	0,40
1585.685 m/z ± 0.154 Da	0,49	0,42	0,43	0,57	0,42	0,35
1586.685 m/z ± 0.154 Da	0,57	0,47	0,42	0,61	0,44	0,32
1587.685 m/z ± 0.154 Da	0,50	0,43	0,42	0,57	0,43	0,35
1588.685 m/z ± 0.154 Da	0,52	0,46	0,44	0,55	0,43	0,39
1589.685 m/z ± 0.154 Da	0,51	0,47	0,46	0,53	0,47	0,45
1602.686 m/z ± 0.154 Da	0,52	0,47	0,44	0,56	0,44	0,37
1605.687 m/z ± 0.154 Da	0,54	0,54	0,50	0,50	0,50	0,50
1606.687 m/z ± 0.154 Da	0,51	0,51	0,48	0,51	0,49	0,49
1607.687 m/z ± 0.154 Da	0,51	0,48	0,47	0,53	0,50	0,47
1608.687 m/z ± 0.154 Da	0,50	0,47	0,44	0,52	0,46	0,44
1619.688 m/z ± 0.154 Da	0,55	0,48	0,43	0,56	0,49	0,42
1620.688 m/z ±	0,52	0,45	0,44	0,54	0,47	0,43

0.154 Da						
1621.688 m/z ± 0.154 Da	0,48	0,45	0,48	0,52	0,47	0,46
1623.688 m/z ± 0.154 Da	0,52	0,48	0,47	0,58	0,44	0,35
1624.688 m/z ± 0.154 Da	0,50	0,46	0,47	0,57	0,43	0,35
1627.689 m/z ± 0.154 Da	0,49	0,43	0,43	0,55	0,45	0,40
1628.689 m/z ± 0.154 Da	0,48	0,44	0,45	0,51	0,44	0,43
1637.89 m/z ± 0.154 Da	0,51	0,46	0,45	0,57	0,44	0,38
1648.691 m/z ± 0.154 Da	0,54	0,50	0,45	0,54	0,48	0,44
1652.891 m/z ± 0.154 Da	0,48	0,45	0,46	0,56	0,51	0,45
1655.891 m/z ± 0.154 Da	0,45	0,38	0,40	0,58	0,43	0,35
1656.891 m/z ± 0.154 Da	0,47	0,39	0,40	0,58	0,44	0,35
1657.892 m/z ± 0.154 Da	0,47	0,41	0,43	0,55	0,45	0,41
1658.892 m/z ± 0.154 Da	0,49	0,44	0,43	0,54	0,47	0,43
1665.692 m/z ±	0,48	0,45	0,47	0,52	0,47	0,45

0.154 Da						
1666.692 m/z ± 0.154 Da	0,50	0,47	0,47	0,53	0,47	0,44
1667.692 m/z ± 0.154 Da	0,50	0,48	0,49	0,52	0,48	0,46
1669.893 m/z ± 0.154 Da	0,47	0,38	0,39	0,56	0,41	0,34
1670.893 m/z ± 0.154 Da	0,48	0,37	0,37	0,59	0,38	0,29
1671.893 m/z ± 0.154 Da	0,48	0,39	0,39	0,57	0,41	0,34
1672.893 m/z ± 0.154 Da	0,48	0,41	0,43	0,54	0,44	0,39
1682.894 m/z ± 0.154 Da	0,48	0,46	0,48	0,50	0,47	0,48
1683.894 m/z ± 0.154 Da	0,45	0,40	0,44	0,54	0,46	0,43
1684.894 m/z ± 0.154 Da	0,44	0,38	0,41	0,54	0,45	0,41
1685.694 m/z ± 0.154 Da	0,48	0,45	0,44	0,56	0,42	0,36
1686.694 m/z ± 0.154 Da	0,47	0,44	0,46	0,55	0,41	0,36
1687.694 m/z ± 0.154 Da	0,49	0,45	0,46	0,56	0,44	0,38
1690.694 m/z ±	0,51	0,48	0,44	0,55	0,44	0,40

0.154 Da						
1691.895 m/z ± 0.154 Da	0,46	0,39	0,41	0,56	0,39	0,33
1692.895 m/z ± 0.154 Da	0,45	0,40	0,41	0,58	0,44	0,36
1693.695 m/z ± 0.154 Da	0,51	0,51	0,48	0,54	0,45	0,41
1694.695 m/z ± 0.154 Da	0,54	0,54	0,47	0,57	0,45	0,39
1706.696 m/z ± 0.154 Da	0,50	0,37	0,37	0,57	0,44	0,37
1707.696 m/z ± 0.154 Da	0,53	0,41	0,38	0,58	0,45	0,36
1708.696 m/z ± 0.154 Da	0,51	0,41	0,39	0,57	0,44	0,35
1709.696 m/z ± 0.154 Da	0,48	0,41	0,43	0,55	0,43	0,39
1710.896 m/z ± 0.154 Da	0,46	0,41	0,42	0,60	0,36	0,26
1711.896 m/z ± 0.154 Da	0,52	0,46	0,43	0,63	0,39	0,25
1712.897 m/z ± 0.154 Da	0,49	0,44	0,45	0,60	0,39	0,30
1723.697 m/z ± 0.154 Da	0,51	0,48	0,46	0,57	0,42	0,35
1724.698 m/z ±	0,54	0,52	0,46	0,58	0,43	0,35

0.154 Da						
1728.698 m/z ± 0.154 Da	0,52	0,48	0,44	0,56	0,45	0,40
1729.698 m/z ± 0.154 Da	0,52	0,48	0,47	0,55	0,45	0,40
1731.698 m/z ± 0.154 Da	0,52	0,52	0,49	0,55	0,45	0,41
1742.699 m/z ± 0.154 Da	0,53	0,44	0,39	0,57	0,46	0,38
1743.699 m/z ± 0.154 Da	0,53	0,43	0,42	0,59	0,47	0,38
1744.699 m/z ± 0.154 Da	0,51	0,42	0,41	0,58	0,44	0,35
1745.699 m/z ± 0.154 Da	0,53	0,46	0,43	0,59	0,44	0,34
1746.7 m/z ± 0.154 Da	0,53	0,48	0,44	0,57	0,43	0,36
1751.9 m/z ± 0.154 Da	0,46	0,38	0,41	0,56	0,42	0,35
1752.9 m/z ± 0.154 Da	0,49	0,39	0,39	0,59	0,44	0,34
1753.9 m/z ± 0.154 Da	0,46	0,39	0,42	0,56	0,46	0,39
1766.901 m/z ± 0.154 Da	0,47	0,38	0,40	0,57	0,46	0,37
1767.902 m/z ±	0,49	0,39	0,38	0,57	0,47	0,39

0.154 Da						
1768.902 m/z ± 0.154 Da	0,47	0,39	0,42	0,54	0,47	0,42
1775.902 m/z ± 0.154 Da	0,50	0,41	0,40	0,59	0,46	0,35
1776.902 m/z ± 0.154 Da	0,49	0,40	0,42	0,59	0,46	0,35
1777.902 m/z ± 0.154 Da	0,47	0,38	0,41	0,56	0,44	0,37
1782.703 m/z ± 0.154 Da	0,47	0,46	0,49	0,55	0,46	0,40
1790.904 m/z ± 0.154 Da	0,47	0,39	0,42	0,57	0,36	0,27
1791.904 m/z ± 0.154 Da	0,51	0,42	0,41	0,59	0,36	0,26
1797.904 m/z ± 0.154 Da	0,49	0,46	0,44	0,57	0,46	0,40
1798.904 m/z ± 0.154 Da	0,50	0,44	0,44	0,57	0,45	0,38
1816.906 m/z ± 0.154 Da	0,45	0,33	0,36	0,59	0,44	0,32
1817.906 m/z ± 0.154 Da	0,49	0,36	0,36	0,60	0,45	0,31
1818.906 m/z ± 0.154 Da	0,49	0,37	0,38	0,60	0,46	0,34
1819.906 m/z ±	0,49	0,42	0,42	0,59	0,47	0,38

0.154 Da						
1820.906 m/z ± 0.154 Da	0,52	0,48	0,46	0,57	0,47	0,39
1821.906 m/z ± 0.154 Da	0,52	0,50	0,47	0,56	0,46	0,40
1822.907 m/z ± 0.154 Da	0,50	0,49	0,48	0,54	0,46	0,42
1832.907 m/z ± 0.154 Da	0,50	0,37	0,36	0,60	0,42	0,30
1833.908 m/z ± 0.154 Da	0,49	0,35	0,35	0,60	0,39	0,28
1834.908 m/z ± 0.154 Da	0,46	0,35	0,38	0,59	0,42	0,32
1835.908 m/z ± 0.154 Da	0,46	0,37	0,40	0,58	0,44	0,34
1836.908 m/z ± 0.154 Da	0,47	0,40	0,42	0,57	0,46	0,37
1848.909 m/z ± 0.154 Da	0,46	0,39	0,43	0,56	0,46	0,41
1850.909 m/z ± 0.154 Da	0,52	0,46	0,44	0,59	0,44	0,34
1851.909 m/z ± 0.154 Da	0,48	0,43	0,45	0,58	0,43	0,34
1852.909 m/z ± 0.154 Da	0,45	0,41	0,45	0,56	0,45	0,38
1854.909 m/z ±	0,48	0,42	0,42	0,56	0,46	0,39

0.154 Da						
1855.91 m/z ± 0.154 Da	0,49	0,42	0,43	0,55	0,46	0,40
1856.91 m/z ± 0.154 Da	0,47	0,41	0,43	0,54	0,47	0,42
1867.911 m/z ± 0.154 Da	0,45	0,43	0,46	0,55	0,43	0,39
1869.911 m/z ± 0.154 Da	0,52	0,47	0,44	0,57	0,45	0,39
1870.911 m/z ± 0.154 Da	0,46	0,41	0,45	0,55	0,47	0,41
1871.911 m/z ± 0.154 Da	0,47	0,42	0,45	0,54	0,45	0,40
1888.913 m/z ± 0.154 Da	0,51	0,48	0,46	0,59	0,48	0,37
1889.913 m/z ± 0.154 Da	0,47	0,44	0,45	0,57	0,46	0,38
1922.916 m/z ± 0.154 Da	0,49	0,42	0,43	0,57	0,44	0,37
1923.916 m/z ± 0.154 Da	0,52	0,44	0,42	0,60	0,41	0,30
1924.916 m/z ± 0.154 Da	0,49	0,41	0,41	0,60	0,43	0,32
1925.916 m/z ± 0.154 Da	0,48	0,41	0,43	0,57	0,42	0,34
1950.918 m/z ±	0,47	0,47	0,49	0,48	0,46	0,49

0.154 Da						
1951.918 m/z ± 0.154 Da	0,47	0,48	0,51	0,49	0,47	0,49
1952.918 m/z ± 0.154 Da	0,46	0,47	0,48	0,48	0,47	0,49
1961.919 m/z ± 0.154 Da	0,49	0,42	0,42	0,59	0,45	0,34
1962.919 m/z ± 0.154 Da	0,50	0,43	0,43	0,60	0,46	0,34
1963.919 m/z ± 0.154 Da	0,49	0,43	0,43	0,58	0,47	0,38
1964.919 m/z ± 0.154 Da	0,47	0,42	0,45	0,60	0,47	0,36
1966.92 m/z ± 0.154 Da	0,49	0,48	0,51	0,56	0,48	0,41
1967.92 m/z ± 0.154 Da	0,47	0,48	0,51	0,51	0,47	0,46
1992.922 m/z ± 0.154 Da	0,47	0,47	0,49	0,52	0,48	0,47
1993.922 m/z ± 0.154 Da	0,50	0,49	0,49	0,54	0,46	0,42
2003.923 m/z ± 0.154 Da	0,51	0,43	0,41	0,62	0,46	0,33
2004.923 m/z ± 0.154 Da	0,53	0,43	0,41	0,58	0,43	0,35
2005.923 m/z ±	0,50	0,41	0,42	0,56	0,44	0,38

0.154 Da						
2026.925 m/z ± 0.154 Da	0,49	0,50	0,50	0,55	0,53	0,48
2027.925 m/z ± 0.154 Da	0,53	0,52	0,45	0,57	0,52	0,45
2028.925 m/z ± 0.154 Da	0,52	0,49	0,47	0,55	0,50	0,44
2038.926 m/z ± 0.154 Da	0,49	0,43	0,45	0,53	0,47	0,44
2039.926 m/z ± 0.154 Da	0,47	0,39	0,41	0,54	0,48	0,44
2040.926 m/z ± 0.154 Da	0,51	0,44	0,42	0,55	0,48	0,44
2041.926 m/z ± 0.154 Da	0,52	0,46	0,45	0,56	0,44	0,38
2042.927 m/z ± 0.154 Da	0,48	0,45	0,47	0,55	0,45	0,41
2056.128 m/z ± 0.154 Da	0,47	0,38	0,39	0,58	0,50	0,42
2056.928 m/z ± 0.154 Da	0,49	0,42	0,42	0,58	0,47	0,38
2057.928 m/z ± 0.154 Da	0,54	0,45	0,40	0,59	0,47	0,37
2058.928 m/z ± 0.154 Da	0,50	0,43	0,42	0,58	0,49	0,40
2059.928 m/z ±	0,48	0,42	0,43	0,55	0,49	0,44

0.154 Da					
2072.129 m/z ± 0.154 Da	0,46	0,36	0,37	0,60	0,47	0,36
2073.129 m/z ± 0.154 Da	0,44	0,35	0,37	0,59	0,45	0,35
2074.129 m/z ± 0.154 Da	0,50	0,40	0,40	0,58	0,47	0,38
2078.93 m/z ± 0.154 Da	0,49	0,45	0,45	0,55	0,49	0,43
2079.93 m/z ± 0.154 Da	0,51	0,46	0,45	0,56	0,46	0,39
2088.931 m/z ± 0.154 Da	0,51	0,47	0,46	0,56	0,45	0,39
2089.931 m/z ± 0.154 Da	0,54	0,46	0,44	0,57	0,43	0,36
2094.931 m/z ± 0.154 Da	0,48	0,43	0,43	0,60	0,45	0,33
2095.931 m/z ± 0.154 Da	0,49	0,45	0,46	0,59	0,44	0,33
2096.931 m/z ± 0.154 Da	0,49	0,47	0,47	0,58	0,46	0,38
2104.132 m/z ± 0.154 Da	0,44	0,36	0,40	0,62	0,39	0,26
2105.132 m/z ± 0.154 Da	0,44	0,36	0,41	0,62	0,38	0,24
2105.932 m/z ±	0,49	0,42	0,42	0,57	0,41	0,34

0.154 Da						
2106.932 m/z ± 0.154 Da	0,50	0,43	0,42	0,55	0,43	0,38
2115.133 m/z ± 0.154 Da	0,48	0,36	0,36	0,61	0,43	0,28
2116.133 m/z ± 0.154 Da	0,49	0,37	0,38	0,61	0,42	0,29
2117.133 m/z ± 0.154 Da	0,49	0,40	0,41	0,59	0,45	0,34
2118.133 m/z ± 0.154 Da	0,52	0,43	0,38	0,60	0,47	0,36
2138.135 m/z ± 0.154 Da	0,48	0,41	0,41	0,55	0,47	0,41
2154.137 m/z ± 0.154 Da	0,44	0,42	0,46	0,56	0,46	0,39
2163.938 m/z ± 0.154 Da	0,49	0,43	0,43	0,60	0,44	0,34
2164.938 m/z ± 0.154 Da	0,53	0,48	0,43	0,59	0,43	0,33
2165.938 m/z ± 0.154 Da	0,52	0,48	0,45	0,57	0,45	0,38
2198.941 m/z ± 0.154 Da	0,49	0,44	0,42	0,56	0,49	0,43
2199.941 m/z ± 0.154 Da	0,50	0,42	0,42	0,57	0,46	0,39
2200.941 m/z ±	0,48	0,43	0,45	0,54	0,49	0,44

0.154 Da					
2215.942 m/z ± 0.154 Da	0,52	0,40	0,36	0,60	0,39	0,29
2217.142 m/z ± 0.154 Da	0,48	0,34	0,34	0,59	0,35	0,26
2218.142 m/z ± 0.154 Da	0,45	0,33	0,37	0,57	0,38	0,31
2219.143 m/z ± 0.154 Da	0,43	0,34	0,40	0,53	0,43	0,40
2437.162 m/z ± 0.154 Da	0,51	0,48	0,47	0,53	0,50	0,47
2454.164 m/z ± 0.154 Da	0,48	0,43	0,45	0,56	0,53	0,47
2455.164 m/z ± 0.154 Da	0,51	0,45	0,46	0,56	0,51	0,45
2456.164 m/z ± 0.154 Da	0,51	0,46	0,43	0,58	0,51	0,42
2457.164 m/z ± 0.154 Da	0,50	0,45	0,44	0,55	0,51	0,46
2461.165 m/z ± 0.154 Da	0,48	0,43	0,46	0,62	0,46	0,33
2462.165 m/z ± 0.154 Da	0,47	0,43	0,44	0,61	0,44	0,31
2463.165 m/z ± 0.154 Da	0,52	0,46	0,45	0,62	0,45	0,32
2470.165 m/z ±	0,53	0,46	0,44	0,55	0,48	0,43

0.154 Da						
2471.165 m/z ± 0.154 Da	0,48	0,44	0,43	0,55	0,50	0,44
2477.166 m/z ± 0.154 Da	0,50	0,44	0,43	0,61	0,43	0,31
2478.166 m/z ± 0.154 Da	0,47	0,42	0,44	0,61	0,43	0,32
2479.166 m/z ± 0.154 Da	0,49	0,45	0,47	0,58	0,44	0,36
2493.167 m/z ± 0.154 Da	0,49	0,43	0,42	0,59	0,45	0,35
2498.168 m/z ± 0.154 Da	0,50	0,46	0,45	0,55	0,49	0,44
2510.169 m/z ± 0.154 Da	0,51	0,45	0,43	0,55	0,48	0,42
2511.169 m/z ± 0.154 Da	0,47	0,44	0,47	0,53	0,51	0,48
2520.17 m/z ± 0.154 Da	0,48	0,45	0,46	0,52	0,51	0,48
2521.17 m/z ± 0.154 Da	0,49	0,46	0,46	0,53	0,50	0,47
2674.184 m/z ± 0.154 Da	0,51	0,51	0,50	0,58	0,52	0,44
2675.184 m/z ± 0.154 Da	0,50	0,47	0,45	0,60	0,51	0,40
2676.184 m/z ±	0,54	0,49	0,46	0,62	0,50	0,38

0.154 Da					
2690.185 m/z ± 0.154 Da	0,53	0,49	0,46	0,59	0,52	0,40
2691.185 m/z ± 0.154 Da	0,50	0,47	0,46	0,59	0,50	0,39
2692.186 m/z ± 0.154 Da	0,51	0,47	0,46	0,59	0,48	0,37
2694.186 m/z ± 0.154 Da	0,45	0,44	0,47	0,53	0,49	0,47
2704.187 m/z ± 0.154 Da	0,49	0,47	0,48	0,54	0,53	0,48
2705.187 m/z ± 0.154 Da	0,52	0,46	0,44	0,58	0,54	0,45
2706.187 m/z ± 0.154 Da	0,51	0,42	0,41	0,58	0,54	0,44
2707.187 m/z ± 0.154 Da	0,53	0,44	0,43	0,58	0,55	0,45
2708.187 m/z ± 0.154 Da	0,49	0,42	0,43	0,54	0,53	0,49
2721.188 m/z ± 0.154 Da	0,47	0,42	0,44	0,55	0,48	0,41
2727.189 m/z ± 0.154 Da	0,50	0,48	0,47	0,53	0,51	0,49
2728.189 m/z ± 0.154 Da	0,51	0,46	0,45	0,52	0,51	0,48
2729.189 m/z ±	0,47	0,45	0,47	0,51	0,51	0,49

0.154 Da					
2730.189 m/z ± 0.154 Da	0,50	0,47	0,49	0,51	0,51	0,50
2744.19 m/z ± 0.154 Da	0,49	0,46	0,47	0,54	0,54	0,48
2745.19 m/z ± 0.154 Da	0,48	0,46	0,47	0,52	0,52	0,50
2869.402 m/z ± 0.154 Da	0,51	0,39	0,37	0,62	0,43	0,29
2870.402 m/z ± 0.154 Da	0,49	0,36	0,37	0,61	0,40	0,26
2871.402 m/z ± 0.154 Da	0,47	0,34	0,36	0,61	0,42	0,29
2872.402 m/z ± 0.154 Da	0,46	0,36	0,39	0,58	0,43	0,34
2885.403 m/z ± 0.154 Da	0,47	0,45	0,47	0,57	0,60	0,53
2886.403 m/z ± 0.154 Da	0,46	0,47	0,50	0,54	0,61	0,56
2887.403 m/z ± 0.154 Da	0,49	0,49	0,52	0,55	0,61	0,55
2950.409 m/z ± 0.154 Da	0,48	0,41	0,42	0,62	0,41	0,27
2951.409 m/z ± 0.154 Da	0,47	0,39	0,40	0,62	0,40	0,27
2952.409 m/z ±	0,45	0,37	0,41	0,63	0,40	0,26

0.154 Da					
2953.409 m/z ± 0.154 Da	0,49	0,42	0,41	0,62	0,44	0,31
2959.41 m/z ± 0.154 Da	0,46	0,37	0,40	0,60	0,51	0,40
2960.41 m/z ± 0.154 Da	0,43	0,34	0,38	0,60	0,50	0,38
2961.41 m/z ± 0.154 Da	0,45	0,35	0,38	0,62	0,52	0,38
2962.41 m/z ± 0.154 Da	0,44	0,35	0,38	0,57	0,51	0,44
2963.41 m/z ± 0.154 Da	0,48	0,39	0,39	0,56	0,51	0,45
2980.412 m/z ± 0.154 Da	0,48	0,48	0,49	0,51	0,51	0,49
3084.421 m/z ± 0.154 Da	0,45	0,42	0,44	0,53	0,50	0,47
3085.421 m/z ± 0.154 Da	0,49	0,41	0,40	0,59	0,50	0,40
3086.421 m/z ± 0.154 Da	0,47	0,39	0,41	0,55	0,48	0,42
3087.421 m/z ± 0.154 Da	0,46	0,42	0,43	0,53	0,50	0,47
3100.423 m/z ± 0.154 Da	0,43	0,34	0,37	0,58	0,55	0,46
3101.423 m/z ±	0,44	0,33	0,37	0,59	0,53	0,42

0.154 Da					
3102.423 m/z ± 0.154 Da	0,43	0,33	0,35	0,61	0,55	0,42
3103.423 m/z ± 0.154 Da	0,42	0,32	0,36	0,61	0,56	0,44
3104.423 m/z ± 0.154 Da	0,46	0,35	0,37	0,56	0,53	0,46
3122.425 m/z ± 0.154 Da	0,46	0,44	0,47	0,53	0,53	0,50
3123.425 m/z ± 0.154 Da	0,47	0,41	0,44	0,55	0,52	0,48
3124.425 m/z ± 0.154 Da	0,46	0,41	0,44	0,54	0,51	0,47
3139.426 m/z ± 0.154 Da	0,46	0,43	0,44	0,56	0,52	0,45
3141.426 m/z ± 0.154 Da	0,47	0,45	0,46	0,54	0,51	0,46
3168.429 m/z ± 0.154 Da	0,52	0,51	0,50	0,52	0,52	0,50
3169.429 m/z ± 0.154 Da	0,50	0,49	0,50	0,51	0,50	0,49
3171.429 m/z ± 0.154 Da	0,50	0,47	0,49	0,52	0,49	0,47
3175.43 m/z ± 0.154 Da	0,54	0,51	0,48	0,52	0,50	0,47
3178.43 m/z ±	0,52	0,51	0,50	0,52	0,50	0,48

0.154 Da					
3179.23 m/z ± 0.154 Da	0,48	0,50	0,51	0,51	0,51	0,51
3180.43 m/z ± 0.154 Da	0,49	0,50	0,50	0,52	0,49	0,48
3181.43 m/z ± 0.154 Da	0,53	0,52	0,49	0,52	0,49	0,46
3182.43 m/z ± 0.154 Da	0,49	0,49	0,51	0,51	0,50	0,49
3183.43 m/z ± 0.154 Da	0,49	0,48	0,49	0,52	0,52	0,50
3184.43 m/z ± 0.154 Da	0,50	0,49	0,47	0,52	0,52	0,49
3185.43 m/z ± 0.154 Da	0,51	0,48	0,44	0,53	0,51	0,48
3186.431 m/z ± 0.154 Da	0,51	0,48	0,45	0,54	0,52	0,48
3187.431 m/z ± 0.154 Da	0,51	0,48	0,47	0,53	0,51	0,48
3188.431 m/z ± 0.154 Da	0,51	0,49	0,50	0,53	0,51	0,48
3189.631 m/z ± 0.154 Da	0,52	0,52	0,49	0,51	0,50	0,49
3190.431 m/z ± 0.154 Da	0,49	0,50	0,48	0,50	0,50	0,50
3191.431 m/z ±	0,52	0,51	0,48	0,51	0,50	0,49

0.154 Da						
3192.631 m/z ± 0.154 Da	0,49	0,50	0,52	0,50	0,52	0,51
3193.231 m/z ± 0.154 Da	0,47	0,50	0,52	0,50	0,52	0,52
3193.831 m/z ± 0.154 Da	0,49	0,51	0,53	0,50	0,52	0,52
3194.431 m/z ± 0.154 Da	0,51	0,51	0,49	0,51	0,49	0,48
3195.431 m/z ± 0.154 Da	0,51	0,50	0,51	0,51	0,49	0,48
3196.431 m/z ± 0.154 Da	0,50	0,49	0,48	0,53	0,50	0,48
3197.432 m/z ± 0.154 Da	0,49	0,51	0,49	0,52	0,50	0,47
3198.432 m/z ± 0.154 Da	0,50	0,50	0,50	0,53	0,50	0,48
3199.432 m/z ± 0.154 Da	0,51	0,51	0,49	0,52	0,51	0,49

Supplementary Table S2. Peptides identified by nanoLC-MS/MS

Retention time (min)	Charge	m/z	Measured mass	Mass error (u)	Mass error (ppm)	Score	Sequence	Modifications	Accession	Description
31,314	2	480,758	959,502	-0,001	-1,397	49,3	VTDALNATR		CH60_HUMAN	60 kDa heat shock protein, mitochondrial
53,593	2	900,991	1799,967	0,000	-0,086	27,26	LAPDYDALDVANKIGII		RL23A_HUMAN	60S ribosomal protein L23a
51,270	3	602,669	1804,987	-0,001	-0,566	29,3	SVFALTNGIYPHKLVF		RL6_HUMAN	60S ribosomal protein L6
32,339	2	614,816	1227,618	-0,003	-2,326	26,78	VEIIANDQGNR		GRP78_HUMAN	78 kDa glucose-regulated protein
41,586	2	658,821	1315,628	-0,001	-0,852	52,08	NELESYAYSLK		GRP78_HUMAN	78 kDa glucose-regulated protein
43,412	2	764,876	1527,737	-0,002	-1,503	37,32	AKFEELNMDLFR	[8] Oxidation (M)	GRP78_HUMAN	78 kDa glucose-regulated protein
31,246	2	488,727	975,440	-0,001	-1,518	88,56	AGFAGDDAPR		ACTA_HUMAN	Actin, aortic smooth muscle
37,841	2	565,776	1129,538	-0,002	-2,062	39,53	GYSFVTTAER		ACTA_HUMAN	Actin, aortic smooth muscle
38,604	2	581,312	1160,610	-0,001	-0,887	27,67	EITALAPSTMK		ACTA_HUMAN	Actin, aortic smooth muscle
31,651	2	586,288	1170,561	-0,002	-2,135	59,23	HQGVMVGMGQK		ACTA_HUMAN	Actin, aortic smooth muscle

35,588	2	589,309	1176,604	-0,002	-1,464	49,97	EITALAPSTMK	[10] Oxidation (M)	ACTA_HUMAN	Actin, aortic smooth muscle
30,739	2	594,285	1186,555	-0,003	-2,802	32,36	HQGVMVGMGQK	[8] Oxidation (M)	ACTA_HUMAN	Actin, aortic smooth muscle
31,010	2	594,285	1186,556	-0,003	-2,392	59,62	HQGVMVGMGQK	[5] Oxidation (M)	ACTA_HUMAN	Actin, aortic smooth muscle
30,567	2	599,763	1197,512	-0,003	-2,414	57,19	DSYVGDEAQSK		ACTA_HUMAN	Actin, aortic smooth muscle
40,077	2	599,855	1197,696	-0,002	-2,086	41,77	AVFPSIVGRPR		ACTA_HUMAN	Actin, aortic smooth muscle
40,077	3	400,241	1197,701	0,003	2,297	33,51	AVFPSIVGRPR		ACTA_HUMAN	Actin, aortic smooth muscle
30,363	2	602,283	1202,551	-0,003	-2,472	41,92	HQGVMVGMGQK	[5] Oxidation (M) ǀ [8] Oxidation (M)	ACTA_HUMAN	Actin, aortic smooth muscle
30,567	2	677,814	1353,613	-0,003	-2,204	48,47	DSYVGDEAQSKR		ACTA_HUMAN	Actin, aortic smooth muscle
33,468	2	790,904	1579,793	-0,002	-1,133	43,04	MQKEITALAPSTMK	[1] Oxidation (M) ǀ [13] Oxidation (M)	ACTA_HUMAN	Actin, aortic smooth muscle
45,436	2	895,949	1789,884	-0,001	-0,339	122,91	SYELPDGQVITIGNER		ACTA_HUMAN	Actin, aortic smooth muscle
44,400	2	895,949	1789,884	-0,001	-0,313	28,06	SYELPDGQVITIGNER		ACTA_HUMAN	Actin, aortic smooth muscle
41,285	2	978,525	1955,036	-0,001	-0,289	77,54	VAPEEHPTLLTEAPLNPK		ACTA_HUMAN	Actin, aortic smooth muscle

41,285	3	652,687	1955,039	0,002	1,149	46,13	VAPEEHPTLLTE APLNPK		ACTA_HUMAN	Actin, aortic smooth muscle
43,392	3	719,382	2155,125	-0,004	-1,644	30,05	AGFAGDDAPRA VFPSIVGRPR		ACTA_HUMAN	Actin, aortic smooth muscle
46,922	2	1114,53 6	2227,058	0,000	0,202	86,08	DLYANNVLSGG TTMYPGIADR		ACTA_HUMAN	Actin, aortic smooth muscle
44,744	2	1122,53 4	2243,053	0,000	-0,024	109,78	DLYANNVLSGG TTMYPGIADR	[14] Oxidation (M)	ACTA_HUMAN	Actin, aortic smooth muscle
34,926	3	784,363	2350,067	-0,001	-0,637	40,56	HQGVMVGMGQ KDSYVGDEAQSK		ACTA_HUMAN	Actin, aortic smooth muscle
41,782	3	791,390	2371,147	-0,001	-0,420	35,62	KDLYANNVLSG GTTMYPGIADR	[15] Oxidation (M)	ACTA_HUMAN	Actin, aortic smooth muscle
29,220	2	468,796	935,577	-0,003	-3,239	32,28	KPPGSLLPK		ACD11_HUMAN	Acyl-CoA dehydrogenase family member 11
29,204	3	312,867	935,579	-0,001	-1,444	37,77	KPPGSLLPK		ACD11_HUMAN	Acyl-CoA dehydrogenase family member 11
43,392	2	519,273	1036,532	-0,002	-2,070	30,7	GFLIDGYPR		KAD5_HUMAN	Adenylate kinase isoenzyme 5
31,864	2	671,892	1341,770	-0,003	-1,962	32,68	VIQVAAGSSNLK R		ALDH2_HUMAN	Aldehyde dehydrogenase, mitochondrial
29,612	2	450,747	899,480	-0,003	-2,961	45,24	KQVSGPER		CRYAB_HUMAN	Alpha-crystallin B chain
42,114	2	461,256	920,498	0,001	1,358	28,35	FSVNLDVK		CRYAB_HUMAN	Alpha-crystallin B

											chain
53,880	3	875,464	2623,369	-0,001	-0,393	39,47	IPADVDPLTITSSLSSDGVLTVNGPR			CRYAB_HUMAN	Alpha-crystallin B chain
53,880	2	1312,692	2623,370	-0,001	-0,284	34,27	IPADVDPLTITSSLSSDGVLTVNGPR			CRYAB_HUMAN	Alpha-crystallin B chain
52,480	3	981,852	2942,534	-0,001	-0,355	33,25	YRIPADVDPLTITSSLSSDGVLTVNGPR			CRYAB_HUMAN	Alpha-crystallin B chain
52,872	3	981,852	2942,534	-0,001	-0,307	62,15	YRIPADVDPLTITSSLSSDGVLTVNGPR			CRYAB_HUMAN	Alpha-crystallin B chain
48,054	2	902,975	1803,936	-0,001	-0,371	39,53	AAVPSGASTGIYEALELR			ENOA_HUMAN	Alpha-enolase
44,894	2	580,322	1158,630	0,013	10,811	35,49	MQKPQGSLVR	[1] (M)	Oxidation	SIA7F_HUMAN	Alpha-N-acetylgalactosaminide alpha-2,6-sialyltransferase 6
38,874	2	438,769	875,523	0,000	-0,435	26,1	ALQLYLR			AL2CL_HUMAN	ALS2 C-terminal-like protein
39,830	2	501,302	1000,590	-0,002	-1,897	37,76	VLTEIIASR			ANXA5_HUMAN	Annexin A5
56,982	2	852,954	1703,893	-0,001	-0,426	75,24	GLGTDEESILTLLTSR			ANXA5_HUMAN	Annexin A5
59,363	3	630,682	1889,024	0,009	4,934	26,91	KVTEGVVDVIVYPSAADK			A1CF_HUMAN	APOBEC1 complementation

									factor
35,449	2	484,779	967,543	-0,002	-2,190	37,46	LGPLVEQGR	APOE_HUMAN	Apolipoprotein E
42,971	2	830,930	1659,846	-0,001	-0,690	52,41	NLDYVATSIHEA VTK	AATC_HUMAN	Aspartate aminotransferase, cytoplasmic
35,868	2	362,230	722,445	0,001	1,083	25,04	APGIIPR	ATPA_HUMAN	ATP synthase subunit alpha, mitochondrial
40,077	2	500,791	999,568	-0,003	-3,246	47,93	VLSIGDGIAR	ATPA_HUMAN	ATP synthase subunit alpha, mitochondrial
39,653	2	560,863	1119,711	-0,001	-1,293	42,95	VGLKAPGIIPR	ATPA_HUMAN	ATP synthase subunit alpha, mitochondrial
39,653	3	374,245	1119,713	0,000	0,141	51,21	VGLKAPGIIPR	ATPA_HUMAN	ATP synthase subunit alpha, mitochondrial
34,419	2	615,345	1228,675	-0,002	-1,873	27	ELIIGDRQTGK	ATPA_HUMAN	ATP synthase subunit alpha, mitochondrial
36,828	2	679,876	1357,737	-0,001	-1,017	34,89	ISVREPMQTGIK	ATPA_HUMAN	ATP synthase subunit alpha, mitochondrial
45,763	3	640,369	1918,087	-0,002	-1,117	45,07	VLDSGAPIKIPVG PETLGR	ATPB_HUMAN	ATP synthase subunit beta, mitochondrial
40,005	2	507,789	1013,563	-0,002	-2,331	25,75	LGTVPQFPR	PGBM_HUMAN	Basement membrane-specific heparan sulfate proteoglycan core protein
36,767	2	507,818	1013,621	-0,002	-1,810	25,78	GTLIIRDVK	PGBM_HUMAN	Basement membrane-specific heparan sulfate proteoglycan

										core protein
42,771	2	563,826	1125,638	-0,002	-1,536	32,56	LEGDTLIIPR		PGBM_HUMAN	Basement membrane-specific heparan sulfate proteoglycan core protein
35,379	3	563,327	1686,960	-0,004	-2,419	47,44	VGGHLRPGIVQSGGVVR		PGBM_HUMAN	Basement membrane-specific heparan sulfate proteoglycan core protein
57,828	3	599,009	1794,005	-0,003	-1,501	39,06	EHLLMALAGIDTLLIR	[5] Oxidation (M)	PGBM_HUMAN	Basement membrane-specific heparan sulfate proteoglycan core protein
47,467	4	613,818	2451,243	-0,002	-0,932	34,23	HLISTHFAPGDFQGFALVNPQR		PGBM_HUMAN	Basement membrane-specific heparan sulfate proteoglycan core protein
34,575	2	507,753	1013,491	-0,003	-2,509	28,22	FFVSSSQGR		BASI_HUMAN	Basigin
31,651	2	586,288	1170,561	-0,002	-2,135	59,23	HQGVMVGMGQK		ACTBL_HUMAN	Beta-actin-like protein 2
30,739	2	594,285	1186,555	-0,003	-2,802	32,36	HQGVMVGMGQK	[8] Oxidation (M)	ACTBL_HUMAN	Beta-actin-like protein 2
31,010	2	594,285	1186,556	-0,003	-2,392	59,62	HQGVMVGMGQK	[5] Oxidation (M)	ACTBL_HUMAN	Beta-actin-like protein 2
30,363	2	602,283	1202,551	-0,003	-2,472	41,92	HQGVMVGMGQK	[5] Oxidation (M) | [8]	ACTBL_HUMAN	Beta-actin-like protein 2

							Oxidation (M)		
45,436	2	895,949	1789,884	-0,001	-0,339	122,91	SYELPDGQVITIG NER	ACTBL_HUMAN	Beta-actin-like protein 2
44,400	2	895,949	1789,884	-0,001	-0,313	28,06	SYELPDGQVITIG NER	ACTBL_HUMAN	Beta-actin-like protein 2
36,733	2	690,859	1379,703	-0,001	-1,072	28,41	GNPTVEVDLHT AK	ENOB_HUMAN	Beta-enolase
48,054	2	902,975	1803,936	-0,001	-0,371	39,53	AAVPSGASTGIY EALELR	ENOB_HUMAN	Beta-enolase
32,875	2	504,290	1006,565	-0,002	-2,445	42,57	LGLGHNQIR	PGS1_HUMAN	Biglycan
32,875	3	336,530	1006,567	0,000	0,057	26,28	LGLGHNQIR	PGS1_HUMAN	Biglycan
49,382	3	676,380	2026,119	-0,002	-0,968	31,14	NHLVEIPPNLPSS LVELR	PGS1_HUMAN	Biglycan
47,100	2	782,956	1563,898	0,000	-0,220	31,49	SIVVSPILIPENQR	CAD13_HUMAN	Cadherin-13
44,869	2	918,925	1835,835	-0,001	-0,411	32,84	MTAFDADDPAT DNALLR	CAD13_HUMAN	Cadherin-13
40,928	2	508,790	1015,565	-0,001	-0,928	36,19	ISDILNSVR	KCC2B_HUMAN	Calcium/calmodulin-dependent protein kinase type II subunit beta
35,059	2	438,732	875,449	-0,001	-1,112	59,95	LPASFDAR	CATB_HUMAN	Cathepsin B
46,619	2	491,320	980,626	-0,001	-1,096	32,21	LLLPTPTVK	CCAR1_HUMAN	Cell division cycle and apoptosis regulator protein 1
35,117	4	573,792	2291,138	0,015	6,422	30,26	CRAEDTLFQAPP ALANGAHPGR	CJ090_HUMAN	Centrosomal protein C10orf90

39,404	2	623,355	1244,695	-0,002	-1,760	26,89	KTLLSNLEEAK		CLUS_HUMAN	Clusterin
33,126	2	473,253	944,491	-0,001	-1,277	30,15	LSENVIDR		CHCH3_HUMAN	Coiled-coil-helix-coiled-coil-helix domain-containing protein 3, mitochondrial
30,008	2	418,722	835,429	-0,001	-1,505	49,08	GPAGPQGPR		CO1A1_HUMAN	Collagen alpha-1(I) chain
34,480	2	426,216	850,418	-0,001	-0,963	43,88	GFSGLDGAK		CO1A1_HUMAN	Collagen alpha-1(I) chain
29,729	2	434,716	867,418	-0,002	-2,089	34,63	GEAGPQGPR		CO1A1_HUMAN	Collagen alpha-1(I) chain
30,105	2	443,722	885,429	-0,002	-1,925	47,39	GSEGPQGVR		CO1A1_HUMAN	Collagen alpha-1(I) chain
38,272	2	581,289	1160,563	-0,001	-1,222	34,7	GQAGVMGFPGPK	[6] Oxidation (M)	CO1A1_HUMAN	Collagen alpha-1(I) chain
40,959	2	479,289	956,564	-0,001	-1,429	51,43	IALVITDGR		CO6A1_HUMAN	Collagen alpha-1(VI) chain
46,133	2	966,507	1930,999	-0,001	-0,402	52,12	LLLFSDGNSQGATPAAIEK		CO6A1_HUMAN	Collagen alpha-1(VI) chain
29,760	2	393,198	784,382	-0,001	-1,519	48,12	GDQGPVGR		CO1A2_HUMAN	Collagen alpha-2(I) chain
29,812	2	448,234	894,454	-0,002	-2,484	39,01	GPAGPSGPAGK		CO1A2_HUMAN	Collagen alpha-2(I) chain
29,988	2	612,308	1222,602	-0,003	-2,602	65,83	GPAGPSGPAGKDGR		CO1A2_HUMAN	Collagen alpha-2(I) chain

32,996	2	781,899	1561,783	-0,002	-0,980	87,57	GETGPSGPVGPAGAVGPR		CO1A2_HUMAN	Collagen alpha-2(I) chain
31,314	3	676,342	2026,004	-0,005	-2,623	39,23	HGNRGETGPSGPVGPAGAVGPR		CO1A2_HUMAN	Collagen alpha-2(I) chain
39,888	2	607,848	1213,681	-0,001	-1,030	44,56	LFAVAPNQNLK		CO6A2_HUMAN	Collagen alpha-2(VI) chain
36,330	2	651,327	1300,639	-0,002	-1,345	34,24	DIASTPHELYR		CO6A2_HUMAN	Collagen alpha-2(VI) chain
30,773	2	359,216	716,417	-0,001	-1,092	33,63	VTQLTR		CO6A3_HUMAN	Collagen alpha-3(VI) chain
34,891	2	367,221	732,428	0,000	-0,203	34,33	FAQVIR		CO6A3_HUMAN	Collagen alpha-3(VI) chain
38,359	2	403,232	804,450	0,000	0,126	36,43	ALEFVAR		CO6A3_HUMAN	Collagen alpha-3(VI) chain
32,443	2	412,242	822,470	-0,001	-1,206	51,16	VGLEHLR		CO6A3_HUMAN	Collagen alpha-3(VI) chain
37,001	3	340,176	1017,506	-0,001	-0,931	28,87	LMHLEFGR	[2] Oxidation (M)	CO6A3_HUMAN	Collagen alpha-3(VI) chain
39,854	2	573,808	1145,601	-0,003	-2,381	40,62	SSGIVSLGVGDR		CO6A3_HUMAN	Collagen alpha-3(VI) chain
41,083	2	619,829	1237,644	-0,001	-1,184	47,18	VAVFFSNTPTR		CO6A3_HUMAN	Collagen alpha-3(VI) chain
46,480	3	487,292	1458,853	-0,002	-1,606	25,39	IGDLHPQIVNLLK		CO6A3_HUMAN	Collagen alpha-3(VI) chain
45,376	2	731,885	1461,756	-0,002	-1,234	27,26	QINVGNALEYVSR		CO6A3_HUMAN	Collagen alpha-3(VI) chain

56,800	2	809,479	1616,943	-0,001	-0,422	43,41	AAPLQGMLPGL LAPLR			CO6A3_HUMAN	Collagen alpha-3(VI) chain
51,197	2	817,476	1632,938	-0,001	-0,483	27,98	AAPLQGMLPGL LAPLR	[7] (M)	Oxidation	CO6A3_HUMAN	Collagen alpha-3(VI) chain
44,625	2	489,796	977,578	-0,002	-1,634	29,85	VLTLELYK			KCRM_HUMAN	Creatine kinase M-type
40,987	2	553,844	1105,673	-0,002	-1,462	32,75	VLTLELYKK			KCRM_HUMAN	Creatine kinase M-type
40,959	3	369,566	1105,675	0,000	0,370	27,56	VLTLELYKK			KCRM_HUMAN	Creatine kinase M-type
39,277	2	651,821	1301,627	-0,001	-0,785	33,03	GQSIDDMIPAQK			KCRM_HUMAN	Creatine kinase M-type
32,373	2	659,818	1317,621	-0,003	-2,039	34,89	GQSIDDMIPAQK	[7] (M)	Oxidation	KCRM_HUMAN	Creatine kinase M-type
41,410	2	754,353	1506,692	-0,003	-1,775	58,36	GGDDLDPNYVL SSR			KCRM_HUMAN	Creatine kinase M-type
49,168	2	754,404	1506,792	-0,001	-0,341	83,26	LSVEALNSLTGEF K			KCRM_HUMAN	Creatine kinase M-type
31,077	2	684,360	1366,706	-0,003	-2,367	51,17	SVTTSNPSKFTAK			CSRP3_HUMAN	Cysteine and glycine-rich protein 3
49,131	3	644,713	1931,118	-0,002	-1,201	50,24	LLDVDNRVVLPI EAPIR			COX2_HUMAN	Cytochrome c oxidase subunit 2
34,018	2	453,263	904,512	-0,001	-1,528	28,35	LVPQQLAH			COX5B_HUMAN	Cytochrome c oxidase subunit 5B, mitochondrial
43,885	2	593,826	1185,638	-0,002	-1,355	38,43	GLDPYNVLAPK			COX5B_HUMAN	Cytochrome c oxidase

									subunit 5B, mitochondrial
38,383	2	425,245	848,476	0,000	0,039	48,74	AGIFQSVK	COX6C_HUMAN	Cytochrome c oxidase subunit 6C
35,208	2	489,292	976,569	-0,002	-2,099	35,74	KAGIFQSVK	COX6C_HUMAN	Cytochrome c oxidase subunit 6C
45,065	2	617,829	1233,643	-0,002	-1,486	37,58	QTESLESLLSK	CKAP4_HUMAN	Cytoskeleton-associated protein 4
33,781	3	451,580	1351,719	-0,002	-1,252	25,67	ELHLDNNKLTR	PGS2_HUMAN	Decorin
30,705	2	376,208	750,401	-0,001	-1,235	27,85	VYQVSR	DESM_HUMAN	Desmin
28,299	2	434,211	866,407	-0,003	-2,926	63,65	GSSSSVTSR	DESM_HUMAN	Desmin
32,875	2	501,763	1001,512	-0,003	-2,549	66,3	ADVDAATLAR	DESM_HUMAN	Desmin
35,718	2	516,774	1031,534	-0,002	-2,254	88,47	TSGGAGGLGSLR	DESM_HUMAN	Desmin
49,886	2	825,453	1648,892	-0,001	-0,831	65,92	INLPIQTYSALNF R	DESM_HUMAN	Desmin
31,965	3	577,298	1728,872	-0,003	-1,953	28,64	VSDLTQAANKN NDALR	DESM_HUMAN	Desmin
31,965	2	865,444	1728,873	-0,003	-1,556	31,28	VSDLTQAANKN NDALR	DESM_HUMAN	Desmin
39,117	2	884,921	1767,828	0,000	0,045	32,75	DGEVVSEATQQ QHEVL	DESM_HUMAN	Desmin
53,687	2	1044,54 9	2087,084	0,000	-0,189	96,03	TFGGAPGFPLGS PLSSPVFPR	DESM_HUMAN	Desmin
53,708	3	696,702	2087,084	0,000	0,102	51,57	TFGGAPGFPLGS PLSSPVFPR	DESM_HUMAN	Desmin
50,251	3	748,735	2243,184	-0,001	-0,540	71,54	RTFGGAPGFPLG	DESM_HUMAN	Desmin

							SPLSSPVFPR			
45,166	2	769,943	1537,871	0,000	-0,077	27,3	LLEAQIATGGIID PK		DESP_HUMAN	Desmoplakin
33,642	2	409,208	816,401	0,004	4,307	26,25	DQQIGEK		MCM2_HUMAN	DNA replication licensing factor MCM2
37,463	2	427,265	852,516	-0,002	-2,356	55,31	LGPLQVAR		ETFB_HUMAN	Electron transfer flavoprotein subunit beta
39,912	2	670,364	1338,713	-0,001	-0,745	51,07	LSVISVEDPPQR		ETFB_HUMAN	Electron transfer flavoprotein subunit beta
38,468	2	513,308	1024,601	-0,002	-1,595	53,41	IGGIGTVPVGR		EF1A1_HUMAN	Elongation factor 1-alpha 1
38,468	2	513,308	1024,601	-0,002	-1,595	53,41	IGGIGTVPVGR		EF1A2_HUMAN	Elongation factor 1-alpha 2
51,385	3	857,812	2570,414	-0,001	-0,269	28,39	VETGILRPGMVV TFAPVNITTEVK		EF1A2_HUMAN	Elongation factor 1-alpha 2
49,194	3	863,143	2586,408	-0,001	-0,257	36,97	VETGILRPGMVV TFAPVNITTEVK	[10] Oxidation (M)	EF1A2_HUMAN	Elongation factor 1-alpha 2
40,301	2	546,295	1090,575	-0,002	-1,888	38,58	VNFTVDQIR		EF2_HUMAN	Elongation factor 2
48,806	2	567,828	1133,642	-0,002	-1,906	42,48	SLNILTAFQK		ERP29_HUMAN	Endoplasmic reticulum resident protein 29
44,253	3	784,423	2350,248	-0,003	-1,158	29,85	TVATPLNQVAN PNSAIFGGARPR		IF4H_HUMAN	Eukaryotic translation initiation factor 4H
44,790	2	723,401	1444,788	-0,001	-0,597	78,09	QLLTLSSELSQAR		EZRI_HUMAN	Ezrin

50,709	4	592,828	2367,283	-0,002	-0,824	28,85	ITHYQLNFQTGL LVPAHIFR	FBLN2_HUMAN	Fibulin-2
36,910	3	521,611	1561,810	-0,004	-2,276	30,26	PYQYPALTPEQK K	ALDOA_HUMAN	Fructose-bisphosphate aldolase A
43,310	2	1114,51 6	2227,017	-0,001	-0,320	63,4	YTPSGQAGAAAS ESLFVSNHAY	ALDOA_HUMAN	Fructose-bisphosphate aldolase A
33,350	2	544,771	1087,527	0,005	4,880	25,24	CTIEEAPPTK	CCNB3_HUMAN	G2/mitotic-specific cyclin-B3
38,571	3	425,207	1272,598	-0,002	-1,320	26,25	GNDVAFHFNPR	LEG3_HUMAN	Galectin-3
46,003	3	547,655	1639,942	-0,002	-1,294	25,73	MLITILGTVKPN ANR	LEG3_HUMAN	Galectin-3
45,625	3	680,024	2037,051	-0,002	-1,142	32,53	VTGPEGALFEHS VETPLVR	SGCG_HUMAN	Gamma-sarcoglycan
35,413	2	378,237	754,459	0,000	-0,400	25,03	AVEVLPK	GELS_HUMAN	Gelsolin
33,443	2	403,219	804,424	-0,001	-0,811	43,55	VGVNGFGR	G3P_HUMAN	Glyceraldehyde-3-phosphate dehydrogenase
33,126	2	403,219	804,424	0,000	-0,612	46,66	VGVNGFGR	G3P_HUMAN	Glyceraldehyde-3-phosphate dehydrogenase
30,105	2	533,298	1064,582	-0,002	-1,934	31,49	AGAHLQGGAKR	G3P_HUMAN	Glyceraldehyde-3-phosphate dehydrogenase
41,934	2	706,398	1410,782	-0,002	-1,086	58,42	GALQNIIPASTG AAK	G3P_HUMAN	Glyceraldehyde-3-phosphate dehydrogenase

58,858	4	649,595	2594,351	-0,002	-0,683	55,38	VIHDNFGIVEGL MTTVHAITATQK			G3P_HUMAN	Glyceraldehyde-3-phosphate dehydrogenase
58,874	3	865,791	2594,352	-0,001	-0,450	71,86	VIHDNFGIVEGL MTTVHAITATQK			G3P_HUMAN	Glyceraldehyde-3-phosphate dehydrogenase
51,785	4	653,594	2610,346	-0,002	-0,631	28,79	VIHDNFGIVEGL MTTVHAITATQK	[13] (M)	Oxidation	G3P_HUMAN	Glyceraldehyde-3-phosphate dehydrogenase
52,008	4	653,594	2610,346	-0,002	-0,632	45,86	VIHDNFGIVEGL MTTVHAITATQK	[13] (M)	Oxidation	G3P_HUMAN	Glyceraldehyde-3-phosphate dehydrogenase
52,008	3	871,123	2610,347	-0,001	-0,420	67,76	VIHDNFGIVEGL MTTVHAITATQK	[13] (M)	Oxidation	G3P_HUMAN	Glyceraldehyde-3-phosphate dehydrogenase
51,806	3	871,123	2610,347	-0,001	-0,286	48,8	VIHDNFGIVEGL MTTVHAITATQK	[13] (M)	Oxidation	G3P_HUMAN	Glyceraldehyde-3-phosphate dehydrogenase
30,873	2	502,273	1002,531	-0,004	-3,538	37,79	LSKEEIER			HSP71_HUMAN	Heat shock 70 kDa protein 1A/1B
32,339	2	614,816	1227,618	-0,003	-2,326	26,78	VEIIANDQGNR			HSP71_HUMAN	Heat shock 70 kDa protein 1A/1B
33,302	2	616,823	1231,632	-0,002	-1,752	31,53	AMTKDNNLLGR			HSP71_HUMAN	Heat shock 70 kDa protein 1A/1B
39,159	2	652,303	1302,591	0,000	-0,256	55,78	NALESYAFNMK	[10] (M)	Oxidation	HSP71_HUMAN	Heat shock 70 kDa protein 1A/1B

39,535	2	744,354	1486,694	0,000	-0,084	46,55	TTPSYVAFTDTER	HSP71_HUMAN	Heat shock 70 kDa protein 1A/1B
39,194	3	605,655	1813,942	-0,002	-0,919	27,03	NQVALNPQNTV FDAKR	HSP71_HUMAN	Heat shock 70 kDa protein 1A/1B
32,339	2	614,816	1227,618	-0,003	-2,326	26,78	VEILANDQGNR	HSP76_HUMAN	Heat shock 70 kDa protein 6
33,302	2	616,823	1231,632	-0,002	-1,752	31,53	AMTKDNNLLGR	HSP76_HUMAN	Heat shock 70 kDa protein 6
39,535	2	744,354	1486,694	0,000	-0,084	46,55	TTPSYVAFTDTER	HSP76_HUMAN	Heat shock 70 kDa protein 6
32,339	2	614,816	1227,618	-0,003	-2,326	26,78	VEIIANDQGNR	HSP7C_HUMAN	Heat shock cognate 71 kDa protein
46,480	2	627,311	1252,608	-0,001	-0,551	31,67	FEELNADLFR	HSP7C_HUMAN	Heat shock cognate 71 kDa protein
39,535	2	744,354	1486,694	0,000	-0,084	46,55	TTPSYVAFTDTER	HSP7C_HUMAN	Heat shock cognate 71 kDa protein
42,114	2	494,308	986,600	-0,002	-2,053	31,35	RVPFSLLR	HSPB1_HUMAN	Heat shock protein beta-1
34,670	2	538,290	1074,565	-0,002	-2,024	66,71	QLSSGVSEIR	HSPB1_HUMAN	Heat shock protein beta-1
34,314	2	538,290	1074,565	-0,002	-1,798	35,47	QLSSGVSEIR	HSPB1_HUMAN	Heat shock protein beta-1
46,568	2	582,313	1162,612	-0,001	-1,182	51,02	LFDQAFGLPR	HSPB1_HUMAN	Heat shock protein beta-1
46,350	2	953,499	1904,984	-0,001	-0,306	39,76	LATQSNEITIPVT FESR	HSPB1_HUMAN	Heat shock protein beta-1

47,702	3	1076,231	3225,671	0,023	7,060	26,02	KYTLPPGVDPTQVSSSLSPEGTLTVEAPMPK		HSPB1_HUMAN	Heat shock protein beta-1
46,584	3	1081,554	3241,642	-0,001	-0,391	31,36	KYTLPPGVDPTQVSSSLSPEGTLTVEAPMPK	[29] Oxidation (M)	HSPB1_HUMAN	Heat shock protein beta-1
51,463	3	1279,691	3836,050	0,003	0,659	46,57	YRLPPGVDPAAVTSALSPEGVLSIQAAPASAQAPPPAAAK		HSPB6_HUMAN	Heat shock protein beta-6
32,339	2	614,816	1227,618	-0,003	-2,326	26,78	VEIIANDQGNR		HSP72_HUMAN	Heat shock-related 70 kDa protein 2
46,480	2	627,311	1252,608	-0,001	-0,551	31,67	FEELNADLFR		HSP72_HUMAN	Heat shock-related 70 kDa protein 2
39,535	2	744,354	1486,694	0,000	-0,084	46,55	TTPSYVAFTDTER		HSP72_HUMAN	Heat shock-related 70 kDa protein 2
46,243	2	536,280	1070,545	-0,002	-1,744	32,39	MFLSFPTTK		HBA_HUMAN	Hemoglobin subunit alpha
33,317	3	510,582	1528,723	-0,004	-2,410	65,55	VGAHAGEYGAEALER		HBA_HUMAN	Hemoglobin subunit alpha
33,317	2	765,370	1528,725	-0,002	-0,982	45,35	VGAHAGEYGAEALER		HBA_HUMAN	Hemoglobin subunit alpha
34,775	2	814,893	1627,772	-0,002	-1,413	58,18	SSGPYGGGGQYFAKPR		ROA1_HUMAN	Heterogeneous nuclear ribonucleoprotein A1
36,511	2	507,224	1012,434	-0,003	-2,595	28,87	GGNFGFGDSR		ROA2_HUMAN	Heterogeneous

											nuclear ribonucleoproteins A2/B1
33,797	2	1095,456	2188,898	-0,001	-0,241	43,72	NMGGPYGGGNYGPGGSGGSGGYGGR			ROA2_HUMAN	Heterogeneous nuclear ribonucleoproteins A2/B1
33,866	2	472,289	942,563	-0,002	-2,608	36,36	VPPPPPIAR			HNRPC_HUMAN	Heterogeneous nuclear ribonucleoproteins C1/C2
41,271	2	727,862	1453,709	-0,003	-2,024	29,74	YSDMIVAAIQAEK	[4] (M)	Oxidation	H10_HUMAN	Histone H1.0
36,977	2	487,305	972,595	-0,002	-2,041	45,36	SGVSLAALKK			H13_HUMAN	Histone H1.3
34,505	2	554,286	1106,558	-0,002	-2,142	44,01	ALAAAGYDVEK			H13_HUMAN	Histone H1.3
34,175	2	554,287	1106,559	-0,002	-2,033	47,87	ALAAAGYDVEK			H13_HUMAN	Histone H1.3
39,888	2	599,837	1197,659	-0,001	-0,959	64,14	ASGPPVSELITK			H13_HUMAN	Histone H1.3
34,505	2	630,879	1259,743	-0,002	-1,752	47,69	SLVSKGTLVQTK			H13_HUMAN	Histone H1.3
37,841	2	663,883	1325,752	-0,004	-2,763	42,44	KASGPPVSELITK			H13_HUMAN	Histone H1.3
34,505	2	630,879	1259,743	-0,002	-1,752	47,69	SLVSKGTLVQTK			H15_HUMAN	Histone H1.5
34,505	2	554,286	1106,558	-0,002	-2,142	44,01	ALAAAGYDVEK			H1T_HUMAN	Histone H1t
34,175	2	554,287	1106,559	-0,002	-2,033	47,87	ALAAAGYDVEK			H1T_HUMAN	Histone H1t
42,568	2	671,390	1340,765	-0,001	-1,041	27,41	ALVQNDTLLQVK			H1X_HUMAN	Histone H1x
41,083	2	472,769	943,524	0,000	-0,467	61,97	AGLQFPVGR			H2A2A_HUMAN	Histone H2A type 2-A
55,118	3	644,394	1930,160	-0,002	-1,009	52,33	VTIAQGGVLPNI			H2A2A_HUMAN	Histone H2A type 2-A

55,118	2	966,087	1930,160	-0,001	-0,636	86,75	QAVLLPK VTIAQGGVLPNI QAVLLPK		H2A2A_HUMAN	Histone H2A type 2-A
28,014	2	414,713	827,412	-0,002	-2,566	35,25	HAVSEGTK		H2B1B_HUMAN	Histone H2B type 1-B
43,438	2	477,305	952,595	0,000	-0,386	49,38	LLLPGELAK		H2B1B_HUMAN	Histone H2B type 1-B
56,898	2	872,413	1742,811	-0,001	-0,479	88,66	AMGIMNSFVNDI FER		H2B1B_HUMAN	Histone H2B type 1-B
54,061	2	880,410	1758,806	-0,001	-0,331	104,62	AMGIMNSFVNDI FER	[2] Oxidation (M)	H2B1B_HUMAN	Histone H2B type 1-B
51,073	2	880,411	1758,806	0,000	-0,254	60,13	AMGIMNSFVNDI FER	[5] Oxidation (M)	H2B1B_HUMAN	Histone H2B type 1-B
49,131	2	888,408	1774,801	-0,001	-0,337	48,88	AMGIMNSFVNDI FER	[2] Oxidation (M) | [5] Oxidation (M)	H2B1B_HUMAN	Histone H2B type 1-B
34,053	2	344,706	687,398	-0,001	-1,054	35,49	VTIMPK		H31T_HUMAN	Histone H3.1t
29,220	2	344,721	687,427	-0,001	-1,467	27,64	KQLATK		H31T_HUMAN	Histone H3.1t
38,962	2	416,250	830,486	0,000	0,203	29,06	STELLIR		H31T_HUMAN	Histone H3.1t
32,339	3	344,870	1031,587	0,000	-0,248	28,91	YRPGTVALR		H31T_HUMAN	Histone H3.1t
37,034	2	663,380	1324,745	-0,001	-0,870	45,61	DNIQGITKPAIR		H4_HUMAN	Histone H4
46,279	2	733,905	1465,795	-0,002	-1,202	61,03	TVTAMDVVYAL KR		H4_HUMAN	Histone H4
33,642	3	494,622	1480,844	-0,004	-2,386	29,78	DNIQGITKPAIRR		H4_HUMAN	Histone H4
42,398	3	494,937	1481,789	-0,003	-1,789	30,06	TVTAMDVVYAL KR	[5] Oxidation (M)	H4_HUMAN	Histone H4
33,217	2	426,221	850,428	0,006	7,566	26,56	DTLMISR	[4] Oxidation (M)	IGHG1_HUMAN	Ig gamma-1 chain C region

42,035	2	593,826	1185,637	-0,002	-1,613	39,48	GPSVFPLAPSSK		IGHG1_HUMAN	Ig gamma-1 chain C region
56,442	2	855,911	1709,808	0,003	1,994	25,14	FEAAETLEEAAM RSR		KLC1_HUMAN	Kinesin light chain 1
39,805	3	794,722	2381,144	-0,002	-0,970	40,67	VAPQQDDLDSP QQISISNAEAR		LAMA2_HUMAN	Laminin subunit alpha-2
49,431	2	607,295	1212,576	-0,001	-1,129	33,46	DGFFGLSISDR		LAMB2_HUMAN	Laminin subunit beta-2
31,485	2	924,941	1847,867	0,006	3,493	52,86	AGNSLAASTAEE TAGSAQGR		LAMB2_HUMAN	Laminin subunit beta-2
33,698	2	816,397	1630,779	0,000	-0,243	27,26	EAQQALGSAAA DATEAK		LAMC1_HUMAN	Laminin subunit gamma-1
42,583	2	662,858	1323,702	-0,001	-0,964	33,76	SASYNLSLTLQK		LDB3_HUMAN	LIM domain-binding protein 3
40,570	2	824,943	1647,872	-0,001	-0,653	61,58	GGPAYTPAGPQ VPPLAR		LDB3_HUMAN	LIM domain-binding protein 3
51,660	3	970,537	2908,590	-0,001	-0,360	27,86	ASGVGLPGGSLPI KDLAVDSASPVY QAVIK		LDB3_HUMAN	LIM domain-binding protein 3
37,306	2	457,294	912,574	-0,001	-1,456	38,43	IVVVTAGVR		LDHB_HUMAN	L-lactate dehydrogenase B chain
45,506	2	512,781	1023,548	-0,002	-1,851	26,88	FNALQYLR		LUM_HUMAN	Lumican
40,356	2	652,343	1302,672	-0,003	-2,474	41,21	PMFIVNTNVPR	[2] Oxidation (M)	MIF_HUMAN	Macrophage migration inhibitory factor

32,270	2	322,210	642,406	0,000	-0,131	33,2	AQIALK	MDHC_HUMAN	Malate dehydrogenase, cytoplasmic
34,018	2	582,803	1163,591	-0,003	-2,189	35,89	GEFVTTVQQR	MDHC_HUMAN	Malate dehydrogenase, cytoplasmic
33,698	2	582,803	1163,591	-0,002	-1,949	28,03	GEFVTTVQQR	MDHC_HUMAN	Malate dehydrogenase, cytoplasmic
56,769	2	897,048	1792,081	-0,002	-0,934	40,47	VAVLGASGGIGQ PLSLLLK	MDHM_HUMAN	Malate dehydrogenase, mitochondrial
49,007	3	789,084	2364,231	-0,001	-0,573	31,54	LTLYDIAHTPGV AADLSHIETK	MDHM_HUMAN	Malate dehydrogenase, mitochondrial
44,094	2	530,774	1059,533	-0,002	-1,560	34,27	DFADIPNLR	MIME_HUMAN	Mimecan
64,653	4	507,747	2026,959	0,011	5,532	32,96	TETTMSPLTNTT TSQGTTR	MUC5B_HUMAN	Mucin-5B
42,771	2	374,721	747,428	0,000	0,026	40,82	ALELFR	MYG_HUMAN	Myoglobin
38,874	2	438,769	875,523	0,000	-0,435	44,21	ALELFRK	MYG_HUMAN	Myoglobin
49,528	3	450,941	1349,802	-0,001	-1,019	49,4	HGATVLTALGGI LK	MYG_HUMAN	Myoglobin
49,528	2	675,908	1349,802	-0,001	-0,537	86,21	HGATVLTALGGI LK	MYG_HUMAN	Myoglobin
46,685	3	493,639	1477,896	-0,002	-1,621	70,53	HGATVLTALGGI LKK	MYG_HUMAN	Myoglobin

38,502	2	698,880	1395,746	-0,001	-0,715	32,13	ALGQNPTQAEV LR			MYL3_HUMAN	Myosin light chain 3
32,855	2	484,281	966,548	-0,002	-2,157	45,45	HVLATLGEK			MYL4_HUMAN	Myosin light chain 4
32,511	2	484,281	966,548	-0,002	-2,153	35,83	HVLATLGEK			MYL4_HUMAN	Myosin light chain 4
36,814	2	632,303	1262,592	0,000	-0,335	47,85	ESNGTVMGAEL R			MYL4_HUMAN	Myosin light chain 4
37,306	2	691,872	1381,730	-0,001	-0,921	63,56	ALGQNPTNAEV LR			MYL4_HUMAN	Myosin light chain 4
63,526	3	654,016	1959,027	-0,002	-0,943	72,2	MLDFETFLPILQ HISR			MYL4_HUMAN	Myosin light chain 4
63,526	2	980,521	1959,027	-0,002	-0,903	72,34	MLDFETFLPILQ HISR			MYL4_HUMAN	Myosin light chain 4
60,449	3	659,348	1975,022	-0,002	-0,909	66,89	MLDFETFLPILQ HISR	[1] (M)	Oxidation	MYL4_HUMAN	Myosin light chain 4
60,473	2	988,518	1975,022	-0,002	-0,796	84,67	MLDFETFLPILQ HISR	[1] (M)	Oxidation	MYL4_HUMAN	Myosin light chain 4
31,485	4	604,085	2412,310	-0,006	-2,577	29,4	KEAAKPAPAPAP APAPAPAPAPEA PK			MYL4_HUMAN	Myosin light chain 4
31,485	3	805,112	2412,313	-0,004	-1,540	31,39	KEAAKPAPAPAP APAPAPAPAPEA PK			MYL4_HUMAN	Myosin light chain 4
57,969	3	630,006	1886,995	-0,002	-0,830	33,26	VLDFEHFLPMLQ TVAK			MYL6_HUMAN	Myosin light polypeptide 6
53,435	3	635,337	1902,989	-0,003	-1,422	29,4	VLDFEHFLPMLQ TVAK	[10] (M)	Oxidation	MYL6_HUMAN	Myosin light polypeptide 6

33,078	2	530,761	1059,507	-0,002	-2,099	50,2	ANSEVAQWR			MYH13_HUMAN	Myosin-13
56,261	2	601,333	1200,652	-0,001	-1,241	49,29	AGLLGLLEEMR			MYH13_HUMAN	Myosin-13
49,754	2	609,331	1216,647	-0,002	-1,419	52,9	AGLLGLLEEMR	[10] (M)	Oxidation	MYH13_HUMAN	Myosin-13
34,209	2	672,366	1342,718	-0,003	-1,942	57,16	ADIAESQVNKLR			MYH13_HUMAN	Myosin-13
34,209	3	448,580	1342,719	-0,002	-1,216	31,1	ADIAESQVNKLR			MYH13_HUMAN	Myosin-13
33,585	3	448,580	1342,719	-0,002	-1,123	29,21	ADIAESQVNKLR			MYH13_HUMAN	Myosin-13
33,585	2	672,367	1342,719	-0,002	-1,121	64,73	ADIAESQVNKLR			MYH13_HUMAN	Myosin-13
56,261	2	601,333	1200,652	-0,001	-1,241	49,29	AGLLGLLEEMR			MYH2_HUMAN	Myosin-2
49,754	2	609,331	1216,647	-0,002	-1,419	52,9	AGLLGLLEEMR	[10] (M)	Oxidation	MYH2_HUMAN	Myosin-2
34,209	2	672,366	1342,718	-0,003	-1,942	57,16	ADIAESQVNKLR			MYH2_HUMAN	Myosin-2
34,209	3	448,580	1342,719	-0,002	-1,216	31,1	ADIAESQVNKLR			MYH2_HUMAN	Myosin-2
33,585	3	448,580	1342,719	-0,002	-1,123	29,21	ADIAESQVNKLR			MYH2_HUMAN	Myosin-2
33,585	2	672,367	1342,719	-0,002	-1,121	64,73	ADIAESQVNKLR			MYH2_HUMAN	Myosin-2
33,078	2	530,761	1059,507	-0,002	-2,099	50,2	ANSEVAQWR			MYH3_HUMAN	Myosin-3
34,209	2	672,366	1342,718	-0,003	-1,942	57,16	ADIAESQVNKLR			MYH3_HUMAN	Myosin-3
34,209	3	448,580	1342,719	-0,002	-1,216	31,1	ADIAESQVNKLR			MYH3_HUMAN	Myosin-3
33,585	3	448,580	1342,719	-0,002	-1,123	29,21	ADIAESQVNKLR			MYH3_HUMAN	Myosin-3
33,585	2	672,367	1342,719	-0,002	-1,121	64,73	ADIAESQVNKLR			MYH3_HUMAN	Myosin-3
39,159	2	442,237	882,460	0,000	-0,395	30,66	ILYGDFR			MYH6_HUMAN	Myosin-6
33,078	2	530,761	1059,507	-0,002	-2,099	50,2	ANSEVAQWR			MYH6_HUMAN	Myosin-6
35,149	2	541,266	1080,518	-0,002	-1,894	50,48	SLNDFTTQR			MYH6_HUMAN	Myosin-6
56,261	2	601,333	1200,652	-0,001	-1,241	49,29	AGLLGLLEEMR			MYH6_HUMAN	Myosin-6
49,754	2	609,331	1216,647	-0,002	-1,419	52,9	AGLLGLLEEMR	[10] (M)	Oxidation	MYH6_HUMAN	Myosin-6

43,438	3	438,266	1311,775	-0,001	-0,995	25,28	LQDLVDKLQLK		MYH6_HUMAN	Myosin-6
34,209	2	672,366	1342,718	-0,003	-1,942	57,16	ADIAESQVNKLR		MYH6_HUMAN	Myosin-6
34,209	3	448,580	1342,719	-0,002	-1,216	31,1	ADIAESQVNKLR		MYH6_HUMAN	Myosin-6
33,585	3	448,580	1342,719	-0,002	-1,123	29,21	ADIAESQVNKLR		MYH6_HUMAN	Myosin-6
33,585	2	672,367	1342,719	-0,002	-1,121	64,73	ADIAESQVNKLR		MYH6_HUMAN	Myosin-6
49,886	2	721,385	1440,756	-0,001	-0,705	53,96	NNLLQAELEELR		MYH6_HUMAN	Myosin-6
48,770	2	762,414	1522,814	-0,001	-0,441	99,32	VIQYFASIAAIGDR		MYH6_HUMAN	Myosin-6
39,677	2	767,391	1532,768	0,000	-0,215	82,04	VVDSLQTSLDAETR		MYH6_HUMAN	Myosin-6
50,683	2	874,442	1746,870	-0,001	-0,498	35,04	LELDDVTSNMEQIIK		MYH6_HUMAN	Myosin-6
47,816	2	884,983	1767,952	0,000	-0,095	66,66	ILNPVAIPEGQFIDSR		MYH6_HUMAN	Myosin-6
40,902	3	602,654	1804,942	-0,003	-1,385	30,09	KAPGVMDNPLVMHQLR		MYH6_HUMAN	Myosin-6
41,813	2	919,965	1837,916	-0,001	-0,533	87,12	DLEEATLQHEATAAALR		MYH6_HUMAN	Myosin-6
41,813	3	613,646	1837,917	0,000	-0,112	35,38	DLEEATLQHEATAAALR		MYH6_HUMAN	Myosin-6
37,098	3	617,685	1850,034	-0,003	-1,769	48,84	VQLLHSQNTSLINQKK		MYH6_HUMAN	Myosin-6
45,625	3	633,022	1896,044	-0,003	-1,557	36,48	ILNPVAIPEGQFIDSRK		MYH6_HUMAN	Myosin-6
39,805	3	656,344	1966,010	-0,002	-0,869	30,33	DLEEATLQHEATAAALRK		MYH6_HUMAN	Myosin-6

42,971	3	662,971	1985,890	-0,003	-1,550	41,6	MEGDLNEMEIQLSHANR			MYH6_HUMAN	Myosin-6
47,739	3	666,001	1994,982	-0,003	-1,361	37,82	HRLQNEIEDLMVDVER			MYH6_HUMAN	Myosin-6
43,885	3	696,354	2086,041	-0,002	-1,060	38,45	VKLEQQVDDLEGSLEQEK			MYH6_HUMAN	Myosin-6
47,367	3	696,713	2087,116	-0,001	-0,287	33,09	YRILNPVAIPEGQFIDSR			MYH6_HUMAN	Myosin-6
50,725	2	1100,565	2199,116	-0,001	-0,316	49,68	GTLEDQIIQANPALEAFGNAK			MYH6_HUMAN	Myosin-6
49,272	3	976,488	2926,441	-0,001	-0,419	43,19	DNANANKGTLEDQIIQANPALEAFGNAK			MYH6_HUMAN	Myosin-6
39,159	2	442,237	882,460	0,000	-0,395	30,66	ILYGDFR			MYH7_HUMAN	Myosin-7
33,078	2	530,761	1059,507	-0,002	-2,099	50,2	ANSEVAQWR			MYH7_HUMAN	Myosin-7
56,261	2	601,333	1200,652	-0,001	-1,241	49,29	AGLLGLLEEMR			MYH7_HUMAN	Myosin-7
49,754	2	609,331	1216,647	-0,002	-1,419	52,9	AGLLGLLEEMR	[10] (M)	Oxidation	MYH7_HUMAN	Myosin-7
43,438	3	438,266	1311,775	-0,001	-0,995	25,28	LQDLVDKLQLK			MYH7_HUMAN	Myosin-7
34,209	2	672,366	1342,718	-0,003	-1,942	57,16	ADIAESQVNKLR			MYH7_HUMAN	Myosin-7
34,209	3	448,580	1342,719	-0,002	-1,216	31,1	ADIAESQVNKLR			MYH7_HUMAN	Myosin-7
33,585	3	448,580	1342,719	-0,002	-1,123	29,21	ADIAESQVNKLR			MYH7_HUMAN	Myosin-7
33,585	2	672,367	1342,719	-0,002	-1,121	64,73	ADIAESQVNKLR			MYH7_HUMAN	Myosin-7
49,886	2	721,385	1440,756	-0,001	-0,705	53,96	NNLLQAELEELR			MYH7_HUMAN	Myosin-7
39,677	2	767,391	1532,768	0,000	-0,215	82,04	VVDSLQTSLDAETR			MYH7_HUMAN	Myosin-7

50,683	2	874,442	1746,870	-0,001	-0,498	35,04	LELDDVTSNMEQIIK		MYH7_HUMAN	Myosin-7
41,813	2	919,965	1837,916	-0,001	-0,533	87,12	DLEEATLQHEATAAALR		MYH7_HUMAN	Myosin-7
41,813	3	613,646	1837,917	0,000	-0,112	35,38	DLEEATLQHEATAAALR		MYH7_HUMAN	Myosin-7
37,098	3	617,685	1850,034	-0,003	-1,769	48,84	VQLLHSQNTSLINQKK		MYH7_HUMAN	Myosin-7
39,805	3	656,344	1966,010	-0,002	-0,869	30,33	DLEEATLQHEATAAALRK		MYH7_HUMAN	Myosin-7
42,971	3	662,971	1985,890	-0,003	-1,550	41,6	MEGDLNEMEIQLSHANR		MYH7_HUMAN	Myosin-7
47,739	3	666,001	1994,982	-0,003	-1,361	37,82	HRLQNEIEDLMVDVER		MYH7_HUMAN	Myosin-7
43,885	3	696,354	2086,041	-0,002	-1,060	38,45	VKLEQQVDDLEGSLEQEK		MYH7_HUMAN	Myosin-7
50,725	2	1100,565	2199,116	-0,001	-0,316	49,68	GTLEDQIIQANPALEAFGNAK		MYH7_HUMAN	Myosin-7
34,018	2	450,779	899,543	-0,001	-1,392	29,17	VQLEGKVK		MYH7B_HUMAN	Myosin-7B
41,410	2	924,952	1847,889	-0,001	-0,550	37,01	SVEVAAGSPAVFEAETER		MYPC3_HUMAN	Myosin-binding protein C, cardiac-type
43,670	2	786,887	1571,760	-0,002	-1,143	49,39	DIMLEELSHLSNR	[3] Oxidation (M)	MYOZ2_HUMAN	Myozenin-2
39,805	2	862,436	1722,857	-0,001	-0,474	47,13	SPPNPDNIAPGYSGPLK		MYOZ2_HUMAN	Myozenin-2

43,710	2	648,387	1294,759	-0,002	-1,427	30,84	VALSPAGVQNL VK	NNTM_HUMAN	NAD(P) transhydrogenase, mitochondrial
35,868	2	371,235	740,455	0,001	0,780	40,11	ALLTAPR	ANF_HUMAN	Natriuretic peptides A
37,560	2	505,826	1009,637	-0,003	-2,741	28,74	LRALLTAPR	ANF_HUMAN	Natriuretic peptides A
35,379	2	680,340	1358,666	-0,002	-1,543	25,52	LLEEENQESLR	NEST_HUMAN	Nestin
33,350	2	544,771	1087,527	-0,010	-9,004	25,3	NAQKSNQNGK	NPM_HUMAN	Nucleophosmin
55,110	4	958,496	3829,954	-0,001	-0,376	33,78	AENPLGAASAA AALVVDSDAAD TASRPGTSTAAL LAHLQR	OBSCN_HUMAN	Obscurin
29,644	3	365,860	1094,557	0,014	12,541	25,48	KITIADCGQF	PAL4A_HUMAN	Peptidyl-prolyl cis-trans isomerase A-like 4A/B/C
44,869	3	726,402	2176,184	0,006	2,905	35,38	TVLTGTKDTVTT GVMGAVNLAK	PLIN4_HUMAN	Perilipin-4
39,888	2	655,306	1308,597	-0,001	-1,082	49,06	NLQEAEEWYK	PERI_HUMAN	Peripherin
39,025	2	554,305	1106,595	-0,002	-1,999	25,88	TIAQDYGVLK	PRDX1_HUMAN	Peroxiredoxin-1
45,315	3	593,657	1777,950	-0,001	-0,726	31,58	QGGLGPMNIPLV SDPKR	PRDX1_HUMAN	Peroxiredoxin-1
49,886	2	549,313	1096,611	-0,002	-1,553	51,55	VLPGVDALSNI	PGK1_HUMAN	Phosphoglycerate kinase 1
40,491	3	545,601	1633,781	-0,004	-2,366	31,93	LGDVYVNDAFG TAHR	PGK1_HUMAN	Phosphoglycerate kinase 1
30,705	2	488,248	974,481	-0,004	-4,203	36,63	AMEAVAAQGK	PGAM2_HUMAN	Phosphoglycerate mutase 2

29,781	2	560,293	1118,572	-0,003	-2,826	41,66	KAMEAVAAQGK	[3] Oxidation (M)	PGAM2_HUMAN	Phosphoglycerate mutase 2
34,480	2	575,837	1149,659	-0,003	-2,259	53,36	VLIAAHGNSLR		PGAM2_HUMAN	Phosphoglycerate mutase 2
34,053	2	575,837	1149,660	-0,002	-1,864	45,55	VLIAAHGNSLR		PGAM2_HUMAN	Phosphoglycerate mutase 2
46,350	2	893,951	1785,887	-0,010	-5,625	30,64	GQGDESERIVIN VGGTR		KCNC1_HUMAN	Potassium voltage-gated channel subfamily C member 1
31,246	2	488,727	975,440	-0,001	-1,518	88,56	AGFAGDDAPR		POTEE_HUMAN	POTE ankyrin domain family member E
40,077	2	599,855	1197,696	-0,002	-2,086	41,77	AVFPSIVGRPR		POTEE_HUMAN	POTE ankyrin domain family member E
40,077	3	400,241	1197,701	0,003	2,297	33,51	AVFPSIVGRPR		POTEE_HUMAN	POTE ankyrin domain family member E
45,436	2	895,949	1789,884	-0,001	-0,339	122,91	SYELPDGQVITIG NER		POTEE_HUMAN	POTE ankyrin domain family member E
44,400	2	895,949	1789,884	-0,001	-0,313	28,06	SYELPDGQVITIG NER		POTEE_HUMAN	POTE ankyrin domain family member E
43,392	3	656,697	1967,070	-0,002	-1,235	25,89	VAPEEHPILLTEA PLNPK		POTEE_HUMAN	POTE ankyrin domain family member E
43,392	3	719,382	2155,125	-0,004	-1,644	30,05	AGFAGDDAPRA VFPSIVGRPR		POTEE_HUMAN	POTE ankyrin domain family member E
31,246	2	488,727	975,440	-0,001	-1,518	88,56	AGFAGDDAPR		POTEF_HUMAN	POTE ankyrin domain family member F
40,077	2	599,855	1197,696	-0,002	-2,086	41,77	AVFPSIVGRPR		POTEF_HUMAN	POTE ankyrin domain

									family member F
40,077	3	400,241	1197,701	0,003	2,297	33,51	AVFPSIVGRPR	POTEF_HUMAN	POTE ankyrin domain family member F
45,436	2	895,949	1789,884	-0,001	-0,339	122,91	SYELPDGQVITIG NER	POTEF_HUMAN	POTE ankyrin domain family member F
44,400	2	895,949	1789,884	-0,001	-0,313	28,06	SYELPDGQVITIG NER	POTEF_HUMAN	POTE ankyrin domain family member F
43,392	3	719,382	2155,125	-0,004	-1,644	30,05	AGFAGDDAPRA VFPSIVGRPR	POTEF_HUMAN	POTE ankyrin domain family member F
31,246	2	488,727	975,440	-0,001	-1,518	88,56	AGFAGDDAPR	POTEI_HUMAN	POTE ankyrin domain family member I
40,077	2	599,855	1197,696	-0,002	-2,086	41,77	AVFPSIVGRPR	POTEI_HUMAN	POTE ankyrin domain family member I
40,077	3	400,241	1197,701	0,003	2,297	33,51	AVFPSIVGRPR	POTEI_HUMAN	POTE ankyrin domain family member I
43,392	3	656,697	1967,070	-0,002	-1,235	25,89	VAPEEHPILLTEA PLNPK	POTEI_HUMAN	POTE ankyrin domain family member I
43,392	3	719,382	2155,125	-0,004	-1,644	30,05	AGFAGDDAPRA VFPSIVGRPR	POTEI_HUMAN	POTE ankyrin domain family member I
31,246	2	488,727	975,440	-0,001	-1,518	88,56	AGFAGDDAPR	POTEJ_HUMAN	POTE ankyrin domain family member J
43,392	3	656,697	1967,070	-0,002	-1,235	25,89	VAPEEHPILLTEA PLNPK	POTEJ_HUMAN	POTE ankyrin domain family member J
38,823	2	783,879	1565,743	-0,001	-0,569	43,84	SVGGSGGGSFGD NLVTR	LMNA_HUMAN	Prelamin-A/C
48,770	2	532,797	1063,580	-0,002	-1,829	34,4	SFPNLAFIR	PRELP_HUMAN	Prolargin

44,464	2	783,432	1564,849	-0,002	-1,272	29,41	NLMQLNLAHNILR	[3] Oxidation (M)	PRELP_HUMAN	Prolargin
43,392	2	511,268	1020,522	-0,002	-1,822	56,34	ETLLQDFR		AMBP_HUMAN	Protein AMBP
39,470	2	890,920	1779,825	-0,003	-1,405	51,42	VDATEESDLAQQYGVR		PDIA1_HUMAN	Protein disulfide-isomerase
42,087	2	500,805	999,595	-0,002	-1,885	31,1	DNALTLLIK		UN45A_HUMAN	Protein unc-45 homolog A
45,436	2	895,949	1789,884	-0,001	-0,339	122,91	SYELPDGQVITIGNER		ACTBM_HUMAN	Putative beta-actin-like protein 3
44,400	2	895,949	1789,884	-0,001	-0,313	28,06	SYELPDGQVITIGNER		ACTBM_HUMAN	Putative beta-actin-like protein 3
43,392	3	656,697	1967,070	-0,002	-1,235	25,89	VAPEEHPILLTEAPLNPK		ACTBM_HUMAN	Putative beta-actin-like protein 3
42,343	2	645,346	1288,677	-0,001	-0,658	40,73	SGPFAPVLSATSR		UCRIL_HUMAN	Putative cytochrome b-c1 complex subunit Rieske-like protein 1
55,923	2	731,381	1460,747	-0,015	-10,262	27,91	ISPEEVYNNLKR		NSUN7_HUMAN	Putative methyltransferase NSUN7
33,266	2	358,208	714,402	0,000	0,057	26,36	DIAAAVR		OR1F2_HUMAN	Putative olfactory receptor 1F2
30,670	2	420,737	839,460	-0,012	-14,823	26,42	RLNNAPR		SHSA8_HUMAN	Putative protein shisa-8
42,310	2	566,307	1130,599	-0,001	-1,083	55,59	MELQEIQLK		TPM3L_HUMAN	Putative tropomyosin alpha-3 chain-like protein

39,025	2	574,304	1146,594	-0,002	-1,399	32,25	MELQEIQLK	[1] (M) Oxidation	TPM3L_HUMAN	Putative tropomyosin alpha-3 chain-like protein
48,933	3	1006,535	3016,583	-0,004	-1,297	28,54	TATESFASDPILYRPVAVALDTKGPEIR		KPYM_HUMAN	Pyruvate kinase PKM
48,933	4	755,154	3016,586	-0,001	-0,411	35,81	TATESFASDPILYRPVAVALDTKGPEIR		KPYM_HUMAN	Pyruvate kinase PKM
40,782	2	581,312	1160,609	-0,013	-11,367	30,51	MGSPGASLGIKK	[1] (M) Oxidation	APRV1_HUMAN	Retroviral-like aspartic protease 1
32,835	2	476,717	951,420	-0,006	-6,383	31,68	SSEDSGSRK		RHG21_HUMAN	Rho GTPase-activating protein 21
46,782	3	558,657	1672,949	0,009	5,580	26,23	LEPEAPALALPVTPQK		RPAP1_HUMAN	RNA polymerase II-associated protein 1
40,618	2	661,376	1320,737	-0,003	-2,169	51,46	DEILLLHQAAAK		SLMAP_HUMAN	Sarcolemmal membrane-associated protein
35,916	2	696,362	1390,710	-0,002	-1,781	25,9	AEIGIAMGSGTAVAK	[7] (M) Oxidation	AT2A2_HUMAN	Sarcoplasmic/endoplasmic reticulum calcium ATPase 2
29,729	2	373,711	745,407	0,010	13,820	27,32	IEDGKGK		SEM3B_HUMAN	Semaphorin-3B
39,194	2	564,852	1127,690	-0,001	-0,925	30,84	KQTALVELVK		ALBU_HUMAN	Serum albumin
41,747	2	382,726	763,438	0,000	0,249	26,01	VFVFPR		SAMP_HUMAN	Serum amyloid P-component
41,211	2	578,803	1155,592	-0,001	-0,702	87,06	VGEYSLYIGR		SAMP_HUMAN	Serum amyloid P-

										component
36,247	2	583,795	1165,575	-0,002	-1,556	40,92	QGYFVEAQPK		SAMP_HUMAN	Serum amyloid P-component
39,248	2	889,439	1776,864	0,000	-0,113	33,2	SDGDPVQPAVL QVHQTS		SDPR_HUMAN	Serum deprivation-response protein
36,849	3	388,900	1163,678	-0,011	-9,209	27,79	SLNRVHLLGR		SSBP_HUMAN	Single-stranded DNA-binding protein, mitochondrial
37,063	2	537,282	1072,550	-0,002	-1,508	37,71	SLQQLAEER		SPTN1_HUMAN	Spectrin alpha chain, non-erythrocytic 1
41,180	2	554,797	1107,579	-0,002	-1,924	32,94	ITALDEFATK		SPTN1_HUMAN	Spectrin alpha chain, non-erythrocytic 1
46,103	2	602,842	1203,670	-0,002	-1,298	36,66	DLSSVQTLLTK		SPTN1_HUMAN	Spectrin alpha chain, non-erythrocytic 1
55,387	3	658,026	1971,056	-0,002	-1,111	48,63	IAALQAFADQLI AAGHYAK		SPTN1_HUMAN	Spectrin alpha chain, non-erythrocytic 1
35,312	2	485,295	968,574	-0,002	-2,325	35,54	VAVVNQIAR		SPTB2_HUMAN	Spectrin beta chain, non-erythrocytic 1
35,781	2	450,281	898,547	-0,013	-14,584	25,53	AAVVALSLR		DHSB_HUMAN	Succinate dehydrogenase [ubiquinone] iron-sulfur subunit, mitochondrial
57,912	2	1084,08 8	2166,162	0,000	-0,182	41,35	APPPIAYNPLLSP FFPQAAR		SYP2L_HUMAN	Synaptopodin 2-like protein
31,451	3	914,448	2740,323	0,014	5,103	27,53	MSQVMSSPLLAG	[1] Oxidation	TENS4_HUMAN	Tensin-4

							GHAVSLAPCDEP RR	(M) ǀ [5] Oxidation (M)		
41,992	2	448,302	894,589	-0,001	-0,924	27,47	VLGVPVIAK		TITIN_HUMAN	Titin
42,051	2	474,761	947,507	-0,001	-0,733	27,55	SVFPELTR		TITIN_HUMAN	Titin
40,726	2	476,800	951,585	-0,001	-1,489	25,75	QLGVPVIAR		TITIN_HUMAN	Titin
44,993	3	434,918	1301,733	-0,001	-1,121	29,55	SLRFPLALEEK		TITIN_HUMAN	Titin
47,852	2	658,366	1314,717	-0,001	-0,857	29,24	AGEDVQVLIPFK		TITIN_HUMAN	Titin
48,481	3	587,329	1758,965	-0,002	-1,085	26,1	LILTEGKNPPFFD IR		TITIN_HUMAN	Titin
42,468	3	694,699	2081,074	-0,001	-0,538	37,1	RSDTGLYTITAV NNLGTASK		TITIN_HUMAN	Titin
46,003	3	845,159	2532,455	-0,001	-0,557	28,88	TIKPPPVEPEPTPI AAPVTVPVVGK		TITIN_HUMAN	Titin
46,384	2	608,347	1214,680	-0,001	-0,790	34,59	TLMNLGGLAVA R		TAGL2_HUMAN	Transgelin-2
49,382	2	801,948	1601,881	-0,001	-0,381	31,2	VVLAYEPVWAIG TGK		TPIS_HUMAN	Triosephosphate isomerase
30,567	2	438,227	874,439	0,000	-0,438	30,27	SLEAQAEK		TPM1_HUMAN	Tropomyosin alpha-1 chain
42,310	2	566,307	1130,599	-0,001	-1,083	55,59	MEIQEIQLK		TPM1_HUMAN	Tropomyosin alpha-1 chain
39,025	2	574,304	1146,594	-0,002	-1,399	32,25	MEIQEIQLK	[1] Oxidation (M)	TPM1_HUMAN	Tropomyosin alpha-1 chain
40,842	2	657,884	1313,754	-0,001	-1,010	40,4	KLVIIESDLER		TPM1_HUMAN	Tropomyosin alpha-1 chain
40,842	3	438,925	1313,754	-0,001	-0,828	34,8	KLVIIESDLER		TPM1_HUMAN	Tropomyosin alpha-1

											chain
35,746	2	666,822	1331,630	-0,001	-1,122	60,41	ATDAEADVASLNR			TPM1_HUMAN	Tropomyosin alpha-1 chain
33,184	2	730,869	1459,724	-0,003	-1,913	39,4	KATDAEADVASLNR			TPM1_HUMAN	Tropomyosin alpha-1 chain
34,018	3	496,917	1487,729	-0,004	-2,443	39,43	ATDAEADVASLNRR			TPM1_HUMAN	Tropomyosin alpha-1 chain
33,413	3	496,917	1487,730	-0,003	-2,140	27,75	ATDAEADVASLNRR			TPM1_HUMAN	Tropomyosin alpha-1 chain
40,921	2	758,913	1515,812	-0,002	-1,407	27,34	SKQLEDELVSLQK			TPM1_HUMAN	Tropomyosin alpha-1 chain
45,315	2	887,911	1773,808	-0,001	-0,440	47,73	AISEELDHALNDMTSI	[13] (M)	Oxidation	TPM1_HUMAN	Tropomyosin alpha-1 chain
30,567	2	438,227	874,439	0,000	-0,438	30,27	SLEAQAEK			TPM3_HUMAN	Tropomyosin alpha-3 chain
42,310	2	566,307	1130,599	-0,001	-1,083	55,59	MELQEIQLK			TPM3_HUMAN	Tropomyosin alpha-3 chain
39,025	2	574,304	1146,594	-0,002	-1,399	32,25	MELQEIQLK	[1] (M)	Oxidation	TPM3_HUMAN	Tropomyosin alpha-3 chain
45,315	2	887,911	1773,808	-0,001	-0,440	47,73	AISEELDHALNDMTSI	[13] (M)	Oxidation	TPM3_HUMAN	Tropomyosin alpha-3 chain
35,746	2	666,822	1331,630	-0,001	-1,122	60,41	ATDAEADVASLNR			TPM2_HUMAN	Tropomyosin beta chain
33,184	2	730,869	1459,724	-0,003	-1,913	39,4	KATDAEADVASLNR			TPM2_HUMAN	Tropomyosin beta chain
34,018	3	496,917	1487,729	-0,004	-2,443	39,43	ATDAEADVASL			TPM2_HUMAN	Tropomyosin beta

							NRR			chain
33,413	3	496,917	1487,730	-0,003	-2,140	27,75	ATDAEADVASL NRR		TPM2_HUMAN	Tropomyosin beta chain
38,823	2	581,779	1161,544	-0,001	-0,624	90,23	NIDALSGMEGR		TNNI3_HUMAN	Troponin I, cardiac muscle
33,111	2	589,778	1177,542	0,003	2,263	61,87	NIDALSGMEGR	[8] Oxidation (M)	TNNI3_HUMAN	Troponin I, cardiac muscle
31,314	2	653,823	1305,631	-0,003	-2,476	44,38	KNIDALSGMEGR	[9] Oxidation (M)	TNNI3_HUMAN	Troponin I, cardiac muscle
54,238	2	724,373	1446,732	-0,001	-0,363	86,95	ISADAMMQALL GAR		TNNI3_HUMAN	Troponin I, cardiac muscle
45,625	2	732,370	1462,726	-0,001	-0,893	65,86	ISADAMMQALL GAR	[6] Oxidation (M)	TNNI3_HUMAN	Troponin I, cardiac muscle
45,625	2	732,370	1462,726	-0,001	-0,893	86,92	ISADAMMQALL GAR	[7] Oxidation (M)	TNNI3_HUMAN	Troponin I, cardiac muscle
38,339	2	740,367	1478,720	-0,002	-1,159	42,27	ISADAMMQALL GAR	[6] Oxidation (M)|[7] Oxidation (M)	TNNI3_HUMAN	Troponin I, cardiac muscle
59,363	3	630,682	1889,024	-0,002	-0,995	44,46	NITEIADLTQKIF DLR		TNNI3_HUMAN	Troponin I, cardiac muscle
30,670	2	379,237	756,460	-0,001	-0,764	35,39	ILAERR		TNNT2_HUMAN	Troponin T, cardiac muscle
40,301	2	453,755	905,495	-0,002	-2,425	26,65	YEINVLR		TNNT2_HUMAN	Troponin T, cardiac muscle
35,810	2	508,742	1015,470	-0,002	-2,422	32,31	VDFDDIHR		TNNT2_HUMAN	Troponin T, cardiac muscle

35,810	3	339,498	1015,473	0,000	0,289	25,44	VDFDDIHR			TNNT2_HUMAN	Troponin T, cardiac muscle
39,499	2	806,884	1611,753	-0,001	-0,572	36,11	ALSNMMHFGGYIQK	[5] (M)	Oxidation	TNNT2_HUMAN	Troponin T, cardiac muscle
46,168	3	599,650	1795,928	-0,001	-0,796	29,11	SFMPNLVPPKIPDGER			TNNT2_HUMAN	Troponin T, cardiac muscle
42,726	3	604,981	1811,922	-0,002	-1,330	35,6	SFMPNLVPPKIPDGER	[3] (M)	Oxidation	TNNT2_HUMAN	Troponin T, cardiac muscle
54,459	3	637,990	1910,947	-0,002	-0,859	60,09	DLNELQALIEAHFENR			TNNT2_HUMAN	Troponin T, cardiac muscle
54,459	2	956,481	1910,948	-0,001	-0,567	40,28	DLNELQALIEAHFENR			TNNT2_HUMAN	Troponin T, cardiac muscle
50,982	4	575,788	2299,125	-0,002	-0,822	37,69	MEKDLNELQALIEAHFENR			TNNT2_HUMAN	Troponin T, cardiac muscle
50,029	4	579,787	2315,119	-0,003	-1,222	38,14	MEKDLNELQALIEAHFENR	[1] (M)	Oxidation	TNNT2_HUMAN	Troponin T, cardiac muscle
50,029	3	772,714	2315,120	-0,001	-0,599	28,08	MEKDLNELQALIEAHFENR	[1] (M)	Oxidation	TNNT2_HUMAN	Troponin T, cardiac muscle
35,810	2	508,742	1015,470	-0,002	-2,422	32,31	VDFDDIHR			TNNT1_HUMAN	Troponin T, slow skeletal muscle
35,810	3	339,498	1015,473	0,000	0,289	25,44	VDFDDIHR			TNNT1_HUMAN	Troponin T, slow skeletal muscle
50,313	2	851,456	1700,897	-0,001	-0,657	64,18	AVFVDLEPTVIDEVR			TBA1A_HUMAN	Tubulin alpha-1A chain
49,552	3	760,734	2279,180	0,001	0,251	27,69	AVFVDLEPTVIDEVRTGTYR			TBA1A_HUMAN	Tubulin alpha-1A chain

52,349	2	858,464	1714,914	0,000	-0,166	40,92	AVFVDLEPTVIDEIR			TBA4A_HUMAN	Tubulin alpha-4A chain
46,003	2	572,320	1142,625	-0,002	-1,335	28,94	LAVNMVPFPR			TBB5_HUMAN	Tubulin beta chain
42,114	2	580,317	1158,620	-0,002	-1,633	30,95	LAVNMVPFPR	[5] (M)	Oxidation	TBB5_HUMAN	Tubulin beta chain
42,726	2	660,354	1318,694	-0,002	-1,315	49,82	IMNTFSVVPSPK			TBB5_HUMAN	Tubulin beta chain
50,866	3	540,949	1619,826	-0,002	-1,416	34,82	LHFFMPGFAPLTSR			TBB5_HUMAN	Tubulin beta chain
44,499	2	816,419	1630,823	-0,001	-0,551	39,61	AILVDLEPGTMDSVR	[11] (M)	Oxidation	TBB5_HUMAN	Tubulin beta chain
49,998	2	830,450	1658,886	-0,002	-1,119	41,96	ALTVPELTQQVFDAK			TBB5_HUMAN	Tubulin beta chain
50,639	3	696,363	2086,068	-0,002	-0,897	54,68	GHYTEGAELVDSVLDVVRK			TBB5_HUMAN	Tubulin beta chain
51,567	3	933,453	2797,336	0,000	-0,069	75,79	SGPFGQIFRPDNFVFGQSGAGNNWAK			TBB5_HUMAN	Tubulin beta chain
46,003	2	572,320	1142,625	-0,002	-1,335	28,94	LAVNMVPFPR			TBB1_HUMAN	Tubulin beta-1 chain
42,114	2	580,317	1158,620	-0,002	-1,633	30,95	LAVNMVPFPR	[5] (M)	Oxidation	TBB1_HUMAN	Tubulin beta-1 chain
47,387	2	808,421	1614,828	-0,001	-0,575	35,86	AVLVDLEPGTMDSIR			TBB1_HUMAN	Tubulin beta-1 chain
46,003	2	572,320	1142,625	-0,002	-1,335	28,94	LAVNMVPFPR			TBB3_HUMAN	Tubulin beta-3 chain
42,114	2	580,317	1158,620	-0,002	-1,633	30,95	LAVNMVPFPR	[5] (M)	Oxidation	TBB3_HUMAN	Tubulin beta-3 chain
42,726	2	660,354	1318,694	-0,002	-1,315	49,82	IMNTFSVVPSPK			TBB3_HUMAN	Tubulin beta-3 chain

44,499	2	816,419	1630,823	-0,001	-0,551	39,61	AILVDLEPGTMD SVR	[11] (M)	Oxidation	TBB3_HUMAN	Tubulin beta-3 chain
50,639	3	696,363	2086,068	-0,002	-0,897	54,68	GHYTEGAELVDS VLDVVRK			TBB3_HUMAN	Tubulin beta-3 chain
46,003	2	572,320	1142,625	-0,002	-1,335	28,94	LAVNMVPFPR			TBB4B_HUMAN	Tubulin beta-4B chain
42,114	2	580,317	1158,620	-0,002	-1,633	30,95	LAVNMVPFPR	[5] (M)	Oxidation	TBB4B_HUMAN	Tubulin beta-4B chain
42,726	2	660,354	1318,694	-0,002	-1,315	49,82	IMNTFSVVPSPK			TBB4B_HUMAN	Tubulin beta-4B chain
45,668	2	801,413	1600,812	-0,001	-0,724	30,14	AVLVDLEPGTM DSVR			TBB4B_HUMAN	Tubulin beta-4B chain
42,836	2	809,411	1616,807	-0,001	-0,833	31,95	AVLVDLEPGTM DSVR	[11] (M)	Oxidation	TBB4B_HUMAN	Tubulin beta-4B chain
50,866	3	540,949	1619,826	-0,002	-1,416	34,82	LHFFMPGFAPLT SR			TBB4B_HUMAN	Tubulin beta-4B chain
50,639	3	696,363	2086,068	-0,002	-0,897	54,68	GHYTEGAELVDS VLDVVRK			TBB4B_HUMAN	Tubulin beta-4B chain
51,567	3	933,453	2797,336	0,000	-0,069	75,79	SGPFGQIFRPDNF VFGQSGAGNNW AK			TBB4B_HUMAN	Tubulin beta-4B chain
46,003	2	572,320	1142,625	-0,002	-1,335	28,94	LAVNMVPFPR			TBB6_HUMAN	Tubulin beta-6 chain
42,114	2	580,317	1158,620	-0,002	-1,633	30,95	LAVNMVPFPR	[5] (M)	Oxidation	TBB6_HUMAN	Tubulin beta-6 chain
50,866	3	540,949	1619,826	-0,002	-1,416	34,82	LHFFMPGFAPLT SR			TBB6_HUMAN	Tubulin beta-6 chain
46,003	2	572,320	1142,625	-0,002	-1,335	28,94	LAVNMVPFPR			TBB8_HUMAN	Tubulin beta-8 chain
42,114	2	580,317	1158,620	-0,002	-1,633	30,95	LAVNMVPFPR	[5]	Oxidation	TBB8_HUMAN	Tubulin beta-8 chain

								(M)		
45,668	2	801,413	1600,812	-0,001	-0,724	30,14	AVLVDLEPGTM DSVR		TBB8_HUMAN	Tubulin beta-8 chain
42,836	2	809,411	1616,807	-0,001	-0,833	31,95	AVLVDLEPGTM DSVR	[11] Oxidation (M)	TBB8_HUMAN	Tubulin beta-8 chain
50,866	3	540,949	1619,826	-0,002	-1,416	34,82	LHFFMPGFAPLT SR		TBB8_HUMAN	Tubulin beta-8 chain
39,218	2	660,858	1319,702	-0,002	-1,688	38,4	TSAALSTVGSAIS R		TPD54_HUMAN	Tumor protein D54
42,996	2	573,314	1144,614	0,017	14,789	26,11	EEEKLELQK		U520_HUMAN	U5 small nuclear ribonucleoprotein 200 kDa helicase
41,992	2	448,302	894,589	-0,001	-0,924	34,35	VLGVLPGIK		ADCK2_HUMAN	Uncharacterized aarF domain-containing protein kinase 2
44,537	4	543,014	2168,028	0,012	5,425	25,4	DGVADSTVISSM PCLLMELR	[12] Oxidation (M) | [17] Oxidation (M)	CR025_HUMAN	Uncharacterized protein C18orf25
29,729	2	373,711	745,407	-0,001	-1,206	28,11	QGTQGKK		YA044_HUMAN	Uncharacterized protein ENSP00000471857
42,836	2	538,755	1075,495	-0,002	-1,911	27,12	DNLAEDIMR		VIME_HUMAN	Vimentin
35,359	2	547,266	1092,518	-0,002	-2,020	72,12	FADLSEAANR		VIME_HUMAN	Vimentin
36,120	2	635,783	1269,552	-0,003	-2,049	37,39	LGDLYEEEMR	[9] Oxidation (M)	VIME_HUMAN	Vimentin
39,888	2	655,306	1308,597	-0,001	-1,082	49,06	NLQEAEEWYK		VIME_HUMAN	Vimentin

37,177	2	714,859	1427,704	-0,001	-0,664	40,41	SLYASSPGGVYA TR	VIME_HUMAN	Vimentin
55,504	2	785,951	1569,887	-0,001	-0,721	59,68	ISLPLPNFSSLNL R	VIME_HUMAN	Vimentin
54,933	2	1063,53 5	2125,056	-0,002	-0,891	46,56	LLQDSVDFSLAD AINTEFK	VIME_HUMAN	Vimentin
52,966	3	833,090	2496,249	0,000	-0,171	42,86	LLQDSVDFSLAD AINTEFKNTR	VIME_HUMAN	Vimentin
34,575	2	406,789	811,564	-0,001	-0,766	32,64	ILLVAKR	VINC_HUMAN	Vinculin
70,071	3	692,731	2075,172	-0,001	-0,685	27,07	AIPDLTAPVAAV QAAVSNLVR	VINC_HUMAN	Vinculin
39,591	3	501,949	1502,825	-0,003	-1,686	25,29	IYISGMAPRPSLA K	VTNC_HUMAN	Vitronectin
35,174	2	470,735	939,455	-0,002	-1,649	32,32	NNFAVGYR	VDAC2_HUMAN	Voltage-dependent anion-selective channel protein 2

Supplementary Table S3. List of peptides and their corresponding proteins in all tissue sections identified by MALDI IMS assigned to nano LC-MS (LCMS-MALDI-MSI-match)

MALDI-MSI m/z value [Da]	ROC [AUC] Adventitia AAA vs. EVAR	ROC [AUC] Adventitia AAA vs. TAA	ROC [AUC] Adventitia EVAR vs. TAA	ROC [AUC] Media AAA vs. EVAR	ROC [AUC] Media AAA vs. TAA	ROC [AUC] Media EVAR vs. TAA	LC-MS [MH+Calc.]	Scores	Sequence	Gene Symbol	Protein
644,40	0,49	0,41	0,41	0,58	0,42	0,33	643,41	33,20	AQIALK	MDHC_HUMAN	Malate dehydrogenase, cytoplasmic
645,40	0,49	0,42	0,41	0,57	0,42	0,34					
678,40	0,44	0,37	0,41	0,57	0,43	0,34					
679,40	0,46	0,40	0,41	0,55	0,46	0,38					
688,40	0,51	0,51	0,48	0,61	0,44	0,33	688,43	27,64	KQLATK	H31T_HUMAN	Histone H3.1t
700,40	0,51	0,47	0,46	0,55	0,44	0,39					
701,41	0,49	0,47	0,48	0,55	0,38	0,33					
730,41	0,50	0,43	0,42	0,58	0,40	0,32					
758,41	0,50	0,45	0,46	0,55	0,45	0,40	757,47	35,39	ILAERR	TNNT2_HUMAN	Troponin T, cardiac muscle
771,41	0,52	0,44	0,41	0,57	0,45	0,36					
784,41	0,51	0,46	0,45	0,58	0,45	0,37	785,39	48,12	GDQGPVGR	CO1A2_HUMAN	Collagen alpha-2(I) chain
785,41	0,50	0,44	0,42	0,57	0,43	0,35	785,39	48,12	GDQGPVGR	CO1A2_HUMAN	Collagen alpha-2(I)

											chain
786,41	0,50	0,45	0,44	0,55	0,44	0,38					
795,41	0,49	0,43	0,42	0,63	0,35	0,21					
796,41	0,48	0,43	0,44	0,62	0,39	0,27					
833,42	0,48	0,49	0,51	0,59	0,40	0,31					
836,42	0,52	0,43	0,41	0,59	0,42	0,31	836,44	49,08	GPAGPQGPR	CO1A1_HUMAN	Collagen alpha-1(I) chain
837,42	0,56	0,45	0,41	0,60	0,43	0,31	836,44	49,08	GPAGPQGPR	CO1A1_HUMAN	Collagen alpha-1(I) chain
838,42	0,50	0,44	0,44	0,57	0,45	0,38					
840,42	0,47	0,45	0,48	0,59	0,48	0,38	840,47	26,42	RLNNAPR	SHSA8_HUMAN	Putative protein shisa-8
841,42	0,47	0,42	0,45	0,59	0,46	0,37	840,47	26,42	RLNNAPR	SHSA8_HUMAN	Putative protein shisa-8
842,42	0,52	0,45	0,46	0,55	0,42	0,39					
852,42	0,54	0,45	0,42	0,61	0,40	0,28	851,42	43,88	GFSGLDGAK	CO1A1_HUMAN	Collagen alpha-1(I) chain
853,42	0,52	0,43	0,42	0,61	0,40	0,27	853,52	55,31	LGPLQVAR	ETFB_HUMAN	Electron transfer flavoprotein subunit beta
854,42	0,51	0,43	0,41	0,60	0,42	0,32	853,52	55,31	LGPLQVAR	ETFB_HUMAN	Electron transfer flavoprotein subunit beta
868,42	0,50	0,42	0,43	0,59	0,42	0,31	868,43	34,63	GEAGPQGPR	CO1A1_HUMAN	Collagen alpha-1(I) chain
869,42	0,50	0,43	0,44	0,57	0,43	0,36	868,43	34,63	GEAGPQGPR	CO1A1_HUMAN	Collagen alpha-1(I)

										chain
870,42	0,51	0,44	0,42	0,56	0,46	0,40				
872,42	0,52	0,45	0,43	0,55	0,37	0,33				
874,42	0,48	0,43	0,45	0,59	0,42	0,32				
875,42	0,51	0,48	0,46	0,57	0,44	0,37	875,45	30,27	SLEAQAEK	TPM1_HUMAN	Tropomyosin alpha-1 chain
886,42	0,49	0,40	0,41	0,56	0,43	0,36	886,44	47,39	GSEGPQGVR	CO1A1_HUMAN	Collagen alpha-1(I) chain
887,42	0,49	0,40	0,41	0,55	0,45	0,38	886,44	47,39	GSEGPQGVR	CO1A1_HUMAN	Collagen alpha-1(I) chain
890,42	0,54	0,49	0,45	0,57	0,39	0,30					
891,42	0,54	0,51	0,48	0,56	0,42	0,35					
899,42	0,54	0,47	0,42	0,56	0,46	0,39	899,55	25,53	AAVVALSLR	DHSB_HUMAN	Succinate dehydrogenase [ubiquinone] iron-sulfur subunit, mitochondrial
906,42	0,51	0,50	0,48	0,55	0,43	0,37	905,52	28,35	LVPQQLAH	COX5B_HUMAN	Cytochrome c oxidase subunit 5B, mitochondrial
944,43	0,53	0,51	0,48	0,57	0,45	0,37	943,57	36,36	VPPPPPIAR	HNRPC_HUMAN	Heterogeneous nuclear ribonucleoproteins C1/C2
945,43	0,51	0,46	0,46	0,56	0,45	0,38	945,50	30,15	LSENVIDR	CHCH3_HUMAN	Coiled-coil-helix-coiled-coil-helix

											domain-containing protein 3, mitochondrial
974,43	0,49	0,46	0,44	0,56	0,43	0,38	973,60	45,36	SGVSLAALKK	H13_HUMAN	Histone H1.3
976,43	0,52	0,43	0,40	0,62	0,35	0,22	976,45	88,56	AGFAGDDAPR	ACTA_HUMAN	Actin, aortic smooth muscle
1094,64	0,52	0,40	0,37	0,63	0,37	0,23	1095,56	25,48	KITIADCGQF	PAL4A_HUMAN	Peptidyl-prolyl cis-trans isomerase A-like 4A/B/C
1095,64	0,45	0,38	0,42	0,58	0,39	0,31	1095,56	25,48	KITIADCGQF	PAL4A_HUMAN	Peptidyl-prolyl cis-trans isomerase A-like 4A/B/C
1096,64	0,51	0,43	0,40	0,59	0,42	0,32	1097,62	51,55	VLPGVDALSNI	PGK1_HUMAN	Phosphoglycerate kinase 1
1097,64	0,50	0,44	0,43	0,55	0,45	0,40	1097,62	51,55	VLPGVDALSNI	PGK1_HUMAN	Phosphoglycerate kinase 1
1105,64	0,47	0,40	0,41	0,60	0,42	0,31					
1106,64	0,41	0,36	0,42	0,58	0,42	0,32	1106,68	27,56	VLTLELYKK	KCRM_HUMAN	Creatine kinase M-type
1107,64	0,50	0,40	0,40	0,61	0,44	0,31	1106,68	27,56	VLTLELYKK	KCRM_HUMAN	Creatine kinase M-type
1108,64	0,51	0,44	0,43	0,56	0,45	0,38	1108,59	32,94	ITALDEFATK	SPTN1_HUMAN	Spectrin alpha chain, non-erythrocytic 1
1111,64	0,48	0,42	0,43	0,61	0,36	0,26					
1112,64	0,50	0,43	0,42	0,62	0,39	0,26					
1113,64	0,44	0,43	0,50	0,55	0,42	0,37					

1133,44	0,51	0,50	0,47	0,59	0,43	0,34					
1134,44	0,53	0,52	0,48	0,56	0,43	0,38	1134,65	42,48	SLNILTAFQK	ERP29_HUMAN	Endoplasmic reticulum resident protein 29
1138,64	0,43	0,38	0,43	0,59	0,35	0,25					
1139,64	0,42	0,37	0,45	0,60	0,38	0,27					
1140,64	0,47	0,42	0,45	0,60	0,41	0,31					
1143,45	0,52	0,51	0,49	0,60	0,42	0,31	1143,63	28,94	LAVNMVPFPR	TBB5_HUMAN	Tubulin beta chain
1144,45	0,47	0,47	0,49	0,58	0,45	0,36	1143,63	28,94	LAVNMVPFPR	TBB5_HUMAN	Tubulin beta chain
1149,65	0,47	0,46	0,47	0,58	0,43	0,35					
1150,65	0,49	0,49	0,50	0,56	0,43	0,38	1150,67	45,55	VLIAAHGNSLR	PGAM2_HUMAN	Phosphoglycerate mutase 2
1154,65	0,44	0,37	0,44	0,63	0,40	0,28					
1155,45	0,51	0,48	0,48	0,57	0,45	0,38					
1176,45	0,53	0,55	0,50	0,58	0,39	0,30					
1198,65	0,49	0,38	0,39	0,66	0,29	0,10	1198,52	57,19	DSYVGDEAQSK	ACTA_HUMAN	Actin, aortic smooth muscle
1199,65	0,51	0,42	0,41	0,65	0,30	0,12	1198,71	33,51	AVFPSIVGRPR	ACTA_HUMAN	Actin, aortic smooth muscle
1200,65	0,45	0,42	0,46	0,61	0,35	0,22					
1231,65	0,52	0,50	0,47	0,58	0,44	0,35	1232,64	31,53	AMTKDNNLLGR	HSP71_HUMAN	Heat shock 70 kDa protein 1A/1B
1235,65	0,46	0,38	0,41	0,57	0,44	0,36					
1236,65	0,49	0,40	0,40	0,57	0,42	0,34					
1237,65	0,52	0,45	0,41	0,57	0,43	0,37	1238,65	47,18	VAVFFSNTPTR	CO6A3_HUMAN	Collagen alpha-3(VI) chain

1267,66	0,47	0,42	0,45	0,57	0,40	0,34					
1268,66	0,54	0,47	0,43	0,59	0,43	0,34					
1269,66	0,52	0,45	0,42	0,56	0,43	0,37	1270,56	37,39	LGDLYEEEMR	VIME_HUMAN	Vimentin
1303,66	0,48	0,39	0,40	0,62	0,36	0,24	1303,60	55,78	NALESYAFNMK	HSP71_HUMAN	Heat shock 70 kDa protein 1A/1B
1304,66	0,50	0,42	0,42	0,62	0,39	0,27	1303,68	41,21	PMFIVNTNVPR	MIF_HUMAN	Macrophage migration inhibitory factor
1305,66	0,47	0,44	0,45	0,58	0,41	0,34	1306,64	44,38	KNIDALSGMEGR	TNNI3_HUMAN	Troponin I, cardiac muscle
1320,66	0,57	0,48	0,41	0,64	0,38	0,24	1319,70	49,82	IMNTFSVVPSPK	TBB5_HUMAN	Tubulin beta chain
1321,66	0,55	0,47	0,42	0,63	0,37	0,24	1321,74	51,46	DEILLLHQAAAK	SLMAP_HUMAN	Sarcolemmal membrane-associated protein
1322,66	0,53	0,46	0,45	0,60	0,38	0,26	1321,74	51,46	DEILLLHQAAAK	SLMAP_HUMAN	Sarcolemmal membrane-associated protein
1324,66	0,54	0,48	0,43	0,59	0,43	0,34	1324,71	33,76	SASYNLSLTLQK	LDB3_HUMAN	LIM domain-binding protein 3
1325,66	0,45	0,45	0,48	0,59	0,44	0,36	1325,75	45,61	DNIQGITKPAIR	H4_HUMAN	Histone H4
1337,66	0,48	0,40	0,45	0,57	0,39	0,33					
1340,66	0,50	0,46	0,46	0,60	0,44	0,34	1339,72	51,07	LSVISVEDPPQR	ETFB_HUMAN	Electron transfer flavoprotein subunit beta
1341,66	0,46	0,44	0,46	0,58	0,42	0,34	1341,77	27,41	ALVQNDTLLQVK	H1X_HUMAN	Histone H1x
1342,66	0,48	0,45	0,47	0,57	0,43	0,35	1342,78	32,68	VIQVAAGSSNLKR	ALDH2_HUMAN	Aldehyde

											dehydrogenase, mitochondrial
1358,66	0,49	0,46	0,48	0,60	0,41	0,30	1358,74	34,89	ISVREPMQTGIK	ATPA_HUMAN	ATP synthase subunit alpha, mitochondrial
1359,66	0,52	0,47	0,47	0,63	0,40	0,26	1358,74	34,89	ISVREPMQTGIK	ATPA_HUMAN	ATP synthase subunit alpha, mitochondrial
1366,67	0,49	0,41	0,41	0,59	0,37	0,29					
1401,67	0,48	0,43	0,44	0,55	0,44	0,39					
1408,67	0,55	0,46	0,42	0,60	0,31	0,20					
1409,67	0,49	0,44	0,46	0,56	0,34	0,25					
1428,67	0,50	0,44	0,44	0,55	0,33	0,28	1428,71	40,41	SLYASSPGGVYATR	VIME_HUMAN	Vimentin
1459,67	0,54	0,46	0,41	0,61	0,43	0,32	1459,86	25,39	IGDLHPQIVNLLK	CO6A3_HUMAN	Collagen alpha-3(VI) chain
1460,67	0,56	0,47	0,40	0,62	0,43	0,31	1459,86	25,39	IGDLHPQIVNLLK	CO6A3_HUMAN	Collagen alpha-3(VI) chain
1461,67	0,50	0,42	0,41	0,58	0,42	0,33	1461,75	27,91	ISPEEVYNNLKR	NSUN7_HUMAN	Putative methyltransferase NSUN7
1462,67	0,48	0,44	0,45	0,55	0,44	0,39	1462,76	27,26	QINVGNALEYVSR	CO6A3_HUMAN	Collagen alpha-3(VI) chain
1465,67	0,47	0,37	0,39	0,56	0,39	0,31					
1466,67	0,55	0,43	0,38	0,60	0,42	0,32	1466,80	61,03	TVTAMDVVYALKR	H4_HUMAN	Histone H4
1467,67	0,52	0,41	0,39	0,57	0,42	0,33	1466,80	61,03	TVTAMDVVYALKR	H4_HUMAN	Histone H4
1477,68	0,54	0,46	0,43	0,60	0,43	0,33					

1478,68	0,55	0,47	0,43	0,59	0,45	0,35	1478,90	70,53	HGATVLTALGGILKK	MYG_HUMAN	Myoglobin
1479,68	0,48	0,44	0,47	0,54	0,43	0,40	1478,90	70,53	HGATVLTALGGILKK	MYG_HUMAN	Myoglobin
1493,68	0,49	0,35	0,37	0,59	0,45	0,34					
1494,68	0,47	0,33	0,37	0,58	0,36	0,28					
1495,68	0,52	0,37	0,37	0,59	0,34	0,26					
1502,68	0,51	0,52	0,52	0,54	0,43	0,40					
1503,68	0,50	0,49	0,48	0,56	0,46	0,40	1503,83	25,29	IYISGMAPRPSLAK	VTNC_HUMAN	Vitronectin
1508,68	0,49	0,41	0,42	0,60	0,40	0,32	1507,80	83,26	LSVEALNSLTGEFK	KCRM_HUMAN	Creatine kinase M-type
1509,68	0,48	0,42	0,43	0,57	0,40	0,33					
1510,68	0,50	0,41	0,40	0,57	0,42	0,36					
1515,68	0,52	0,43	0,41	0,61	0,41	0,28					
1516,68	0,52	0,45	0,42	0,61	0,43	0,32	1516,82	27,34	SKQLEDELVSLQK	TPM1_HUMAN	Tropomyosin alpha-1 chain
1530,68	0,46	0,42	0,45	0,57	0,43	0,36	1529,73	45,35	VGAHAGEYGAEALER	HBA_HUMAN	Hemoglobin subunit alpha
1531,68	0,50	0,42	0,41	0,59	0,41	0,31					
1532,68	0,45	0,39	0,44	0,55	0,42	0,36					
1533,68	0,55	0,47	0,42	0,58	0,44	0,34	1533,78	82,04	VVDSLQTSLDAETR	MYH6_HUMAN	Myosin-6
1534,68	0,51	0,44	0,43	0,57	0,47	0,40	1533,78	82,04	VVDSLQTSLDAETR	MYH6_HUMAN	Myosin-6
1546,88	0,51	0,37	0,36	0,61	0,44	0,31					
1547,88	0,45	0,33	0,37	0,61	0,43	0,33					
1548,88	0,48	0,37	0,39	0,58	0,45	0,37					
1549,68	0,54	0,44	0,43	0,56	0,43	0,37					
1561,88	0,48	0,38	0,39	0,60	0,48	0,37	1562,79	87,57	GETGPSGPVGPAGAVGPR	CO1A2_HUMAN	Collagen alpha-2(I) chain

1562,88	0,46	0,33	0,36	0,62	0,40	0,28	1562,79	87,57	GETGPSGPVGPAGAVGPR	CO1A2_HUMAN	Collagen alpha-2(I) chain
1563,88	0,47	0,35	0,36	0,62	0,41	0,28					
1564,88	0,49	0,37	0,38	0,59	0,42	0,33	1564,91	31,49	SIVVSPILIPENQR	CAD13_HUMAN	Cadherin-13
1565,88	0,45	0,38	0,42	0,54	0,44	0,40	1564,91	31,49	SIVVSPILIPENQR	CAD13_HUMAN	Cadherin-13
1580,68	0,54	0,45	0,41	0,56	0,45	0,39	1580,80	43,04	MQKEITALAPSTMK	ACTA_HUMAN	Actin, aortic smooth muscle
1581,69	0,56	0,47	0,40	0,59	0,47	0,39	1580,80	43,04	MQKEITALAPSTMK	ACTA_HUMAN	Actin, aortic smooth muscle
1585,69	0,49	0,42	0,43	0,57	0,42	0,35					
1586,69	0,57	0,47	0,42	0,61	0,44	0,32					
1587,69	0,50	0,43	0,42	0,57	0,43	0,35					
1588,69	0,52	0,46	0,44	0,55	0,43	0,39					
1602,69	0,52	0,47	0,44	0,56	0,44	0,37	1602,89	31,20	VVLAYEPVWAIGTGK	TPIS_HUMAN	Triosephosphate isomerase
1623,69	0,52	0,48	0,47	0,58	0,44	0,35					
1624,69	0,50	0,46	0,47	0,57	0,43	0,35					
1637,89	0,51	0,46	0,45	0,57	0,44	0,38					
1655,89	0,45	0,38	0,40	0,58	0,43	0,35					
1656,89	0,47	0,39	0,40	0,58	0,44	0,35					
1669,89	0,47	0,38	0,39	0,56	0,41	0,34					
1670,89	0,48	0,37	0,37	0,59	0,38	0,29					
1671,89	0,48	0,39	0,39	0,57	0,41	0,34					
1672,89	0,48	0,41	0,43	0,54	0,44	0,39					
1685,69	0,48	0,45	0,44	0,56	0,42	0,36					
1686,69	0,47	0,44	0,46	0,55	0,41	0,36					

1687,69	0,49	0,45	0,46	0,56	0,44	0,38	1687,97	47,44	VGGHLRPGIVQSGGVVR	PGBM_HUMAN	Basement membrane-specific heparan sulfate proteoglycan core protein
1691,90	0,46	0,39	0,41	0,56	0,39	0,33					
1692,90	0,45	0,40	0,41	0,58	0,44	0,36					
1694,70	0,54	0,54	0,47	0,57	0,45	0,39					
1706,70	0,50	0,37	0,37	0,57	0,44	0,37					
1707,70	0,53	0,41	0,38	0,58	0,45	0,36					
1708,70	0,51	0,41	0,39	0,57	0,44	0,35					
1709,70	0,48	0,41	0,43	0,55	0,43	0,39					
1710,90	0,46	0,41	0,42	0,60	0,36	0,26	1710,81	25,14	FEAAETLEEAAMRSR	KLC1_HUMAN	Kinesin light chain 1
1711,90	0,52	0,46	0,43	0,63	0,39	0,25					
1712,90	0,49	0,44	0,45	0,60	0,39	0,30					
1723,70	0,51	0,48	0,46	0,57	0,42	0,35	1723,86	47,13	SPPNPDNIAPGYSGPLK	MYOZ2_HUMAN	Myozenin-2
1724,70	0,54	0,52	0,46	0,58	0,43	0,35	1723,86	47,13	SPPNPDNIAPGYSGPLK	MYOZ2_HUMAN	Myozenin-2
1728,70	0,52	0,48	0,44	0,56	0,45	0,40					
1729,70	0,52	0,48	0,47	0,55	0,45	0,40	1729,88	31,28	VSDLTQAANKNNDALR	DESM_HUMAN	Desmin
1742,70	0,53	0,44	0,39	0,57	0,46	0,38					
1743,70	0,53	0,43	0,42	0,59	0,47	0,38	1743,82	88,66	AMGIMNSFVNDIFER	H2B1B_HUMAN	Histone H2B type 1-B
1744,70	0,51	0,42	0,41	0,58	0,44	0,35	1743,82	88,66	AMGIMNSFVNDIFER	H2B1B_HUMAN	Histone H2B type 1-B
1745,70	0,53	0,46	0,43	0,59	0,44	0,34					
1746,70	0,53	0,48	0,44	0,57	0,43	0,36					

1751,90	0,46	0,38	0,41	0,56	0,42	0,35					
1752,90	0,49	0,39	0,39	0,59	0,44	0,34					
1753,90	0,46	0,39	0,42	0,56	0,46	0,39					
1766,90	0,47	0,38	0,40	0,57	0,46	0,37					
1767,90	0,49	0,39	0,38	0,57	0,47	0,39	1768,83	32,75	DGEVVSEATQQQHEVL	DESM_HUMAN	Desmin
1775,90	0,50	0,41	0,40	0,59	0,46	0,35	1775,81	48,88	AMGIMNSFVNDIFER	H2B1B_HUMAN	Histone H2B type 1-B
1776,90	0,49	0,40	0,42	0,59	0,46	0,35	1777,87	33,20	SDGDPVQPAVLQVHQTS	SDPR_HUMAN	Serum deprivation-response protein
1777,90	0,47	0,38	0,41	0,56	0,44	0,37	1777,87	33,20	SDGDPVQPAVLQVHQTS	SDPR_HUMAN	Serum deprivation-response protein
1790,90	0,47	0,39	0,42	0,57	0,36	0,27	1790,89	28,06	SYELPDGQVITIGNER	ACTA_HUMAN	Actin, aortic smooth muscle
1791,90	0,51	0,42	0,41	0,59	0,36	0,26					
1797,90	0,49	0,46	0,44	0,57	0,46	0,40	1796,93	29,11	SFMPNLVPPKIPDGER	TNNT2_HUMAN	Troponin T, cardiac muscle
1798,90	0,50	0,44	0,44	0,57	0,45	0,38					
1816,91	0,45	0,33	0,36	0,59	0,44	0,32					
1817,91	0,49	0,36	0,36	0,60	0,45	0,31					
1818,91	0,49	0,37	0,38	0,60	0,46	0,34					
1819,91	0,49	0,42	0,42	0,59	0,47	0,38					
1820,91	0,52	0,48	0,46	0,57	0,47	0,39					
1821,91	0,52	0,50	0,47	0,56	0,46	0,40					
1832,91	0,50	0,37	0,36	0,60	0,42	0,30					
1833,91	0,49	0,35	0,35	0,60	0,39	0,28					
1834,91	0,46	0,35	0,38	0,59	0,42	0,32					

1835,91	0,46	0,37	0,40	0,58	0,44	0,34	1836,84	32,84	MTAFDADDPATDNALLR	CAD13_HUMAN	Cadherin-13
1836,91	0,47	0,40	0,42	0,57	0,46	0,37	1836,84	32,84	MTAFDADDPATDNALLR	CAD13_HUMAN	Cadherin-13
1850,91	0,52	0,46	0,44	0,59	0,44	0,34	1851,04	48,84	VQLLHSQNTSLINQKK	MYH6_HUMAN	Myosin-6
1851,91	0,48	0,43	0,45	0,58	0,43	0,34	1851,04	48,84	VQLLHSQNTSLINQKK	MYH6_HUMAN	Myosin-6
1852,91	0,45	0,41	0,45	0,56	0,45	0,38					
1854,91	0,48	0,42	0,42	0,56	0,46	0,39					
1855,91	0,49	0,42	0,43	0,55	0,46	0,40					
1867,91	0,45	0,43	0,46	0,55	0,43	0,39					
1869,91	0,52	0,47	0,44	0,57	0,45	0,39					
1888,91	0,51	0,48	0,46	0,59	0,48	0,37	1888,00	33,26	VLDFEHFLPMLQTVAK	MYL6_HUMAN	Myosin light polypeptide 6
1889,91	0,47	0,44	0,45	0,57	0,46	0,38	1890,03	44,46	NITEIADLTQKIFDLR	TNNI3_HUMAN	Troponin I, cardiac muscle
1922,92	0,49	0,42	0,43	0,57	0,44	0,37					
1923,92	0,52	0,44	0,42	0,60	0,41	0,30					
1924,92	0,49	0,41	0,41	0,60	0,43	0,32					
1925,92	0,48	0,41	0,43	0,57	0,42	0,34					
1961,92	0,49	0,42	0,42	0,59	0,45	0,34					
1962,92	0,50	0,43	0,43	0,60	0,46	0,34					
1963,92	0,49	0,43	0,43	0,58	0,47	0,38					
1964,92	0,47	0,42	0,45	0,60	0,47	0,36					
2003,92	0,51	0,43	0,41	0,62	0,46	0,33					
2004,92	0,53	0,43	0,41	0,58	0,43	0,35					
2005,92	0,50	0,41	0,42	0,56	0,44	0,38					
2041,93	0,52	0,46	0,45	0,56	0,44	0,38					
2056,93	0,49	0,42	0,42	0,58	0,47	0,38					

2057,93	0,54	0,45	0,40	0,59	0,47	0,37					
2072,13	0,46	0,36	0,37	0,60	0,47	0,36					
2073,13	0,44	0,35	0,37	0,59	0,45	0,35					
2074,13	0,50	0,40	0,40	0,58	0,47	0,38					
2079,93	0,51	0,46	0,45	0,56	0,46	0,39					
2088,93	0,51	0,47	0,46	0,56	0,45	0,39	2088,09	51,57	TFGGAPGFPLGSPLSSPVFPR	DESM_HUMAN	Desmin
2089,93	0,54	0,46	0,44	0,57	0,43	0,36					
2094,93	0,48	0,43	0,43	0,60	0,45	0,33					
2095,93	0,49	0,45	0,46	0,59	0,44	0,33					
2096,93	0,49	0,47	0,47	0,58	0,46	0,38					
2104,13	0,44	0,36	0,40	0,62	0,39	0,26					
2105,13	0,44	0,36	0,41	0,62	0,38	0,24					
2105,93	0,49	0,42	0,42	0,57	0,41	0,34					
2106,93	0,50	0,43	0,42	0,55	0,43	0,38					
2115,13	0,48	0,36	0,36	0,61	0,43	0,28					
2116,13	0,49	0,37	0,38	0,61	0,42	0,29					
2117,13	0,49	0,40	0,41	0,59	0,45	0,34					
2118,13	0,52	0,43	0,38	0,60	0,47	0,36					
2154,14	0,44	0,42	0,46	0,56	0,46	0,39					
2163,94	0,49	0,43	0,43	0,60	0,44	0,34					
2164,94	0,53	0,48	0,43	0,59	0,43	0,33					
2165,94	0,52	0,48	0,45	0,57	0,45	0,38					
2199,94	0,50	0,42	0,42	0,57	0,46	0,39	2200,12	49,68	GTLEDQIIQANPALEAFGNAK	MYH6_HUMAN	Myosin-6
2215,94	0,52	0,40	0,36	0,60	0,39	0,29					
2217,14	0,48	0,34	0,34	0,59	0,35	0,26					
2218,14	0,45	0,33	0,37	0,57	0,38	0,31					

2461,17	0,48	0,43	0,46	0,62	0,46	0,33
2462,17	0,47	0,43	0,44	0,61	0,44	0,31
2463,17	0,52	0,46	0,45	0,62	0,45	0,32
2477,17	0,50	0,44	0,43	0,61	0,43	0,31
2478,17	0,47	0,42	0,44	0,61	0,43	0,32
2479,17	0,49	0,45	0,47	0,58	0,44	0,36
2493,17	0,49	0,43	0,42	0,59	0,45	0,35
2675,18	0,50	0,47	0,45	0,60	0,51	0,40
2676,18	0,54	0,49	0,46	0,62	0,50	0,38
2691,19	0,50	0,47	0,46	0,59	0,50	0,39
2692,19	0,51	0,47	0,46	0,59	0,48	0,37
2869,40	0,51	0,39	0,37	0,62	0,43	0,29
2870,40	0,49	0,36	0,37	0,61	0,40	0,26
2871,40	0,47	0,34	0,36	0,61	0,42	0,29
2872,40	0,46	0,36	0,39	0,58	0,43	0,34
2950,41	0,48	0,41	0,42	0,62	0,41	0,27
2951,41	0,47	0,39	0,40	0,62	0,40	0,27
2952,41	0,45	0,37	0,41	0,63	0,40	0,26
2953,41	0,49	0,42	0,41	0,62	0,44	0,31
2960,41	0,43	0,34	0,38	0,60	0,50	0,38
2961,41	0,45	0,35	0,38	0,62	0,52	0,38

Supplementary Table S4. Differential intensity distributions of all peptides (MALDI-MSI) and their corresponding proteins from the tunica adventitia in TAA, AAA and EVAR tissue sections

MALDI-MSI m/z value [Da]	ROC [AUC] Adventitia AAA vs. EVAR	ROC [AUC] Adventitia AAA vs. TAA	ROC [AUC] Adventitia EVAR vs. TAA	LC-MS [MH+Calc.]	Scores	Sequence	Gene Symbol	Protein
1315,66	0,48	0,50	0,53	1316,64	52,08	NELESYAYSLK	HEL-S-89n	78 kDa glucose-regulated protein
1529,68	0,46	0,57	0,59	1528,74	37,32	AKFEELNMDLFR		
976,43	0,52	0,43	0,40	976,45	88,56	AGFAGDDAPR	ACTA2	Actin, aortic smooth muscle
1198,65	0,49	0,38	0,39	1198,52	57,19	DSYVGDEAQSK		
1198,65	0,49	0,38	0,39	1198,70	41,77	AVFPSIVGRPR		
1353,66	0,49	0,48	0,49	1354,62	48,47	DSYVGDEAQSKR		
1580,68	0,54	0,45	0,41	1580,80	43,04	MQKEITALAPSTMK		
1790,90	0,47	0,39	0,42	1790,89	28,06	SYELPDGQVITIGNER		
1342,66	0,48	0,45	0,47	1342,78	32,68	VIQVAAGSSNLKR	ALDH1B1	Aldehyde dehydrogenase, mitochondrial
1230,65	0,44	0,45	0,49	1229,68	27	ELIIGDRQTGK	ATP5F1A	ATP synthase subunit alpha, mitochondrial
1358,66	0,49	0,46	0,48	1358,74	34,89	ISVREPMQTGIK		
1127,64	0,46	0,43	0,47	1126,64	32,56	LEGDTLIIPR	HSPG2	Basement membrane-specific heparan sulfate proteoglycan core protein
1687,69	0,49	0,45	0,46	1687,97	47,44	VGGHLRPGIVQSGGVVR		
1790,90	0,47	0,39	0,42	1790,89	28,06	SYELPDGQVITIGNER	ACTBL2	Beta-actin-like protein 2
2026,93	0,49	0,50	0,50	2027,13	31,14	NHLVEIPPNLPSSLVELR	BGN	Biglycan
1564,88	0,49	0,37	0,38	1564,91	31,49	SIVVSPILIPENQR	CDH13	Cadherin-13
1835,91	0,46	0,37	0,40	1836,84	32,84	MTAFDADDPATDNALLR		
877,02	0,53	0,53	0,51	876,46	59,95	LPASFDAR	CTSB	Cathepsin B

981,43	0,45	0,49	0,53	981,63	32,21	LLLPTPTVK	CCAR1	Cell division cycle and apoptosis regulator protein 1
945,43	0,51	0,46	0,46	945,50	30,15	LSENVIDR	CHCHD10	Coiled-coil-helix-coiled-coil-helix domain-containing protein 3, mitochondrial
836,42	0,52	0,43	0,41	836,44	49,08	GPAGPQGPR	COL1A1	Collagen alpha-1(I) chain
852,42	0,54	0,45	0,42	851,42	43,88	GFSGLDGAK		
868,42	0,50	0,42	0,43	868,43	34,63	GEAGPQGPR		
886,42	0,49	0,40	0,41	886,44	47,39	GSEGPQGVR		
784,41	0,51	0,46	0,45	785,39	48,12	GDQGPVGR	COL1A2	Collagen alpha-2(I) chain
1223,65	0,46	0,45	0,48	1223,61	65,83	GPAGPSGPAGKDGR		
1561,88	0,48	0,38	0,39	1562,79	87,57	GETGPSGPVGPAGAVGPR		
2026,93	0,49	0,50	0,50	2027,01	39,23	HGNRGETGPSGPVGPAGAVGPR		
1214,65	0,47	0,44	0,47	1214,69	44,56	LFAVAPNQNLK	COL6A2	Collagen alpha-2(VI) chain
823,42	0,50	0,50	0,50	823,48	51,16	VGLEHLR	COL6A3	Collagen alpha-3(VI) chain
1237,65	0,52	0,45	0,41	1238,65	47,18	VAVFFSNTPTR		
1459,67	0,54	0,46	0,41	1459,86	25,39	IGDLHPQIVNLLK		
1462,67	0,48	0,44	0,45	1462,76	27,26	QINVGNALEYVSR		
1106,64	0,41	0,36	0,42	1106,68	27,56	VLTLELYKK	CKM	Creatine kinase M-type
1302,66	0,48	0,43	0,43	1302,63	33,03	GQSIDDMIPAQK		
1508,68	0,49	0,41	0,42	1507,80	83,26	LSVEALNSLTGEFK		
1508,68	0,49	0,41	0,42	1507,70	58,36	GGDDLDPNYVLSSR		
906,42	0,51	0,50	0,48	905,52	28,35	LVPQQLAH	COX5B	Cytochrome c oxidase subunit 5B, mitochondrial
1352,66	0,50	0,48	0,47	1352,73	25,67	ELHLDNNKLTR	DCN	Decorin
1032,64	0,43	0,49	0,55	1032,54	88,47	TSGGAGGLGSLR	DES	Desmin
1729,70	0,52	0,48	0,47	1729,88	31,28	VSDLTQAANKNNDALR		
1767,90	0,49	0,39	0,38	1768,83	32,75	DGEVVSEATQQQHEVL		
2088,93	0,51	0,47	0,46	2088,09	51,57	TFGGAPGFPLGSPLSSPVFPR		
816,42	0,45	0,46	0,51	817,41	26,25	DQQIGEK	MCM2	DNA replication licensing factor

								MCM2
853,42	0,52	0,43	0,42	853,52	55,31	LGPLQVAR	ETFB	Electron transfer flavoprotein subunit beta
1340,66	0,50	0,46	0,46	1339,72	51,07	LSVISVEDPPQR		
1134,44	0,53	0,52	0,48	1134,65	42,48	SLNILTAFQK	ERP29	Endoplasmic reticulum resident protein 29
1561,88	0,48	0,38	0,39	1562,82	30,26	PYQYPALTPEQKK	ALDOA	Fructose-bisphosphate aldolase A
2038,93	0,49	0,43	0,45	2038,06	32,53	VTGPEGALFEHSVETPLVR	SGCG	Gamma-sarcoglycan
1066,04	0,55	0,53	0,49	1065,59	31,49	AGAHLQGGAKR	GAPDH	Glyceraldehyde-3-phosphate dehydrogenase
1231,65	0,52	0,50	0,47	1232,64	31,53	AMTKDNNLLGR	HSPA1A	Heat shock 70 kDa protein 1A/1B
1302,66	0,48	0,43	0,43	1303,60	55,78	NALESYAFNMK		
1487,68	0,48	0,43	0,47	1487,70	46,55	TTPSYVAFTDTER		
1231,65	0,52	0,50	0,47	1232,64	31,53	AMTKDNNLLGR	HSPA6	Heat shock 70 kDa protein 6
1487,68	0,48	0,43	0,47	1487,70	46,55	TTPSYVAFTDTER		
1487,68	0,48	0,43	0,47	1487,70	46,55	TTPSYVAFTDTER	HSPA8	Heat shock cognate 71 kDa protein
1487,68	0,48	0,43	0,47	1487,70	46,55	TTPSYVAFTDTER	HSPA2	Heat shock-related 70 kDa protein 2
1529,68	0,46	0,57	0,59	1529,73	45,35	VGAHAGEYGAEALER	HBA1	Hemoglobin subunit alpha
1628,69	0,48	0,44	0,45	1628,78	58,18	SSGPYGGGGQYFAKPR	HNRNPA1	Heterogeneous nuclear ribonucleoprotein A1
944,43	0,53	0,51	0,48	943,57	36,36	VPPPPPIAR	HNRNPC	Heterogeneous nuclear ribonucleoproteins C1/C2
974,43	0,49	0,46	0,44	973,60	45,36	SGVSLAALKK	H1-3	Histone H1.3
1106,64	0,41	0,36	0,42	1107,57	47,87	ALAAAGYDVEK		
1198,65	0,49	0,38	0,39	1198,67	64,14	ASGPPVSELITK		
1326,66	0,44	0,46	0,53	1326,76	42,44	KASGPPVSELITK		
1106,64	0,41	0,36	0,42	1107,57	47,87	ALAAAGYDVEK	H1-6	Histone H1t
1341,66	0,46	0,44	0,46	1341,77	27,41	ALVQNDTLLQVK	H1-10	Histone H1x

944,43	0,53	0,51	0,48	944,53	61,97	AGLQFPVGR	H2AC18	Histone H2A type 2-A
1743,70	0,53	0,43	0,42	1743,82	88,66	AMGIMNSFVNDIFER	H2BC3	Histone H2B type 1-B
688,40	0,51	0,51	0,48	688,43	27,64	KQLATK	H3-4	Histone H3.1t
688,40	0,51	0,51	0,48	688,41	35,49	VTIMPK		
1032,64	0,43	0,49	0,55	1032,59	28,91	YRPGTVALR		
1325,66	0,45	0,45	0,48	1325,75	45,61	DNIQGITKPAIR	H4C1	Histone H4
1466,67	0,55	0,43	0,38	1466,80	61,03	TVTAMDVVYALKR		
1481,68	0,47	0,43	0,46	1481,85	29,78	DNIQGITKPAIRR		
852,42	0,54	0,45	0,42	851,44	26,56	DTLMISR	IGHG1	Ig gamma-1 chain C region
1710,90	0,46	0,41	0,42	1710,81	25,14	FEAAETLEEAAMRSR	KLC1	Kinesin light chain 1
1848,91	0,46	0,39	0,43	1848,87	52,86	AGNSLAASTAEETAGSAQGR	LAMB2	Laminin subunit beta-2
1324,66	0,54	0,48	0,43	1324,71	33,76	SASYNLSLTLQK	LDB3	LIM domain-binding protein 3
1648,69	0,54	0,50	0,45	1648,88	61,58	GGPAYTPAGPQVPPLAR		
1303,66	0,48	0,39	0,40	1303,68	41,21	PMFIVNTNVPR	MIF	Macrophage migration inhibitory factor
644,00	0,53	0,54	0,52	643,41	33,2	AQIALK	MDH1	Malate dehydrogenase, cytoplasmic
1060,04	0,51	0,53	0,53	1060,54	34,27	DFADIPNLR	OGN	Mimecan
2027,93	0,53	0,52	0,45	2027,97	32,96	TETTMSPLTNTTTSQGTTR	MUC5B	Mucin-5B
877,02	0,53	0,53	0,51	876,53	44,21	ALELFRK	MB	Myoglobin
1478,68	0,55	0,47	0,43	1478,90	70,53	HGATVLTALGGILKK		
1264,46	0,48	0,48	0,50	1263,60	47,85	ESNGTVMGAELR	MYL4	Myosin light chain 4
1888,91	0,51	0,48	0,46	1888,00	33,26	VLDFEHFLPMLQTVAK	MYL6	Myosin light polypeptide 6
1060,04	0,51	0,53	0,53	1060,51	50,2	ANSEVAQWR	MYH13	Myosin-13
1060,04	0,51	0,53	0,53	1060,51	50,2	ANSEVAQWR	MYH3	Myosin-3
1060,04	0,51	0,53	0,53	1060,51	50,2	ANSEVAQWR	MYO6	Myosin-6
1082,04	0,51	0,52	0,51	1081,53	50,48	SLNDFTTQR		
1533,68	0,55	0,47	0,42	1533,78	82,04	VVDSLQTSLDAETR		
1768,90	0,47	0,39	0,42	1768,96	66,66	ILNPVAIPEGQFIDSR		

1850,91	0,52	0,46	0,44	1851,04	48,84	VQLLHSQNTSLINQKK		
1966,92	0,49	0,48	0,51	1967,02	30,33	DLEEATLQHEATAAALRK		
2088,93	0,51	0,47	0,46	2088,12	33,09	YRILNPVAIPEGQFIDSR		
2199,94	0,50	0,42	0,42	2200,12	49,68	GTLEDQIIQANPALEAFGNAK		
1060,04	0,51	0,53	0,53	1060,51	50,2	ANSEVAQWR	MYH7	Myosin-7
1533,68	0,55	0,47	0,42	1533,78	82,04	VVDSLQTSLDAETR		
1850,91	0,52	0,46	0,44	1851,04	48,84	VQLLHSQNTSLINQKK		
1966,92	0,49	0,48	0,51	1967,02	30,33	DLEEATLQHEATAAALRK		
2199,94	0,50	0,42	0,42	2200,12	49,68	GTLEDQIIQANPALEAFGNAK		
1848,91	0,46	0,39	0,43	1848,90	37,01	SVEVAAGSPAVFEAETER	MYBPC3	Myosin-binding protein C, cardiac-type
1572,68	0,49	0,49	0,52	1572,77	49,39	DIMLEELSHLSNR	MYOZ2	Myozenin-2
1723,70	0,51	0,48	0,46	1723,86	47,13	SPPNPDNIAPGYSGPLK		
1359,66	0,52	0,47	0,47	1359,67	25,52	LLEEENQESLR	NES	Nestin
1094,64	0,52	0,40	0,37	1095,56	25,48	KITIADCGQF	PPIAL4A	Peptidyl-prolyl cis-trans isomerase A-like 4A/B/C
1106,64	0,41	0,36	0,42	1107,60	25,88	TIAQDYGVLK	PRDX1	Peroxiredoxin-1
1096,64	0,51	0,43	0,40	1097,62	51,55	VLPGVDALSNI	PGK1	Phosphoglycerate kinase 1
976,43	0,52	0,43	0,40	975,49	36,63	AMEAVAAQGK	PGAM2	Phosphoglycerate mutase 2
1150,65	0,49	0,49	0,50	1150,67	45,55	VLIAAHGNSLR		
976,43	0,52	0,43	0,40	976,45	88,56	AGFAGDDAPR	POTEE	POTE ankyrin domain family member E
1199,65	0,51	0,42	0,41	1198,71	33,51	AVFPSIVGRPR		
1790,90	0,47	0,39	0,42	1790,89	28,06	SYELPDGQVITIGNER		
1967,92	0,47	0,48	0,51	1968,08	25,89	VAPEEHPILLTEAPLNPK		
976,43	0,52	0,43	0,40	976,45	88,56	AGFAGDDAPR	POTEF	POTE ankyrin domain family member F
1199,65	0,51	0,42	0,41	1198,71	33,51	AVFPSIVGRPR		
1790,90	0,47	0,39	0,42	1790,89	28,06	SYELPDGQVITIGNER		
976,43	0,52	0,43	0,40	976,45	88,56	AGFAGDDAPR	POTEI	POTE ankyrin domain family

								member I
1199,65	0,51	0,42	0,41	1198,71	33,51	AVFPSIVGRPR		
1967,92	0,47	0,48	0,51	1968,08	25,89	VAPEEHPILLTEAPLNPK		
976,43	0,52	0,43	0,40	976,45	88,56	AGFAGDDAPR	POTEJ	POTE ankyrin domain family member J
1967,92	0,47	0,48	0,51	1968,08	25,89	VAPEEHPILLTEAPLNPK		
1565,88	0,45	0,38	0,42	1566,75	43,84	SVGGSGGGSFGDNLVTR	LMNA	Prelamin-A/C
1564,88	0,49	0,37	0,38	1565,86	29,41	NLMQLNLAHNILR	PRELP	Prolargin
1790,90	0,47	0,39	0,42	1790,89	28,06	SYELPDGQVITIGNER	POTEKP	Putative beta-actin-like protein 3
1967,92	0,47	0,48	0,51	1968,08	25,89	VAPEEHPILLTEAPLNPK		
1461,67	0,50	0,42	0,41	1461,75	27,91	ISPEEVYNNLKR	NSUN7	Putative methyltransferase NSUN7
1462,67	0,48	0,44	0,45	1461,75	27,91	ISPEEVYNNLKR		
840,42	0,47	0,45	0,48	840,47	26,42	RLNNAPR	SHISA8	Putative protein shisa-8
1321,66	0,55	0,47	0,42	1321,74	51,46	DEILLLHQAAAK	SLMAP	Sarcolemmal membrane-associated protein
1391,67	0,46	0,48	0,51	1391,72	25,9	AEIGIAMGSGTAVAK	ATP2A2	Sarcoplasmic/endoplasmic reticulum calcium ATPase 2
1776,90	0,49	0,40	0,42	1777,87	33,2	SDGDPVQPAVLQVHQTS	SDPR	Serum deprivation-response protein
1107,64	0,50	0,40	0,40	1108,59	32,94	ITALDEFATK	Sptan1	Spectrin alpha chain, non-erythrocytic 1
899,42	0,54	0,47	0,42	899,55	25,53	AAVVALSLR	SDHB	Succinate dehydrogenase [ubiquinone] iron-sulfur subunit, mitochondrial
1302,66	0,48	0,43	0,43	1302,74	29,55	SLRFPLALEEK	TTN	Titin
1315,66	0,48	0,50	0,53	1315,72	29,24	AGEDVQVLIPFK		
1602,69	0,52	0,47	0,44	1602,89	31,2	VVLAYEPVWAIGTGK	TPI1	Triosephosphate isomerase
875,42	0,51	0,48	0,46	875,45	30,27	SLEAQAEK	TPM1	Tropomyosin alpha-1 chain
1314,66	0,46	0,49	0,55	1314,76	40,4	KLVIIESDLER		

1460,67	0,56	0,47	0,40	1460,73	39,4	KATDAEADVASLNR		
1488,68	0,54	0,48	0,43	1488,74	27,75	ATDAEADVASLNRR		
1516,68	0,52	0,45	0,42	1516,82	27,34	SKQLEDELVSLQK		
875,42	0,51	0,48	0,46	875,45	30,27	SLEAQAEK	TPM3	Tropomyosin alpha-3 chain
1460,67	0,56	0,47	0,40	1460,73	39,4	KATDAEADVASLNR	TPM2	Tropomyosin beta chain
1488,68	0,54	0,48	0,43	1488,74	27,75	ATDAEADVASLNRR		
1305,66	0,47	0,44	0,45	1306,64	44,38	KNIDALSGMEGR	TNNI3	Troponin I, cardiac muscle
1479,68	0,48	0,44	0,47	1479,73	42,27	ISADAMMQALLGAR		
1889,91	0,47	0,44	0,45	1890,03	44,46	NITEIADLTQKIFDLR		
758,41	0,50	0,45	0,46	757,47	35,39	ILAERR	TNNT2	Troponin T, cardiac muscle
906,42	0,51	0,50	0,48	906,50	26,65	YEINVLR		
1797,90	0,49	0,46	0,44	1796,93	29,11	SFMPNLVPPKIPDGER		
1143,45	0,52	0,51	0,49	1143,63	28,94	LAVNMVPFPR	TUBB	Tubulin beta chain
1320,66	0,57	0,48	0,41	1319,70	49,82	IMNTFSVVPSPK		
1620,69	0,52	0,45	0,44	1620,83	34,82	LHFFMPGFAPLTSR		
1143,45	0,52	0,51	0,49	1143,63	28,94	LAVNMVPFPR	TUBB1	Tubulin beta-1 chain
1143,45	0,52	0,51	0,49	1143,63	28,94	LAVNMVPFPR	TUBB3	Tubulin beta-3 chain
1320,66	0,57	0,48	0,41	1319,70	49,82	IMNTFSVVPSPK		
1143,45	0,52	0,51	0,49	1143,63	28,94	LAVNMVPFPR	TUBB4B	Tubulin beta-4B chain
1320,66	0,57	0,48	0,41	1319,70	49,82	IMNTFSVVPSPK		
1602,69	0,52	0,47	0,44	1601,82	30,14	AVLVDLEPGTMDSVR		
1620,69	0,52	0,45	0,44	1620,83	34,82	LHFFMPGFAPLTSR		
1143,45	0,52	0,51	0,49	1143,63	28,94	LAVNMVPFPR	TUBB6	Tubulin beta-6 chain
1620,69	0,52	0,45	0,44	1620,83	34,82	LHFFMPGFAPLTSR		
1143,45	0,52	0,51	0,49	1143,63	28,94	LAVNMVPFPR	TUBB8	Tubulin beta-8 chain
1602,69	0,52	0,47	0,44	1601,82	30,14	AVLVDLEPGTMDSVR		
1620,69	0,52	0,45	0,44	1620,83	34,82	LHFFMPGFAPLTSR		
1320,66	0,57	0,48	0,41	1320,71	38,4	TSAALSTVGSAISR	TPD52L2	Tumor protein D54
1269,66	0,52	0,45	0,42	1270,56	37,39	LGDLYEEEMR	VIM	Vimentin
1428,67	0,50	0,44	0,44	1428,71	40,41	SLYASSPGGVYATR		

2498,17	0,50	0,46	0,45	2497,26	42,86	LLQDSVDFSLADAINTEFKNTR		
1503,68	0,50	0,49	0,48	1503,83	25,29	IYISGMAPRPSLAK	VTN	Vitronectin

Supplementary Table S5. Differential intensity distributions of all peptides (MALDI-MSI) and their corresponding proteins from the tunica media in TAA, AAA and EVAR tissue sections

MALDI-MSI m/z value [Da]	ROC [AUC] Media AAA vs. EVAR	ROC [AUC] Media AAA vs. TAA	ROC [AUC] Media EVAR vs. TAA	LC-MS [MH+Calc.]	Scores	Sequence	Gene Symbol	Protein
1315,66	0,51	0,42	0,42	1316,64	52,08	NELESYAYSLK	HEL-S-89n	78 kDa glucose-regulated protein
1529,68	0,50	0,61	0,60	1528,74	37,32	AKFEELNMDLFR		
976,43	0,62	0,35	0,22	976,45	88,56	AGFAGDDAPR	ACTA2	Actin, aortic smooth muscle
1198,65	0,66	0,29	0,10	1198,52	57,19	DSYVGDEAQSK		
1198,65	0,66	0,29	0,10	1198,70	41,77	AVFPSIVGRPR		
1353,66	0,51	0,48	0,47	1354,62	48,47	DSYVGDEAQSKR		
1580,68	0,56	0,45	0,39	1580,80	43,04	MQKEITALAPSTMK		
1790,90	0,57	0,36	0,27	1790,89	28,06	SYELPDGQVITIGNER		
1342,66	0,57	0,43	0,35	1342,78	32,68	VIQVAAGSSNLKR	ALDH1B1	Aldehyde dehydrogenase, mitochondrial
1230,65	0,53	0,46	0,43	1229,68	27	ELIIGDRQTGK	ATP5F1A	ATP synthase subunit alpha, mitochondrial
1358,66	0,60	0,41	0,30	1358,74	34,89	ISVREPMQTGIK		
1127,64	0,56	0,47	0,42	1126,64	32,56	LEGDTLIIPR	HSPG2	Basement membrane-specific heparan sulfate proteoglycan core protein
1687,69	0,56	0,44	0,38	1687,97	47,44	VGGHLRPGIVQSGGVVR		
1790,90	0,57	0,36	0,27	1790,89	28,06	SYELPDGQVITIGNER	ACTBL2	Beta-actin-like protein 2
2026,93	0,55	0,53	0,48	2027,13	31,14	NHLVEIPPNLPSSLVELR	BGN	Biglycan
1564,88	0,59	0,42	0,33	1564,91	31,49	SIVVSPILIPENQR	CDH13	Cadherin-13
1835,91	0,58	0,44	0,34	1836,84	32,84	MTAFDADDPATDNALLR		
877,02	0,51	0,52	0,51	876,46	59,95	LPASFDAR	CTSB	Cathepsin B
981,43	0,45	0,50	0,54	981,63	32,21	LLLPTPTVK	CCAR1	Cell division cycle and apoptosis

								regulator protein 1
945,43	0,56	0,45	0,38	945,50	30,15	LSENVIDR	CHCHD10	Coiled-coil-helix-coiled-coil-helix domain-containing protein 3, mitochondrial
836,42	0,59	0,42	0,31	836,44	49,08	GPAGPQGPR	COL1A1	Collagen alpha-1(I) chain
852,42	0,61	0,40	0,28	851,42	43,88	GFSGLDGAK		
868,42	0,59	0,42	0,31	868,43	34,63	GEAGPQGPR		
886,42	0,56	0,43	0,36	886,44	47,39	GSEGPQGVR		
784,41	0,58	0,45	0,37	785,39	48,12	GDQGPVGR	COL1A2	Collagen alpha-2(I) chain
1223,65	0,53	0,47	0,45	1223,61	65,83	GPAGPSGPAGKDGR		
1561,88	0,60	0,48	0,37	1562,79	87,57	GETGPSGPVGPAGAVGPR		
2026,93	0,55	0,53	0,48	2027,01	39,23	HGNRGETGPSGPVGPAGAVGPR		
1214,65	0,54	0,46	0,42	1214,69	44,56	LFAVAPNQNLK	COL6A2	Collagen alpha-2(VI) chain
823,42	0,54	0,46	0,43	823,48	51,16	VGLEHLR	COL6A3	Collagen alpha-3(VI) chain
1237,65	0,57	0,43	0,37	1238,65	47,18	VAVFFSNTPTR		
1459,67	0,61	0,43	0,32	1459,86	25,39	IGDLHPQIVNLLK		
1462,67	0,55	0,44	0,39	1462,76	27,26	QINVGNALEYVSR		
1106,64	0,58	0,42	0,32	1106,68	27,56	VLTLELYKK	CKM	Creatine kinase M-type
1302,66	0,57	0,48	0,41	1302,63	33,03	GQSIDDMIPAQK		
1508,68	0,60	0,40	0,32	1507,80	83,26	LSVEALNSLTGEFK		
1508,68	0,60	0,40	0,32	1507,70	58,36	GGDDLDPNYVLSSR		
906,42	0,55	0,43	0,37	905,52	28,35	LVPQQLAH	COX5B	Cytochrome c oxidase subunit 5B, mitochondrial
1352,66	0,52	0,44	0,43	1352,73	25,67	ELHLDNNKLTR	DCN	Decorin
1032,64	0,46	0,49	0,53	1032,54	88,47	TSGGAGGLGSLR	DES	Desmin
1729,70	0,55	0,45	0,40	1729,88	31,28	VSDLTQAANKNNDALR		
1767,90	0,57	0,47	0,39	1768,83	32,75	DGEVVSEATQQQHEVL		
2088,93	0,56	0,45	0,39	2088,09	51,57	TFGGAPGFPLGSPLSSPVFPR		
816,42	0,53	0,46	0,42	817,41	26,25	DQQIGEK	MCM2	DNA replication licensing factor MCM2

853,42	0,61	0,40	0,27	853,52	55,31	LGPLQVAR	ETFB	Electron transfer flavoprotein subunit beta
1340,66	0,60	0,44	0,34	1339,72	51,07	LSVISVEDPPQR		
1134,44	0,56	0,43	0,38	1134,65	42,48	SLNILTAFQK	ERP29	Endoplasmic reticulum resident protein 29
1561,88	0,60	0,48	0,37	1562,82	30,26	PYQYPALTPEQKK	ALDOA	Fructose-bisphosphate aldolase A
2038,93	0,53	0,47	0,44	2038,06	32,53	VTGPEGALFEHSVETPLVR	SGCG	Gamma-sarcoglycan
1066,04	0,57	0,48	0,42	1065,59	31,49	AGAHLQGGAKR	GAPDH	Glyceraldehyde-3-phosphate dehydrogenase
1231,65	0,58	0,44	0,35	1232,64	31,53	AMTKDNNLLGR	HSPA1A	Heat shock 70 kDa protein 1A/1B
1302,66	0,57	0,48	0,41	1303,60	55,78	NALESYAFNMK		
1487,68	0,55	0,46	0,42	1487,70	46,55	TTPSYVAFTDTER		
1231,65	0,58	0,44	0,35	1232,64	31,53	AMTKDNNLLGR	HSPA6	Heat shock 70 kDa protein 6
1487,68	0,55	0,46	0,42	1487,70	46,55	TTPSYVAFTDTER		
1487,68	0,55	0,46	0,42	1487,70	46,55	TTPSYVAFTDTER	HSPA8	Heat shock cognate 71 kDa protein
1487,68	0,55	0,46	0,42	1487,70	46,55	TTPSYVAFTDTER	HSPA2	Heat shock-related 70 kDa protein 2
1529,68	0,50	0,61	0,60	1529,73	45,35	VGAHAGEYGAEALER	HBA1	Hemoglobin subunit alpha
1628,69	0,51	0,44	0,43	1628,78	58,18	SSGPYGGGGQYFAKPR	HNRNPA1	Heterogeneous nuclear ribonucleoprotein A1
944,43	0,57	0,45	0,37	943,57	36,36	VPPPPPIAR	HNRNPC	Heterogeneous nuclear ribonucleoproteins C1/C2
974,43	0,56	0,43	0,38	973,60	45,36	SGVSLAALKK	H1-3	Histone H1.3
1106,64	0,58	0,42	0,32	1107,57	47,87	ALAAAGYDVEK		
1198,65	0,66	0,29	0,10	1198,67	64,14	ASGPPVSELITK		
1326,66	0,54	0,46	0,41	1326,76	42,44	KASGPPVSELITK		
1106,64	0,58	0,42	0,32	1107,57	47,87	ALAAAGYDVEK	H1-6	Histone H1t
1341,66	0,58	0,42	0,34	1341,77	27,41	ALVQNDTLLQVK	H1-10	Histone H1x
944,43	0,57	0,45	0,37	944,53	61,97	AGLQFPVGR	H2AC18	Histone H2A type 2-A
1743,70	0,59	0,47	0,38	1743,82	88,66	AMGIMNSFVNDIFER	H2BC3	Histone H2B type 1-B

688,40	0,61	0,44	0,33	688,43	27,64	KQLATK	H3-4	Histone H3.1t
688,40	0,61	0,44	0,33	688,41	35,49	VTIMPK		
1032,64	0,46	0,49	0,53	1032,59	28,91	YRPGTVALR		
1325,66	0,59	0,44	0,36	1325,75	45,61	DNIQGITKPAIR	H4C1	Histone H4
1466,67	0,60	0,42	0,32	1466,80	61,03	TVTAMDVVYALKR		
1481,68	0,54	0,46	0,42	1481,85	29,78	DNIQGITKPAIRR		
852,42	0,61	0,40	0,28	851,44	26,56	DTLMISR	IGHG1	Ig gamma-1 chain C region
1710,90	0,60	0,36	0,26	1710,81	25,14	FEAAETLEEAAMRSR	KLC1	Kinesin light chain 1
1848,91	0,56	0,46	0,41	1848,87	52,86	AGNSLAASTAEETAGSAQGR	LAMB2	Laminin subunit beta-2
1324,66	0,59	0,43	0,34	1324,71	33,76	SASYNLSLTLQK	LDB3	LIM domain-binding protein 3
1648,69	0,54	0,48	0,44	1648,88	61,58	GGPAYTPAGPQVPPLAR		
1303,66	0,62	0,36	0,24	1303,68	41,21	PMFIVNTNVPR	MIF	Macrophage migration inhibitory factor
644,00	0,54	0,49	0,43	643,41	33,2	AQIALK	MDH1	Malate dehydrogenase, cytoplasmic
1060,04	0,53	0,49	0,42	1060,54	34,27	DFADIPNLR	OGN	Mimecan
2027,93	0,57	0,52	0,45	2027,97	32,96	TETTMSPLTNTTTSQGTTR	MUC5B	Mucin-5B
877,02	0,51	0,52	0,51	876,53	44,21	ALELFRK	MB	Myoglobin
1478,68	0,59	0,45	0,35	1478,90	70,53	HGATVLTALGGILKK		
1264,46	0,48	0,51	0,53	1263,60	47,85	ESNGTVMGAELR	MYL4	Myosin light chain 4
1888,91	0,59	0,48	0,37	1888,00	33,26	VLDFEHFLPMLQTVAK	MYL6	Myosin light polypeptide 6
1060,04	0,53	0,49	0,42	1060,51	50,2	ANSEVAQWR	MYH13	Myosin-13
1060,04	0,53	0,49	0,42	1060,51	50,2	ANSEVAQWR	MYH3	Myosin-3
1060,04	0,53	0,49	0,42	1060,51	50,2	ANSEVAQWR	MYO6	Myosin-6
1082,04	0,51	0,52	0,52	1081,53	50,48	SLNDFTTQR		
1533,68	0,58	0,44	0,34	1533,78	82,04	VVDSLQTSLDAETR		
1768,90	0,54	0,47	0,42	1768,96	66,66	ILNPVAIPEGQFIDSR		
1850,91	0,59	0,44	0,34	1851,04	48,84	VQLLHSQNTSLINQKK		
1966,92	0,56	0,48	0,41	1967,02	30,33	DLEEATLQHEATAAALRK		
2088,93	0,56	0,45	0,39	2088,12	33,09	YRILNPVAIPEGQFIDSR		
2199,94	0,57	0,46	0,39	2200,12	49,68	GTLEDQIIQANPALEAFGNAK		

1060,04	0,53	0,49	0,42	1060,51	50,2	ANSEVAQWR	MYH7	Myosin-7
1533,68	0,58	0,44	0,34	1533,78	82,04	VVDSLQTSLDAETR		
1850,91	0,59	0,44	0,34	1851,04	48,84	VQLLHSQNTSLINQKK		
1966,92	0,56	0,48	0,41	1967,02	30,33	DLEEATLQHEATAAALRK		
2199,94	0,57	0,46	0,39	2200,12	49,68	GTLEDQIIQANPALEAFGNAK		
1848,91	0,56	0,46	0,41	1848,90	37,01	SVEVAAGSPAVFEAETER	MYBPC3	Myosin-binding protein C, cardiac-type
1572,68	0,52	0,47	0,46	1572,77	49,39	DIMLEELSHLSNR	MYOZ2	Myozenin-2
1723,70	0,57	0,42	0,35	1723,86	47,13	SPPNPDNIAPGYSGPLK		
1359,66	0,63	0,40	0,26	1359,67	25,52	LLEEENQESLR	NES	Nestin
1094,64	0,63	0,37	0,23	1095,56	25,48	KITIADCGQF	PPIAL4A	Peptidyl-prolyl cis-trans isomerase A-like 4A/B/C
1106,64	0,58	0,42	0,32	1107,60	25,88	TIAQDYGVLK	PRDX1	Peroxiredoxin-1
1096,64	0,59	0,42	0,32	1097,62	51,55	VLPGVDALSNI	PGK1	Phosphoglycerate kinase 1
976,43	0,62	0,35	0,22	975,49	36,63	AMEAVAAQGK	PGAM2	Phosphoglycerate mutase 2
1150,65	0,56	0,43	0,38	1150,67	45,55	VLIAAHGNSLR		
976,43	0,62	0,35	0,22	976,45	88,56	AGFAGDDAPR	POTEE	POTE ankyrin domain family member E
1199,65	0,65	0,30	0,12	1198,71	33,51	AVFPSIVGRPR		
1790,90	0,57	0,36	0,27	1790,89	28,06	SYELPDGQVITIGNER		
1967,92	0,51	0,47	0,46	1968,08	25,89	VAPEEHPILLTEAPLNPK		
976,43	0,62	0,35	0,22	976,45	88,56	AGFAGDDAPR	POTEF	POTE ankyrin domain family member F
1199,65	0,65	0,30	0,12	1198,71	33,51	AVFPSIVGRPR		
1790,90	0,57	0,36	0,27	1790,89	28,06	SYELPDGQVITIGNER		
976,43	0,62	0,35	0,22	976,45	88,56	AGFAGDDAPR	POTEI	POTE ankyrin domain family member I
1199,65	0,65	0,30	0,12	1198,71	33,51	AVFPSIVGRPR		
1967,92	0,51	0,47	0,46	1968,08	25,89	VAPEEHPILLTEAPLNPK		
976,43	0,62	0,35	0,22	976,45	88,56	AGFAGDDAPR	POTEJ	POTE ankyrin domain family

								member J
1967,92	0,51	0,47	0,46	1968,08	25,89	VAPEEHPILLTEAPLNPK		
1565,88	0,54	0,44	0,40	1566,75	43,84	SVGGSGGGSFGDNLVTR	LMNA	Prelamin-A/C
1564,88	0,59	0,42	0,33	1565,86	29,41	NLMQLNLAHNILR	PRELP	Prolargin
1790,90	0,57	0,36	0,27	1790,89	28,06	SYELPDGQVITIGNER	POTEKP	Putative beta-actin-like protein 3
1967,92	0,51	0,47	0,46	1968,08	25,89	VAPEEHPILLTEAPLNPK		
1461,67	0,58	0,42	0,33	1461,75	27,91	ISPEEVYNNLKR	NSUN7	Putative methyltransferase NSUN7
840,42	0,59	0,48	0,38	840,47	26,42	RLNNAPR	SHISA8	Putative protein shisa-8
1321,66	0,63	0,37	0,24	1321,74	51,46	DEILLLHQAAAK	SLMAP	Sarcolemmal membrane-associated protein
1391,67	0,46	0,48	0,52	1391,72	25,9	AEIGIAMGSGTAVAK	ATP2A2	Sarcoplasmic/endoplasmic reticulum calcium ATPase 2
1776,90	0,59	0,46	0,35	1777,87	33,2	SDGDPVQPAVLQVHQTS	SDPR	Serum deprivation-response protein
1107,64	0,61	0,44	0,31	1108,59	32,94	ITALDEFATK	Sptan1	Spectrin alpha chain, non-erythrocytic 1
899,42	0,56	0,46	0,39	899,55	25,53	AAVVALSLR	SDHB	Succinate dehydrogenase [ubiquinone] iron-sulfur subunit, mitochondrial
1302,66	0,57	0,48	0,41	1302,74	29,55	SLRFPLALEEK	TTN	Titin
1315,66	0,51	0,42	0,42	1315,72	29,24	AGEDVQVLIPFK		
1602,69	0,56	0,44	0,37	1602,89	31,2	VVLAYEPVWAIGTGK	TPI1	Triosephosphate isomerase
875,42	0,57	0,44	0,37	875,45	30,27	SLEAQAEK	TPM1	Tropomyosin alpha-1 chain
1314,66	0,50	0,41	0,42	1314,76	40,4	KLVIIESDLER		
1460,67	0,62	0,43	0,31	1460,73	39,4	KATDAEADVASLNR		
1488,68	0,55	0,45	0,41	1488,74	27,75	ATDAEADVASLNRR		
1516,68	0,61	0,43	0,32	1516,82	27,34	SKQLEDELVSLQK		
875,42	0,57	0,44	0,37	875,45	30,27	SLEAQAEK	TPM3	Tropomyosin alpha-3 chain
1460,67	0,62	0,43	0,31	1460,73	39,4	KATDAEADVASLNR	TPM2	Tropomyosin beta chain
1488,68	0,55	0,45	0,41	1488,74	27,75	ATDAEADVASLNRR		

1305,66	0,58	0,41	0,34	1306,64	44,38	KNIDALSGMEGR	TNNI3	Troponin I, cardiac muscle
1479,68	0,54	0,43	0,40	1479,73	42,27	ISADAMMQALLGAR		
1889,91	0,57	0,46	0,38	1890,03	44,46	NITEIADLTQKIFDLR		
758,41	0,55	0,45	0,40	757,47	35,39	ILAERR	TNNT2	Troponin T, cardiac muscle
906,42	0,55	0,43	0,37	906,50	26,65	YEINVLR		
1797,90	0,57	0,46	0,40	1796,93	29,11	SFMPNLVPPKIPDGER		
1143,45	0,60	0,42	0,31	1143,63	28,94	LAVNMVPFPR	TUBB	Tubulin beta chain
1320,66	0,64	0,38	0,24	1319,70	49,82	IMNTFSVVPSPK		
1621,69	0,52	0,47	0,46	1620,83	34,82	LHFFMPGFAPLTSR		
1143,45	0,60	0,42	0,31	1143,63	28,94	LAVNMVPFPR	TUBB1	Tubulin beta-1 chain
1143,45	0,60	0,42	0,31	1143,63	28,94	LAVNMVPFPR	TUBB3	Tubulin beta-3 chain
1320,66	0,64	0,38	0,24	1319,70	49,82	IMNTFSVVPSPK		
1143,45	0,60	0,42	0,31	1143,63	28,94	LAVNMVPFPR	TUBB4B	Tubulin beta-4B chain
1320,66	0,64	0,38	0,24	1319,70	49,82	IMNTFSVVPSPK		
1602,69	0,56	0,44	0,37	1601,82	30,14	AVLVDLEPGTMDSVR		
1620,69	0,54	0,47	0,43	1620,83	34,82	LHFFMPGFAPLTSR		
1143,45	0,60	0,42	0,31	1143,63	28,94	LAVNMVPFPR	TUBB6	Tubulin beta-6 chain
1620,69	0,54	0,47	0,43	1620,83	34,82	LHFFMPGFAPLTSR		
1143,45	0,60	0,42	0,31	1143,63	28,94	LAVNMVPFPR	TUBB8	Tubulin beta-8 chain
1602,69	0,56	0,44	0,37	1601,82	30,14	AVLVDLEPGTMDSVR		
1620,69	0,54	0,47	0,43	1620,83	34,82	LHFFMPGFAPLTSR		
1320,66	0,64	0,38	0,24	1320,71	38,4	TSAALSTVGSAISR	TPD52L2	Tumor protein D54
1269,66	0,56	0,43	0,37	1270,56	37,39	LGDLYEEEMR	VIM	Vimentin
1428,67	0,55	0,33	0,28	1428,71	40,41	SLYASSPGGVYATR		
2498,17	0,55	0,49	0,44	2497,26	42,86	LLQDSVDFSLADAINTEFKNTR		
1503,68	0,56	0,46	0,40	1503,83	25,29	IYISGMAPRPSLAK	VTN	Vitronectin
